# Diagnostic performance of artificial intelligence-assisted PET imaging for Parkinson’s disease: a systematic review and meta-analysis

**DOI:** 10.1038/s41746-024-01012-z

**Published:** 2024-01-22

**Authors:** Jing Wang, Le Xue, Jiehui Jiang, Fengtao Liu, Ping Wu, Jiaying Lu, Huiwei Zhang, Weiqi Bao, Qian Xu, Zizhao Ju, Li Chen, Fangyang Jiao, Huamei Lin, Jingjie Ge, Chuantao Zuo, Mei Tian

**Affiliations:** 1 Huashan Hospital & Human Phenome Institute, Fudan University, Shanghai, China; 2 Department of Nuclear Medicine/PET Center, Huashan Hospital, Fudan University, Shanghai, China; 3 Department of Nuclear Medicine, the Second Hospital of Zhejiang University School of Medicine, Hangzhou, Zhejiang China; 4 Institute of Biomedical Engineering, School of Life Science, Shanghai University, Shanghai, China; 5 Department of Neurology, Huashan Hospital, Fudan University, Shanghai, China; 6 National Clinical Research Center for Aging and Medicine, & National Center for Neurological Disorders, Huashan Hospital, Fudan University, Shanghai, China; 7 Department of Ultrasound Medicine, Huashan Hospital, Fudan University, Shanghai, China

**Keywords:** Parkinson's disease, Computational science, Brain imaging, Molecular imaging

## Abstract

Artificial intelligence (AI)-assisted PET imaging is emerging as a promising tool for the diagnosis of Parkinson’s disease (PD). We aim to systematically review the diagnostic accuracy of AI-assisted PET in detecting PD. The Ovid MEDLINE, Ovid Embase, Web of Science, and IEEE Xplore databases were systematically searched for related studies that developed an AI algorithm in PET imaging for diagnostic performance from PD and were published by August 17, 2023. Binary diagnostic accuracy data were extracted for meta-analysis to derive outcomes of interest: area under the curve (AUC). 23 eligible studies provided sufficient data to construct contingency tables that allowed the calculation of diagnostic accuracy. Specifically, 11 studies were identified that distinguished PD from normal control, with a pooled AUC of 0.96 (95% CI: 0.94–0.97) for presynaptic dopamine (DA) and 0.90 (95% CI: 0.87–0.93) for glucose metabolism (^18^F-FDG). 13 studies were identified that distinguished PD from the atypical parkinsonism (AP), with a pooled AUC of 0.93 (95% CI: 0.91 − 0.95) for presynaptic DA, 0.79 (95% CI: 0.75–0.82) for postsynaptic DA, and 0.97 (95% CI: 0.96–0.99) for ^18^F-FDG. Acceptable diagnostic performance of PD with AI algorithms-assisted PET imaging was highlighted across the subgroups. More rigorous reporting standards that take into account the unique challenges of AI research could improve future studies.

## Introduction

Parkinson’s disease (PD) is the most common neurodegenerative disorder associated with involuntary or uncontrollable movements^1^. In addition to these motor symptoms, patients with progressive disease may also experience other complications such as cognitive impairment, mental and behavioral disorders, sleep disorders, memory problems, and sensory disturbances^2^. Accurate diagnosis in the early clinical or prodromal stages, however, remains a challenge due to symptom overlap with conditions like atypical parkinsonism (AP)^3^. An estimated 20-30% of patients initially diagnosed with PD are, post-pathological examinations, reclassified as having either multiple system atrophy (MSA) or progressive supranuclear palsy (PSP)^4^. This misdiagnosis affects clinical care and research trials by leading to incorrect prognoses, heterogeneous therapeutic responses in PD and AP^5^. Hence, it is essential to establish precise diagnoses early, considering the symptom similarity but differing treatment requirements across these conditions^6^.

In addition to diagnosing PD on the basis of the above general symptoms examined by clinicians, imaging techniques, particularly PET molecular imaging, which are used as critical imaging biomarkers for diagnosis and disease progression by clinicians and researchers in the PD progression, reveal a wide range of neurobiological abnormalities and have shown to be helpful in the differential diagnosis of parkinsonism to facilitate decision making for diagnosis and treatment^3,7^. The ^18^Flourine-fluorodeoxyglucose (^18^F-FDG) PET scan offers comprehensive insights into brain glucose metabolism, assisting in differentiating PD from other neurodegenerative conditions through distinctive glucose metabolism patterns^8^. Further, dopaminergic imaging evaluates the condition of dopamine (DA) neurons, providing tangible evidence of the dopaminergic system’s dysfunction, a key feature of PD^9^. Reading these imaging results accurately demands considerable expertise, often relying on veteran radiologists in PET imaging. Yet, the challenges posed by inadequate nuclear medicine facilities in resource-limited regions make it difficult for physicians to make an immediate and correct diagnosis based on medical imaging^10^.

The potential of artificial intelligence (AI) in PET imaging to automate diagnosis is attracting considerable interest and is becoming a research focus, as it could help solve the aforementioned problem of limited healthcare resources in areas with high diagnostic demand for medical imaging^11,12^. Deep learning (DL) utilizes multi-layered artificial neural networks for data analysis, whereas machine learning (ML) employs algorithms that enable computers to learn from data without being explicitly programmed. Conversely, transfer learning (TL) applies knowledge acquired from one task to improve performance on a related task. Integrating molecular medical images with AI algorithms, particularly ML and DL, has demonstrated potential in identifying PD patients^13^. DL algorithms utilize a variety of methods to achieve predictions and classifications from large, complex datasets. This has led to a number of groundbreaking innovative applications in medical imaging, where DL strategies have the potential to far outperform human experts. Researchers have attempted to improve diagnostic accuracy in a variety of ways, including expanding sample size and optimizing algorithms. Wu et al. demonstrated that a DL algorithm, when applied to ^18^F-FDG PET images, achieved a diagnostic accuracy of 98.6%^14^. In addition, an ML model was used to automatically discriminate between PD and normal control (NC) images, with a high accuracy of 71.2%^15^.

Although the number of research studies on AI-assisted PET imaging for the detection of PD has increased, a quantitative synthesis that comprehensively summarizes the available evidence is still lacking. Recent literature has also underscored the importance of modifying and adapting current research methodologies in line with the digital shift in healthcare^16^. This study therefore systematically reviews and meta-analyzes the published data on the diagnostic performance of AI algorithms-assisted PET scans for the detection of PD to provide a clear overview of the current situation, issues, and potential future directions of this tool in the digital era.

## Results

### Study selection and characteristics of eligible studies

**Fig. 1:**
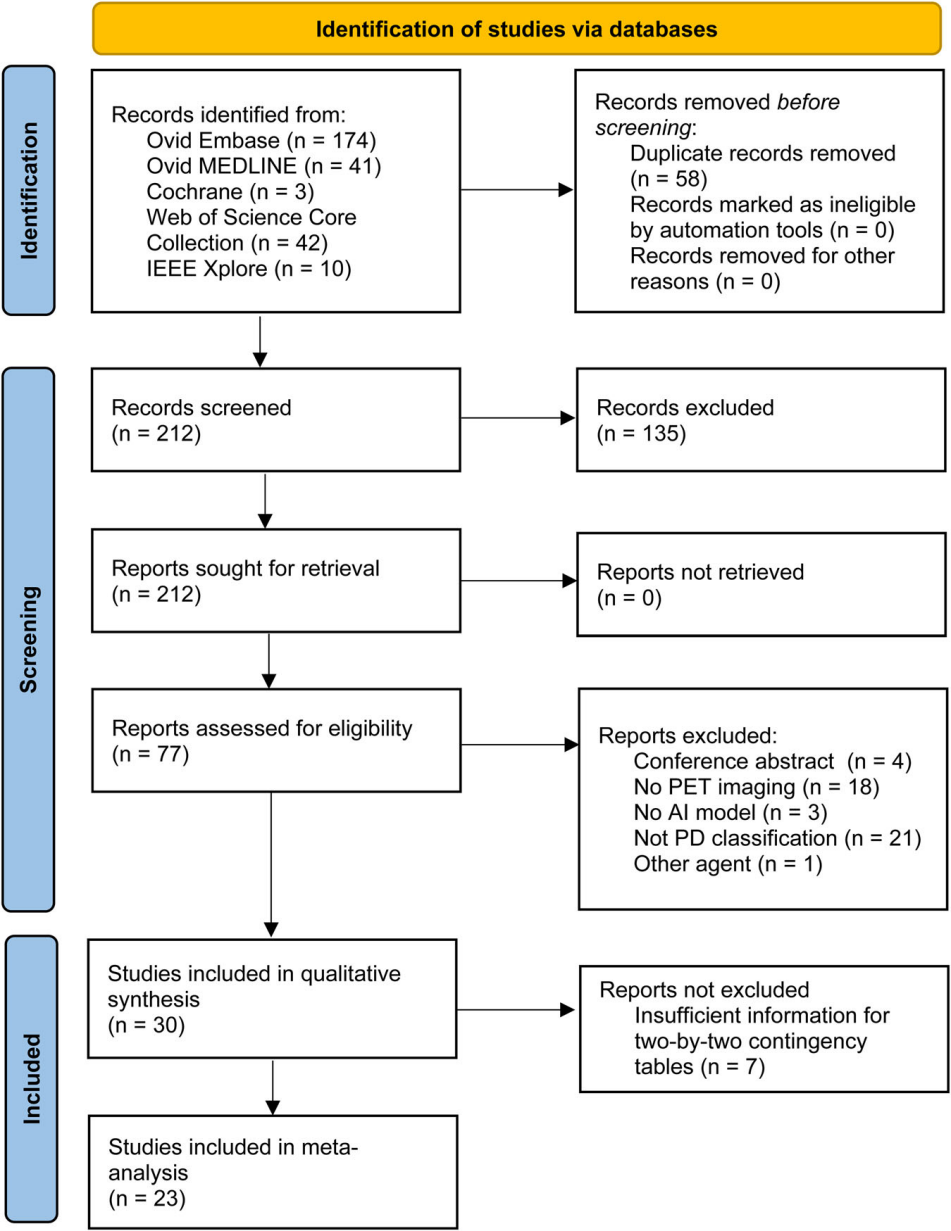
PRISMA flowchart of study selection. PRISMA (preferred reporting items for systematic reviews and meta-analyses) flow diagram of included studies.

A total of 270 records were found in the initial search, among which 58 were duplicates. Following the screening of titles and abstracts, 135 studies were excluded, leaving 77 articles for full-text eligibility assessment. Of these, 47 were further excluded, resulting in 30 studies that were included for the qualitative synthesis. However, seven of these studies were later excluded due to the insufficient information for constructing two-by-two contingency tables. Consequently, twenty-three articles contained sufficient data to meet the inclusion criteria for meta-analysis (Fig. 1)^14,15,17–37^.

The detailed characteristics of these included studies are shown in Table 1 and Supplementary Table 1. All but one study used prospective patient data (1/23), and the remaining study used retrospective data (22/23). One study used images from public databases. All studies recruited patients based on routine clinical diagnosis. In terms of imaging techniques, five studies utilized both PET and structural MRI to inform the AI model, while the remaining eighteen relied exclusively on PET imaging. Four studies used out-of-sample datasets to perform external validation. Twenty-one studies used a single imaging agent—twelve for glucose metabolism and nine for the dopaminergic system and two studies employed two types of imaging agents.

**Table 1:** Characteristics of all included studies (*n* = 23).

Author. year^[ref]^	Algorithm details							Data characteristics				
	Model	Number of patients	Training/validation (ratio)	Testing	Type of internal validation	External validation	ML/DL	Target condition	Imaging agent	Source of data	Prospective	Data range
Sun et al.^17^	LASSO; LR	119	84	35	Ten-fold cross-validation	No	ML	PD vs. AP	^11^C-CFT ^18^F-FDG	Department of Nuclear Medicine, Daping Hospital, Army Medical University, Chongqing, China	No	2015.1–2019.3
Wu et al.^14^	CNN	2228	945	330	Six-fold cross-validation	Yes	DL	PD vs. AP	^18^F-FDG	(a) Huashan Parkinsonian PET Imaging database; (b) University Hospital of Munich	No	2011.6–2019.4
Zhao et al.^18^	CNN	1017	737	280	Six-fold cross-validation	No	DL	PD vs. AP	^11^C-CFT	Huashan Parkinsonian PET Imaging database	No	NR
Xu et al.^19^	SVM	129	NR	NR	Leave-one-out cross-validation	No	ML	PD vs. AP	^11^C-CFT	PD Database and Samples Bank of Huashan Hospital, Fudan University, Shanghai, China	No	NR
Sun et al.^20^	SVM; LR	406	358	48	Five-fold cross-validation (100 times)	Yes	ML	PD vs. NC	^18^F-FDG	(a) PD Database and Samples Bank of Huashan Hospital, Fudan University, Shanghai, China; (b) Wuxi 904 Hospital, Jiangsu, China	No	NR
	CNN	406	358	48	Five-fold cross-validation (100 times)	Yes	DL	PD vs. NC	^18^F-FDG	(a) PD Database and Samples Bank of Huashan Hospital, Fudan University, Shanghai, China; (b) Wuxi 904 Hospital, Jiangsu, China	No	NR
Yoon et al.^21^	SVM; LR; XGBoost	127	NR	NR	NR	No	ML	PD vs. NC	^18^F-FP-CIT	Dong-A Medical Center	No	NR
Piccardo et al.^22^	CNN	98	68	30	Random split sample validation	No	DL	PD vs. NC	^18^F-DOPA	Department of Nuclear Medicine, Italy	No	2016.1–2018.1
Martins et al.^23^	SVM; LASSO	61	NR	NR	Ten-fold cross-validation (50 times)	No	ML	PD vs. NC PD vs. AP	^11^C-RAC	Institute of Nuclear Sciences Applied to Health, University of Coimbra	No	2013.4–2017.2
Hu et al.^24^	LASSO	90	63	27	Ten-fold cross-validation	No	ML	PD vs. AP	^18^F-FDG	Department of Nuclear Medicine, Union Hospital, Tongji Medical College, Wuhan, China	No	2017.12–2019.6
Dai et al.^25^	CNN	1350	NR	NR	Five-fold cross-validation	No	DL	PD vs. NC	^18^F-FDG	PPMI database	No	NR
Choi et al.^26^	CNN	527	456 (3:1)	71	Four-fold cross-validation	No	DL	PD vs. AP	^18^F-FP-CIT	Department of Nuclear Medicine, Daegu Catholic University Medical Center, Daegu Catholic University School of Medicine, Korea	No	2016–2019
Van et al.^27^	LVQ	214	NR	NR	Ten-fold cross-validation (10 times)	No	ML	PD vs. NC	^18^F-FDG	(a) Movement Disorder Unit of the Clinica Universidad de Navarra, the University Medical Center Groningen; (b) University of Genoa and IRCCS AOU San Martino-IST	No	NR
Rus et al.^28^	LR	99	NR	NR	Leave-one-out cross-validation	No	ML	PD vs. AP	^18^F-FDG	Department of Nuclear Medicine at UMC Ljubljana	No	2012.10–2015.8
Wu et al.^29^	SVM; RF	230	80%	20%	Five-fold cross-validation (500 times)	Yes	ML	PD vs. NC	^18^F-FDG	(a) PD Database and Samples Bank of Huashan Hospital, Fudan University, Shanghai, China; (b) Wuxi 904 Hospital, Jiangsu, China	No	NR
Shen et al.^30^	CNN	350	250 (4:1)	100	Random cross-validation	Yes	DL	PD vs. NC	^18^F-FDG	(a) Huashan Hospital, Fudan University, Shanghai, China; (b) 904 Hospital in Wuxi, China	No	(a) NR; (b) 2011–2015
Manzanera et al.^31^	CNN	310	270	40	Five-fold cross-validation	No	DL	PD vs. NC	^18^F-FDG	(a) University Medical Center Groningen, Netherlands; (b) University of Genoa, Italy; (c) Clinic University of Navarra, Spain	No	NR
	SVM		270	40	Five-fold cross-validation	No	ML	PD vs. NC	^18^F-FDG	(a) University Medical Center Groningen, Netherlands; (b) University of Genoa, Italy; (c) Clinic University of Navarra, Spain	No	NR
Glaab et al.^15^	SVM; RF	75	50%	50%	Leave-one-out cross-validation	No	ML	PD vs. NC	^18^F-FDG	University Hospital Cologne	Yes	NR
									^18^F-DOPA	University Hospital Cologne	No	NR
Segovia, F et al.^32^	SVM	87	NR	NR	Five-fold cross-validation	No	ML	PD vs. AP	^18^F-DMFP	University of Munich	No	NR
Segovia, F et al.^33^	SVM	87	NR	NR	Ten-fold cross-validation	No	ML	PD vs. AP	^18^F-DMFP	University of Munich	No	NR
Segovia, F et al.^34^	SVM	87	NR	NR	Leave-one-out cross-validation	No	ML	PD vs. AP	^18^F-DMFP	University of Munich	No	NR
Mudali et al.^35^	DT	79	No	NR	Leave-one-out cross-validation	No	ML	PD vs. NC	^18^F-FDG	NR	No	1998–2008
Garraux et al.^36^	RVM	120	No	NR	Bootstrapping cross-validation	No	ML	PD vs. AP	^18^F-FDG	Cyclotron Research Centre, University of Liège, University Hospital Center of Liège	No	1993–2009
Tang et al.^37^	LR	167	No	NR	Leave-one-out cross-validation	No	ML	PD vs. AP	^18^F-FDG	Functional neuroimaging laboratory at The Feinstein Institute for Medical Research, New York, USA	No	1998.1–2006.12

*NC* normal control, *PD* Parkinson’s disease, *AP* atypical parkinsonism, *DL* deep learning, *ML* machine learning, *CNN* convolutional neural network, *LR* logistic regression, *RVM* relevance vector machine, *LASSO* least absolute shrinkage and selection operator method, *LVQ* learning vector quantization, *DT* decision tree, *SVM* support vector machine, *RF* Random Forest, *XGBoost* tree gradient boosting, *PPMI* Parkinson’s Progression Markers Initiative, ^*18*^*F-DMFP* ^18^F-Desmethoxyfallypride, ^*18*^*F-FDG* ^18^F-fluorodeoxyglucose, ^*18*^*F FP-CIT* ^18^F N-(3-fluoropropyl)-2β-carboxymethoxy-3β-(4-iodophenyl) nortropane, ^*11*^*C-CFT* ^11^C-2β-carbomethoxy-3β-(4-fluorophenyl) tropane, ^*18*^*F-DOPA* ^18^F-Fluoro-dihydroxyphenylalanine, ^*11*^*C-RAC* ^11^C-raclopride, *NR* not reported.

In addition, the distribution of studies concerning the classification of PD in the present study is as follows: 11 studies on the classification of PD from NC and 13 studies on the classification of PD from AP (more details see Supplementary Table 2). Supplementary Tables 3−4 provide a detailed enumeration of the various categories using different PET imaging tracers. Tables 2 and 3 summaries the estimate of the pooled performance of AI-assisted PET imaging for the diagnosis of PD. Forest plots can be found in the Supplementary Figs. 1−18.

**Table 2:** Summary estimates and meta-regression of pooled performance of AI-assisted PET imaging in the diagnosing PD from NC.

Parameter	No. of tables	AUC (95% CI)	Sensitivity (%)		*p* value^a^	Specificity (%)		*p* value^a^	LR+ (95% CI)	LR- (95% CI)
			SE (95% CI)	*I*^*2*^ (95% CI)		SP (95% CI)	*I*^*2*^ (95% CI)			
Presynaptic DA										
Overall	21	0.96 (0.94–0.97)	91.47 (87.01–94.50)	79.85 (71.78–87.91)		88.23 (82.34–92.34)	70.44 (57.34–83.55)		7.77 (5.06–11.95)	0.10 (0.06–0.15)
Postsynaptic DA										
Overall	3	-	-	-		-	-		-	-
^18^F-FDG										
Overall	116	0.90 (0.87–0.93)	83.66 (81.42–85.68)	82.20 (79.30–85.09)		83.81 (80.69–86.51)	90.37 (89.06–91.68)		5.17 (4.30–6.20)	0.19 (0.17–0.22)
Highest performance	8	0.95 (0.93–0.97)	91.98 (83.36–96.33)	91.04 (86.28–95.80)		84.02 (57.32–95.31)	96.78 (95.52–98.04)		5.76 (1.82–18.26)	0.10 (0.04–0.22)
*Algorithm*					<0.001			<0.001		
DL	53	0.93 (0.90–0.95)	87.84 (85.37–89.94)	79.03 (73.73–84.33)		84.69 (81.06–87.82)	90.43 (88.50–92.36)		5.74 (4.57–7.21)	0.14 (0.12–0.18)
ML	63	0.87 (0.83–0.89)	79.44 (76.06–82.46)	75.65 (69.78–81.53)		83.05 (77.45–87.49)	90.25 (88.44–92.06)		4.69 (3.5–6.27)	0.25 (0.21–0.29)
*Sample size*					<0.001			<0.001		
≥100	46	0.94 (0.92–0.96)	87.18 (84.72–89.29)	86.89 (83.77–90.00)		88.91 (86.25–91.10)	93.15 (91.81–94.49)		7.86 (6.23–9.91)	0.14 (0.12–0.17)
<100	70	0.86 (0.82–0.88)	79.58 (75.97–82.78)	69.42 (61.97–76.86)		78.87 (72.85–83.36)	81.86 (78.04–85.68)		3.71 (0.94–4.69)	0.26 (0.22–0.30)

*NC* normal control, *PD* Parkinson’s disease, *DL* deep learning, *ML* machine learning, ^*18*^*F-FDG* ^18^F-fluorodeoxyglucose, *DA* dopamine, *LR+* Positive likelihood ratio, *LR−* negative likelihood ratio, *SE* sensitivity, *SP* specificity, *AUC* under the curve.

^a^*p* value for heterogeneity between subgroups with meta-regression analysis.

**Table 3:** Summary estimates and meta-regression of pooled performance of AI-assisted PET imaging in the diagnosing PD from AP.

Parameter	No. of tables	AUC (95% CI)	Sensitivity (%)		*p* value^a^	Specificity (%)		*p* value^a^	LR+ (95% CI)	LR- (95% CI)
			SE (95% CI)	*I*^*2*^ (95% CI)		SP (95% CI)	*I*^*2*^ (95% CI)			
Presynaptic DA										
Overall	13	0.93 (0.91–0.95)	89.54 (87.11–91.56)	44.46 (8.46–80.47)		89.07 (81.87–93.63)	79.51 (68.95–90.07)		8.19 (4.79–14.02)	0.12 (0.09–0.15)
Highest performance	4	0.95 (0.93–0.97)	91.75 (82.94–96.22)	80.98 (62.68–99.28)		91.06 (64.09–98.31)	93.83 (89.41–98.25)		10.26 (2.01–52.29)	0.09 (0.04–0.21)
*Algorithm*										
DL	10	0.96 (0.94–0.97)	90.78 (88.52–92.63)	6.41 (0.00–97.89)		91.19 (88.21–93.48)	38.66 (0.00–84.07)		10.31 (7.69–13.827)	0.10 (0.08–0.13)
ML	3	-	-	-		-	-		-	-
Postsynaptic DA										
Overall	15	0.79 (0.75–0.82)	74.43 (68.84–79.33)	44.99 (11.74–78.23)		71.26 (66.62–75.49)	23.76 (0.00–70.68)		2.59 (2.25–2.98)	0.36 (0.30–0.43)
Highest performance	4	0.81 (0.77–0.84)	84.05 (66.13–93.43)	63.10 (22.96–100.00)		71.62 (61.22–80.13)	24.47 (0.00–100.00)		2.96 (2.22–3.94)	0.22 (0.10–0.48)
^18^F-FDG										
Overall	44	0.97 (0.96–0.99)	92.79 (90.66–94.47)	74.27 (66.81–81.74)		92.94 (90.14–94.99)	73.11 (65.21–81.01)		13.14 (9.35–18.47)	0.78 (0.06–0.10)
Highest performance	6	0.97 (0.95–0.98)	91.63 (85.57–95.28)	49.28 (2.43–96.13)		95.36 (84.64–98.71)	63.06 (30.33–95.79)		19.76 (5.57–70.11)	0.09 (0.05–0.16)
*Algorithm*					<0.001			<0.001		
DL	19	0.99 (0.97–0.99)	96.17 (94.73–97.22)	61.77 (42.74–80.79)		94.63 (92.53–96.16)	62.44 (43.83–81.05)		17.90 (12.92–24.80)	0.04 (0.03–0.06)
ML	26	0.93 (0.91–0.95)	87.72 (84.52–90.34)	45.80 (20.83–70.78)		91.14 (84.89–94.96)	69.97 (57.98–81.96)		9.90 (5.70–17.19)	0.13 (0.11–0.17)
*Sample size*					<0.001			<0.001		
≥100	21	0.99 (0.97–0.99)	95.61 (93.88–96.86)	73.16 (61.56–84.77)		95.13 (93.01–96.63)	65.86 (50.16–81.56)		19.64 (13.75–28.05)	0.05 (0.03–0.06)
<100	24	0.94 (0.91–0.96)	87.96 (84.20–90.93)	53.30 (31.17–75.43)		89.03 (82.30–93.40)	61.13 (43.49–78.76)		8.01 (4.88–13.16)	0.14 (0.10– 0.18)

*PD* Parkinson’s disease, *AP* atypical parkinsonism, *DL* deep learning, *ML* machine learning, ^*18*^*F-FDG* ^18^F-fluorodeoxyglucose, *DA* dopamine, *LR+* Positive likelihood ratio, *LR−* negative likelihood ratio, *SE* sensitivity, *SP* specificity, *AUC* under the curve.

^a^*p* value for heterogeneity between subgroups with meta-regression analysis.

### Pooled performance of AI algorithms for classifying PD from NC

**Fig. 2:**
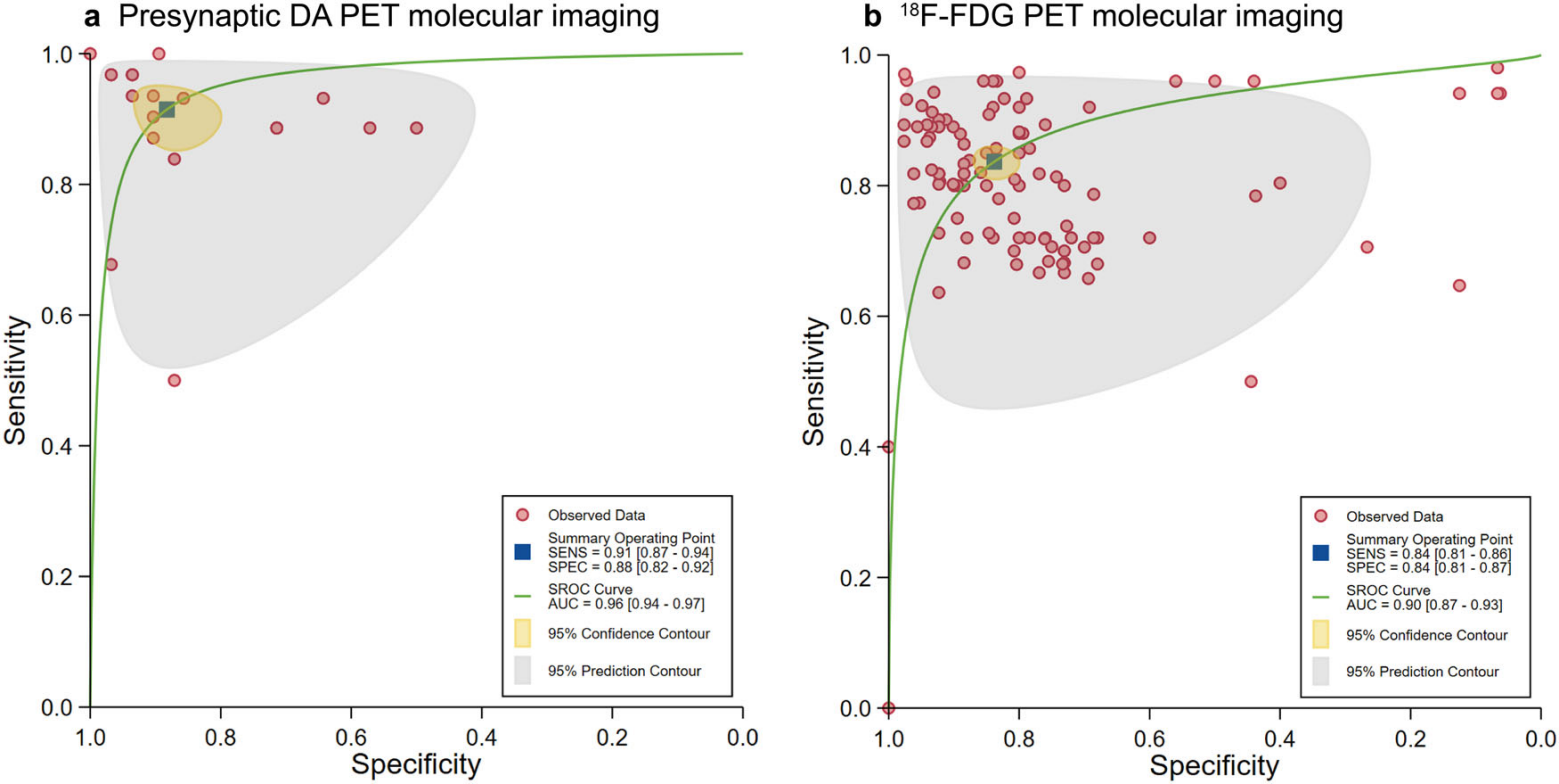
Hierarchical summary receiver operating characteristic (SROC) curves of studies included in the meta-analysis to classify Parkinson’s disease from normal control (11 studies). The 95% prediction region is a visual representation of between-study heterogeneity. Presynaptic DA PET molecular imaging by using ML algorithms (21 contingency tables from three studies) (**a**), and ^18^F-FDG PET molecular imaging by using AI algorithms (116 contingency tables from eight studies) (**b**).

A total of 21 contingency tables from three studies on presynaptic DA PET imaging, all utilizing the ML algorithm. The pooled sensitivity (SE), specificity (SP), and area under the curve (AUC) for this group were 91.47% (95% CI: 87.01–94.50), 88.23% (95% CI: 82.34–92.34), and 0.96 (95% CI: 0.94–0.97) respectively (Fig. 2a).

Eight studies involving ^18^F-FDG PET imaging provided sufficient data for constructing contingency tables and determining diagnostic performance metrics. For these studies, the pooled SE, SP, and AUC were 83.66% (95% CI: 81.42–85.68), 83.81% (95% CI: 80.69–86.51), and 0.90 (95% CI: 0.87–0.93) respectively (Fig. 2b). When the contingency table with the highest performance was selected, yielded a pooled SE of 91.98% (95%CI: 83.36–96.33), SP of 84.02% (95%CI: 57.32–95.31), and AUC of 0.95 (95% CI: 0.93–0.97) (Table 2). For ^18^F-FDG PET imaging, two distinct subgroup meta-analyses were performed as follows:

Regarding AI algorithms, 53 contingency tables from four studies utilized the DL algorithm, while 63 tables from six studies employed the ML algorithm. The hierarchical summary receiver operating characteristic (SROC) curves for these algorithms are depicted in Supplementary Fig. 19. The pooled SE for DL was 87.84% (95% CI: 85.37–89.94), and for ML was 79.44% (95% CI: 76.06–82.46); pooled SP was 84.69% (95% CI: 81.06–87.82) for DL and 83.05% (95% CI: 77.45–87.49) for ML. The AUC was 0.93 (95% CI: 0.90–0.95) for DL and 0.87 (95% CI: 0.83–0.89) for ML. The diagnostic accuracy using various ML algorithms are further detailed in Supplementary Table 5 and Supplementary Figs. 20−21.

With respect to sample sizes, 46 contingency tables were derived from samples exceeding 100, while 70 tables involved smaller samples. The hierarchical SROC curves for these sample size subgroups are shown in Supplementary Fig. 22. The pooled SE for samples larger than 100 was 87.18% (95% CI: 84.72–89.29), and for samples smaller than 100 was 79.58% (95% CI: 75.97–82.78); pooled SP was 88.91% (95% CI: 86.25–91.10) for larger samples and 78.87% (95% CI: 72.85–83.36) for smaller samples. The AUC was 0.94 (95% CI: 0.92–0.96) for the larger sample size group and 0.86 (95% CI: 0.82–0.88) for the smaller one.

### Pooled performance of AI algorithms for classifying PD from AP

**Fig. 3:**
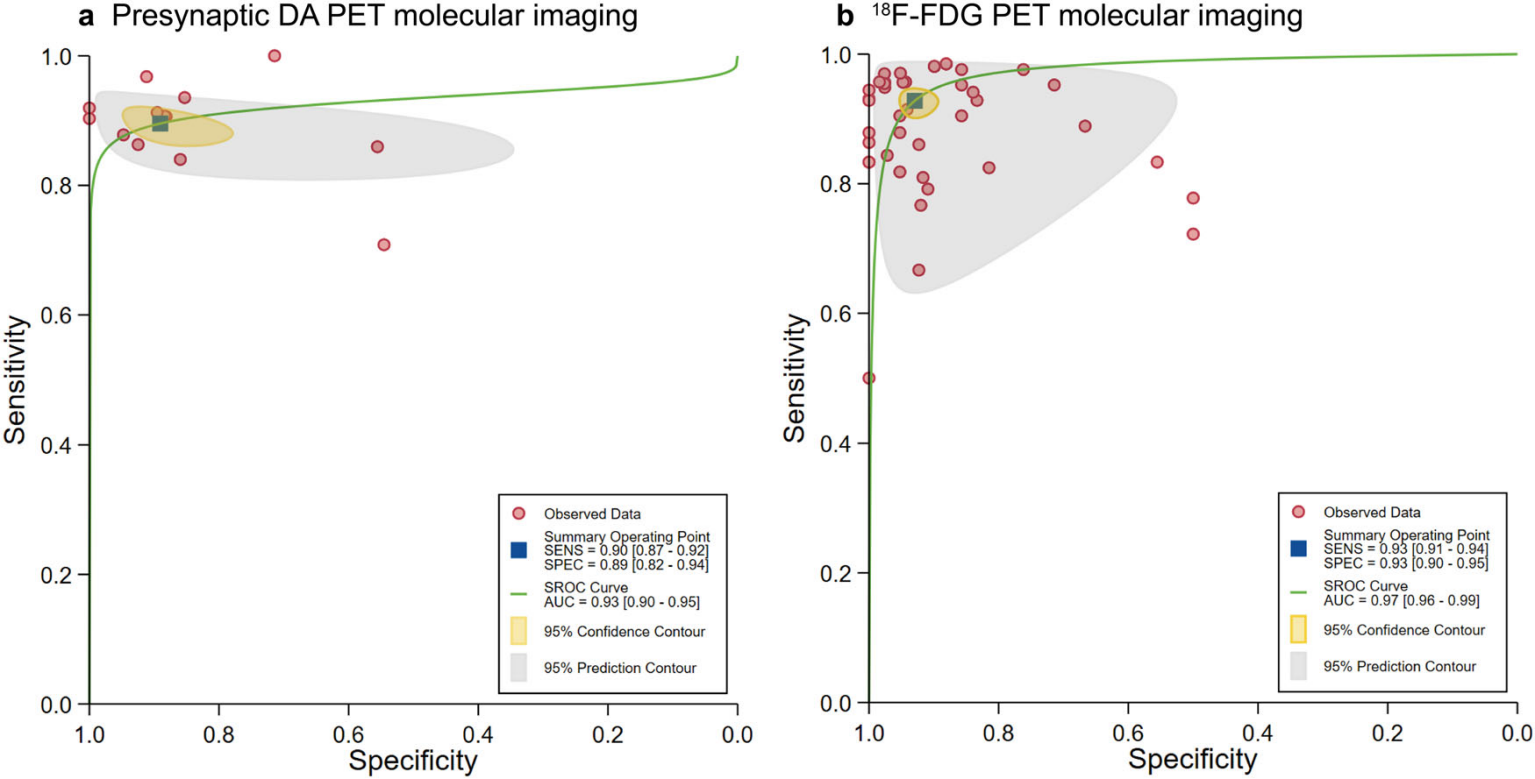
Hierarchical summary receiver operating characteristic (SROC) curves of studies included in the meta-analysis to classify Parkinson’s disease from atypical parkinsonism by using AI algorithms (13 studies). The 95% prediction region is a visual representation of between-study heterogeneity. Presynaptic DA PET molecular imaging (13 contingency tables from four studies) (**a**), and ^18^F-FDG PET molecular imaging (45 contingency tables from six studies) (**b**).

A total of 13 contingency tables from presynaptic DA PET imaging analyses were included. The pooled SE, SP, and AUC were 89.54% (95% CI: 87.11–91.56), 89.07% (95% CI: 81.87–93.63), and 0.93 (95% CI: 0.91–0.95), respectively (Fig. 3a). Selecting the contingency table with the highest performance yielded a pooled SE of 91.75% (95% CI: 82.94–96.22), SP of 91.06% (95% CI: 64.09–98.31), and AUC of 0.95 (95% CI: 0.93–0.97). Among these, 10 out of 13 contingency tables utilized DL algorithms, with a pooled SE, SP, and AUC of 90.78% (95% CI: 88.52–92.63), 91.19% (95% CI: 88.21–93.48), and 0.96 (95% CI: 0.94–0.97), respectively.

For postsynaptic DA PET imaging, 15 contingency tables were included, all employing ML algorithms. The pooled results within this group were a SE of 74.43% (95% CI: 68.84–79.33), SP of 71.26% (95% CI: 66.62–75.49), and AUC of 0.79 (95% CI: 0.75–0.82). When the contingency table with the highest performance was selected, the pooled SE, SP and AUC were 84.05% (95% CI: 66.13–93.43), 71.62% (95% CI: 61.22–80.13), and 0.81 (95% CI: 0.77–0.84), respectively.

Six studies on ^18^F-FDG PET imaging provided sufficient data for computing contingency tables and testing performance metrics. The pooled estimates for this group were 92.79% SE (95% CI: 90.66–94.47), 92.94% SP (95% CI: 90.14–94.99), and an AUC of 0.97 (95% CI: 0.96–0.99) (Fig. 3b). The contingency tables with highest performance showed an SE of 91.63% (95% CI: 85.57–95.28), SP of 95.36% (95% CI: 84.64–98.71), and AUC of 0.97 (95% CI: 0.95–0.98). Two separate subgroup meta-analyses for ^18^F-FDG PET imaging are presented as follows.

Regarding AI algorithms, 19 contingency tables applied the DL algorithm, while 26 applied the ML algorithm. Hierarchical SROC curves for these algorithms are shown in Supplementary Fig. 23. The pooled SE was 96.17% (95% CI: 94.73–97.22) for DL and 87.72% (95% CI: 84.52–90.34) for ML, with corresponding SP of 94.63% (95% CI: 92.53–96.16) for DL and 91.14% (95% CI: 84.89–94.96) for ML. The AUC for DL was 0.99 (95% CI: 0.97–0.99) and for ML was 0.93 (95% CI: 0.91–0.95). The diagnostic accuracy using various ML algorithms are further detailed in Supplementary Table 6.

In terms of sample sizes, 21 contingency tables had more than 100 samples, while 24 had fewer than 100. The hierarchical SROC curves for these sample size subgroups are illustrated in Supplementary Fig. 24. The pooled SE for groups with over 100 samples was 95.61% (95% CI: 93.88–96.86) and for those with fewer than 100 samples was 87.96% (95% CI: 84.20–90.93). Pooled SP for the larger sample group was 95.13% (95% CI: 93.01–96.63) and for those with fewer than 100 samples was 89.03% (95% CI: 82.30–93.40). The AUC was 0.99 (95% CI: 0.97–0.99) for larger samples and 0.94 (95% CI: 0.91–0.96) for smaller samples.

### Heterogeneity analysis

The meta-analysis of the included studies suggests a potential benefit of AI algorithms in assisting the diagnosis of PD using PET molecular imaging; however, the heterogeneity observed in some subgroups suggests that cautious interpretation and further validation are required.

Moderate to high heterogeneity was observed in distinguishing PD from NC using presynaptic DA PET imaging, with an *I*^*2*^ of 79.85% (95% CI: 71.78–87.91) for SE and an *I*^*2*^ of 70.44% (95% CI: 57.34–83.55) for SP. In addition, heterogeneity was lower for PD classification of AP, with an *I*^*2*^ of 44.46% (95% CI: 8.46–80.47) for SE and an *I*^*2*^ of 79.51% (95% CI: 68.95–90.07) for SP. In contrast, when DL algorithms were utilized in the subgroup analysis, low heterogeneity was observed, with an *I*^*2*^ for SE of 6.41% (95% CI: 0.00–97.89) and an *I*^*2*^ for SP of 38.66% (95% CI: 0.00–84.07) for the classification of PD and AP.

In postsynaptic DA PET imaging, low heterogeneity was observed in distinguishing PD from AP, as indicated by an *I*^*2*^ of 44.99% (95% CI: 11.74–78.23) for SE and 23.76% (95% CI: 0.00–70.68) for SP.

Moderate heterogeneity in the classification of PD and AP was observed with ^18^F-FDG PET imaging, with an *I*^*2*^ for SE and SP of 74.27% (95% CI: 66.81–81.74) and 73.11% (95% CI: 65.21–81.01), respectively. However, substantial heterogeneity was found in the classification of PD and NC, with SE at an *I*^*2*^ of 82.20% (95% CI: 79.30–85.09) and SP at an *I*^*2*^ of 90.37% (95% CI: 89.06–91.68). Subgroup analyses were conducted to explore the sources of this pronounced heterogeneity. Although *I*^*2*^ values remained high in most subgroups with ^18^F-FDG PET imaging, heterogeneity was reduced to an acceptable level in some subgroups.

Detailed results of all subgroups and meta-regression analyses examining the potential source of heterogeneity between studies are shown in Tables 2−3 and Supplementary Tables 7−10. The results revealing statistically significant differences in covariates. Publication bias of groups and subgroups resulting from visual inspection of funnel plots are shown in Supplementary Figs. 25−33.

### Quality assessment

The quality of the included studies was determined by the quality assessment of diagnostic accuracy studies-AI (QUADAS-AI). Detailed assessment results are shown in a diagram in Supplementary Fig. 34 and Supplementary Table 11. More than half of the studies had high or unclear risk of bias for patient selection (*n* = 12) and the index test (*n* = 19) because these studies had not clarified the description of included patients with detailed information about previous tests, presentation, setting, intended use of the index test, and lack of adequate external validation.

## Discussion

The role of PET molecular imaging in PD diagnosis has gained importance in recent years, leading to an increase in studies investigating AI as a potential diagnostic tool. Thus, we attempted to ascertain which is the most accurate and reliable AI detection technology for PD diagnosis within PET molecular imaging currently available. By strictly adhering to diagnostic review guidelines, we were able to maintain the integrity of the study. Our findings indicate that AI algorithms demonstrate high diagnostic accuracy in differentiating PD from NC and AP. Specifically, the pooled AUC for presynaptic DA was 0.96 (95% CI: 0.94–0.97) and 0.90 (95% CI: 0.87–0.93) for ^18^F-FDG in classifying PD from NC. In distinguishing PD from AP, the pooled AUCs were 0.93 (95% CI: 0.91–0.95) for presynaptic DA, 0.79 (95% CI: 0.75–0.82) for postsynaptic DA, and 0.97 (95% CI: 0.96–0.99) for ^18^F-FDG. While these results highlight the potential of AI in detecting PD, our analysis also identified significant methodological limitations, which may limit its practical application.

Standardization of data attenuates confounding factors and improves CNN learning of meaningful patterns and features from neuroimaging data. Previous studies suggested that preprocessing is critical for the reliability and validity of CNN-based neuroimaging studies in PD^38^, improving the quality of imaging data and the accuracy of the AI model^39^. Most studies in the present study have considered this step. Nevertheless, there are some potential drawbacks, such as time consumption, inaccuracy, etc., which need to be streamlined and simplified as AI technology advances.

The lack of transparency of DL models can be challenging for clinicians to understand diagnostic strategies, affecting their confidence in predictions^40^. Emerging explainable artificial intelligence (XAI), such as layer-wise relevance propagation (LRP), can improve the interpretability of models by highlighting input features and providing explanations about the model^41,42^. Despite the infancy of PD, understanding its principles and applications will accelerate the utility of this practice. In the clinical context, AI can help clarify the factors the model considers in predicting disease progression. DL can analyze complex physiological data to detect disease onset and progression and potentially discover new biomarkers or risk factors. However, the ability of DL, to uncover correlations and associations should not be confused with the direct discovery of causality. Therefore, any patterns discovered by AI will require further research to confirm and understand the underlying mechanisms.

The studies reviewed showed considerable heterogeneity due to differences in AI methods, sample sizes, and imaging modalities^43^. The results of these subgroup meta-analyses of the variety of ML approaches (such as SVM, RF, LR, XGBoost, etc.), demonstrate the variability in diagnostic performance across the different ML methods, which could introduce heterogeneity in our meta-analysis. In addition, none of the studies performed sample size calculations, a glaring deficiency in reports of AI models^10,44^. The performance of AI models depends on large datasets; small datasets could compromise their accuracy and generalizability. In the current study, the diagnostic accuracy of the small-sample study was significantly lower than the results of the large-sample study. In addition, current research typically relies on databases that are not open and rarely curated, which significantly hinders AI model learning. We advocate for larger, more diverse image databases for PD patients and an international consensus on the use of PET in clinical contexts^45^. Regarding the imaging modalities used, five studies utilized both PET and structural MRI to build the AI model, while the remaining eighteen relied solely on PET imaging. The heterogeneity of these studies, coupled with the ‘black box’ nature of their methodology represents a notable limitation of this research. Consequently, we emphasize the compelling need for standardization of AI methods and reporting practices. Such standardization is critical to improving the consistency and transparency of future research in this area.

External validation significantly impacts the risk of bias and the generalizability of AI diagnostic studies. It is essential that only externally validated models be employed in clinical practice^46^. Only four of the studies included offered external validation. Consequently, performance might decline if the algorithm is applied in routine clinical practice, where all patients with suspected PD are screened. Approximately half of the studies reviewed were at high risk for bias, resulting in potentially inflated performance estimates. Future research design should include rigorous external validation, with multicenter studies playing a vital role. Most studies are based on retrospective hospital data; prospective studies yield more robust evidence, bridging the gap between anticipated and actual effects.

Terminological discrepancies in AI research make it difficult to distinguish independent datasets as found in the literature. To address this issue, we propose to divide datasets into training, tuning, and validation sets for model training, parameter optimization, and performance evaluation. Therefore, datasets used for in-sample validation should be referred to as internal validation sets, while out-of-sample validation should be referred to as external validation sets^46^. The consistency of these parameters will improve the quality of the study.

AI’s potential to rapidly analyze medical images and integrate data from multiple sources can improve the diagnostic process and be particularly beneficial in emergencies. In addition, AI can facilitate junior physicians training by providing immediate diagnostic feedback^47^. A collaborative human-AI model could optimize diagnostic accuracy by integrating the unique strengths of both components and potentially incorporating nonimage-based patient data such as demographic information and history of motor impairment^48–50^. These AI methods, with their potential for quality assurance and personalized, predictive medicine, represent promising models for improving healthcare. However, given the variability of diseases and the urgent need for mechanism research, a standardized molecular AI application for imaging remains a distant goal.

A recent study reported high diagnostic accuracy using standard reporting protocols with ^18^F-FDG PET, achieving an overall accuracy of 74% in distinguishing PD from AP^51^. Meta-analytic evidence also suggests that ^18^F-FDG PET, when used with metabolic pattern analysis, discriminates PD from NC with a pooled SE of 0.88 (95% CI: 0.82–0.92), a pooled SP of 0.90 (95% CI: 0.85–0.94), and an AUC of 0.95 (95% CI: 0.93–0.97). It also separates PD from AP with comparable efficacy, showing a pooled SE of 0.88 (95% CI: 0.84–0.91) and a pooled SP of 0.93 (95% CI: 0.89–0.96) and an AUC of 0.95 (95% CI: 0.93–0.97)^52^. This indicates that the metabolic pattern appears to have higher accuracy than AI-assisted ^18^F-FDG. Previous studies have also shown the consistency of different semiquantitative presynaptic dopaminergic PET imaging in PD diagnosis^53^. Therefore, we divided the dopaminergic radioligands into presynaptic and postsynaptic DA to analyze the diagnostic performance for PD. In the present study, AI-assisted presynaptic DA PET appears to have similar performance to other results from the meta-analysis, which showed an AUC of 0.95 (95% CI: 0.92–0.97) for distinguishing PD from NC using traditional tracer uptake of presynaptic dopaminergic neuroimaging^54^. This ability to extract complex data features from medical images that are unobservable or unquantifiable to the human eye increases diagnostic potential and contributes to disease progression modeling. AI-assisted presynaptic DA appears to have better performance in discriminating PD from NC, and AI-assisted ^18^F-FDG appears to have better performance in discriminating PD from AP, supporting the diagnostic pathway (two-step) of PET imaging in clinical practice for PD^55^. Normal dopaminergic imaging was included as an absolute exclusion criterion and the clinical utility of ^18^F-FDG PET in distinguishing PD from AP.

This study has several limitations that warrant consideration. Firstly, we focused on English-language articles, potentially overlooking valuable findings from non-English studies. Secondly, for studies with insufficient information, we did not contact the authors to provide the required data. Future research should aim to validate the performance of AI in real-world conditions. The majority of the included articles relied on clinical diagnostic criteria without pathological verification when diagnosing PD, introducing another potential limitation in our findings. Furthermore, the MSA, PSP, and other subtypes were included in different proportions in the group of AP patients. Due to the limited literature, this study did not investigate where the heterogeneity of differences between studies originates from, and further research is needed to investigate the diagnostic accuracy of AI algorithms in more homogeneous patient groups. Given the limited availability of PET data, ML methods used for meta-analysis in the literature may be prone to overfitting problems, and the generalizability of data from different sites is not addressed in this study, which is also our future research direction.

This research highlights the considerable potential of AI algorithms in detecting PD using PET molecular imaging and points to a promising future in nuclear medicine^50^. Although the challenges such as false positive and negative risks, data privacy and security concerns, and regulatory approval requirements, AI is an important adjunct to assist physicians in diagnosis. We also highlight the need for improved research design in PD AI-based diagnostic systems.

## Methods

The systematic review and meta-analysis were performed according to the standard PRISMA (preferred reporting items for systematic reviews and meta-analyses)^56^. The study was registered in the PROSPERO (CRD42022367782).

### Search strategy and eligibility criteria

The Ovid MEDLINE, Ovid Embase, Web of Science Core Collection, Cochrane, and IEEE Xplore databases were systematically searched for studies that developed an AI algorithm in PET imaging for diagnostic performance from PD and were published by August 17, 2023. Only English-language articles were included. Supplementary Methods summarizes the search strategy used in each database. Eligible studies that reported AI-assisted PET imaging for the diagnosis of PD with diagnostic outcomes such as SE and SP were then used to calculate the 2 × 2 contingency tables. The inclusion/exclusion criteria of literature were listed in Supplementary Methods.

### Data analysis

The characteristics and diagnostic performance were extracted independently by two reviewers using a standardized data extraction sheet. Discrepancies were resolved by discussion, or a third reviewer was consulted. Information was collected on the data set, including participant demographics: inclusion criteria, exclusion criteria, total sample, reference standard; data characteristics: imaging agent, poor image quality information, data source; algorithm details: design, algorithm model, type of validation; and diagnostic accuracy data.

Binary diagnostic accuracy data were extracted and contingency tables were constructed at the reported thresholds. Diagnostic accuracy data, including SE, SP, AUC, true-positive (TP), false-positive (FP), true-negative (TN), and false-negative (FN) for the AI model, were extracted directly into contingency tables and used to calculate SE and SP. If a study provided multiple contingency tables for the same or different AI algorithms, the contingency tables for different AI algorithms were used independently. The contingency tables for the included studies are summarized in Supplementary Tables 12−13. An additional analysis of the included studies was performed to determine the optimal performance of an AI model. The contingency table with the highest performance from each study was selected, where the highest performing was defined either by the AUC or, if the AUC was not available, by the positive prediction (total number of true positives and true negatives).

The risk of bias and applicability of all selected studies were assessed by using the QUADAS-AI^57^ criteria. It provides researchers with a specific framework for assessing the risk of bias and applicability when conducting reviews that evaluate the accuracy of AI-assisted diagnostic tests. In addition, an applicability analysis was also conducted. The list of all questions used to assess signaling, risk of bias, and applicability can be found in Supplementary Table 14. All of the studies were reviewed and analyzed by at least two separate authors. When disagreements occurred, they were resolved either by consensus or by a third reviewer. The methodological quality of the included studies was evaluated using RevMan software (Version 5.4).

We estimated the diagnostic performance of AI algorithms using a meta-analysis of studies with contingency tables. The random-effects model was conducted because of the assumed differences between studies. We intended to perform a meta-analysis if at least five contingency tables were eligible for inclusion, which is recommended for random-effects meta-analysis^58^. We used the contingency tables to construct hierarchical SROC curves, forest plots, and to calculate pooled sensitivities and specificities, anticipating a high level of heterogeneity^59^. The combined curve was plotted with the corresponding 95% confidence region, and 95% prediction region around the averaged estimates of SE, SP, and AUC in the SROC figures. The risk of publication bias was assessed using the funnel plot and regression test. Heterogeneity was assessed using the *I*^*2*^ statistic (25–49% was considered to be low heterogeneity, 50–74% was moderate and >75% was high heterogeneity). The calculations were performed by using STATA statistical software (version 17.0) (Midas and Metandi modules; StataCorp). Statistical significance was indicated at a *P* value of 0.05.

Considering the difference of the control group in clinical utility, the included studies were first divided into the classification PD from the NC group and the classification PD from the AP group. The diagnostic performance of the different tracers (glucose metabolism, pre- and postsynaptic DA) was evaluated separately, as the functional and regional brain uptakes are varied in the radioligands. Subsequently, in order to identify the source/sources of the extreme heterogeneity, the subgroup analysis was conducted based on: (1) AI algorithms (ML or DL); (2) the sample size of the AI algorithms (≥100 or <100). A meta-regression analysis was conducted to investigate the sources of heterogeneity among studies, taking into account the type of AI algorithms and sample size as covariates.

### Reporting summary

Further information on research design is available in the Nature Research Reporting Summary linked to this article.

### Supplementary information

Reporting Summary

Supplementary materials

## Data Availability

The authors declare that all the data included in this study are available within the paper and its Supplementary Information files.

## References

[1] Lang AE, Lozano AM (1998). Parkinson’s disease. N. Engl. J. Med..

[2] Salat D, Noyce AJ, Schrag A, Tolosa E (2016). Challenges of modifying disease progression in prediagnostic Parkinson’s disease. Lancet Neurol..

[3] Tolosa E, Garrido A, Scholz SW, Poewe W (2021). Challenges in the diagnosis of Parkinson’s disease. Lancet Neurol..

[4] Hughes AJ, Daniel SE, Ben-Shlomo Y, Lees AJ (2002). The accuracy of diagnosis of Parkinsonian syndromes in a specialist movement disorder service. Brain.

[5] Tarsy D, Apetauerova D, Ryan P, Norregaard T (2003). Adverse effects of subthalamic nucleus DBS in a patient with multiple system atrophy. Neurology.

[6] Armstrong MJ, Okun MS (2020). Diagnosis and treatment of Parkinson’s disease: a review. JAMA.

[7] Liu FT (2023). Dopaminergic dysfunction and glucose metabolism characteristics in parkin-induced early-onset Parkinson’s disease compared to genetically undetermined early-onset Parkinson’s disease. Phenomics.

[8] Wu L (2018). Clinical characteristics of cognitive impairment in patients with Parkinson’s disease and its related pattern in (18) F-FDG PET imaging. Hum. Brain Mapp..

[9] Yang YJ (2019). Preserved caudate function in young-onset patients with Parkinson’s disease: a dual-tracer PET imaging study. Ther. Adv. Neurol. Disord..

[10] Varoquaux G, Cheplygina V (2022). Machine learning for medical imaging: methodological failures and recommendations for the future. npj Digit. Med..

[11] Santosh, K., Antani, S., Guru, D. & Dey, N. *Medical Imaging: Artificial Intelligence, Image Recognition, and Machine Learning Techniques* 1st edn (CRC Press, 2019).

[12] Esteva A (2019). A guide to deep learning in healthcare. Nat. Med..

[13] Boutet A (2021). Predicting optimal deep brain stimulation parameters for Parkinson’s disease using functional MRI and machine learning. Nat. Commun..

[14] Wu P (2022). Differential diagnosis of parkinsonism based on deep metabolic imaging indices. J. Nucl. Med..

[15] Glaab E (2019). Integrative analysis of blood metabolomics and PET brain neuroimaging data for Parkinson’s disease. Neurobiol. Dis..

[16] Guo C (2020). Challenges for the evaluation of digital health solutions-a call for innovative evidence generation approaches. npj Digit. Med..

[17] Sun, J. et al. Identification of Parkinson’s disease and multiple system atrophy using multimodal PET/MRI radiomics. *Eur. Radiol.*10.1007/s00330-023-10003-9 (2023). 10.1007/s00330-023-10003-9 37535155

[18] Zhao Y (2022). Decoding the dopamine transporter imaging for the differential diagnosis of parkinsonism using deep learning. Eur. J. Nucl. Med. Mol. Imaging.

[19] Xu J (2021). Computer-aided classification framework of Parkinsonian disorders using (11)C-CFT PET imaging. Front. Aging Neurosci..

[20] Sun X (2022). Use of deep learning-based radiomics to differentiate Parkinson’s disease patients from normal controls: a study based on [(18)F]FDG PET imaging. Eur. Radiol..

[21] Yoon HJ (2021). Heterogeneity by global and textural feature analysis in F-18 FP-CIT brain PET images for diagnosis of Parkinson’s disease. Medicine.

[22] Piccardo A (2021). The role of the deep convolutional neural network as an aid to interpreting brain [(18)F]DOPA PET/CT in the diagnosis of Parkinson’s disease. Eur. Radiol..

[23] Martins, R. et al. Automatic classification of idiopathic Parkinson’s disease and atypical Parkinsonian syndromes combining [(11)C]raclopride PET uptake and MRI grey matter morphometry. *J. Neural Eng.* **18**, 10.1088/1741-2552/abf772 (2021). 10.1088/1741-2552/abf772 33848996

[24] Hu X (2021). Multivariate radiomics models based on (18)F-FDG hybrid PET/MRI for distinguishing between Parkinson’s disease and multiple system atrophy. Eur. J. Nucl. Med. Mol. Imaging.

[25] Dai Y (2021). Multi-focus image fusion based on convolution neural network for Parkinson’s disease image classification. Diagnostics.

[26] Choi BW (2021). Faster region-based convolutional neural network in the classification of different parkinsonism patterns of the striatum on maximum intensity projection images of [(18)F]FP-CIT positron emission tomography. Diagnostics.

[27] van Veen R (2020). An application of generalized matrix learning vector quantization in neuroimaging. Comput. Methods Prog. Biomed..

[28] Rus T (2020). Differential diagnosis of Parkinsonian syndromes: a comparison of clinical and automated - metabolic brain patterns’ based approach. Eur. J. Nucl. Med. Mol. Imaging.

[29] Wu Y (2019). Use of radiomic features and support vector machine to distinguish Parkinson’s disease cases from normal controls. Ann. Transl. Med..

[30] Shen T (2019). Use of overlapping group LASSO sparse deep belief network to discriminate Parkinson’s disease and normal control. Front. Neurosci..

[31] Manzanera OM (2019). Scaled subprofile modeling and convolutional neural networks for the identification of Parkinson’s disease in 3D nuclear imaging data. Int. J. Neural Syst..

[32] Segovia F, Górriz JM, Ramírez J, Martínez-Murcia FJ, Salas-Gonzalez D (2017). Preprocessing of (18)F-DMFP-PET data based on hidden Markov random fields and the Gaussian distribution. Front. Aging Neurosci..

[33] Segovia F (2017). Multivariate analysis of (18)F-DMFP PET data to assist the diagnosis of parkinsonism. Front. Neuroinform..

[34] Segovia F (2015). Distinguishing Parkinson’s disease from atypical Parkinsonian syndromes using PET data and a computer system based on support vector machines and Bayesian networks. Front. Comput. Neurosci..

[35] Mudali D, Teune LK, Renken RJ, Leenders KL, Roerdink JB (2015). Classification of Parkinsonian syndromes from FDG-PET brain data using decision trees with SSM/PCA features. Comput. Math. Methods Med..

[36] Garraux G (2013). Multiclass classification of FDG PET scans for the distinction between Parkinson’s disease and atypical parkinsonian syndromes. Neuroimage Clin..

[37] Tang CC (2010). Differential diagnosis of parkinsonism: a metabolic imaging study using pattern analysis. Lancet Neurol..

[38] Martinez-Murcia FJ, Górriz JM, Ramírez J, Ortiz A (2018). Convolutional neural networks for neuroimaging in Parkinson’s disease: is preprocessing needed?. Int. J. Neural Syst..

[39] Diaz O (2021). Data preparation for artificial intelligence in medical imaging: a comprehensive guide to open-access platforms and tools. Phys. Med..

[40] Salahuddin Z, Woodruff HC, Chatterjee A, Lambin P (2021). Transparency of deep neural networks for medical image analysis: a review of interpretability methods. Comput. Biol. Med..

[41] van der Velden BHM, Kuijf HJ, Gilhuijs KGA, Viergever MA (2022). Explainable artificial intelligence (XAI) in deep learning-based medical image analysis. Med. Image Anal..

[42] Montavon, G., Binder, A., Lapuschkin, S., Samek, W. & Müller, K.-R. in *Explainable AI: Interpreting, Explaining and Visualizing Deep Learning* (eds Samek W. et al.) 193–209 (Springer International Publishing, 2019).

[43] Fletcher J (2007). What is heterogeneity and is it important?. BMJ.

[44] Balki I (2019). Sample-size determination methodologies for machine learning in medical imaging research: a systematic review. Can. Assoc. Radiol. J..

[45] Tian M (2022). International nuclear medicine consensus on the clinical use of amyloid positron emission tomography in Alzheimer’s disease. Phenomics.

[46] Altman DG, Vergouwe Y, Royston P, Moons KG (2009). Prognosis and prognostic research: validating a prognostic model. BMJ.

[47] Fang H, Shi K, Wang X, Zuo C, Lan X (2022). Editorial: artificial intelligence in positron emission tomography. Front. Med..

[48] Zhang Y, Lu J, Wang M, Zuo C, Jiang J (2022). Influence of gender on tau precipitation in Alzheimer’s disease according to ATN research framework. Phenomics.

[49] Lu J (2023). Adjustment for the age- and gender-related metabolic changes improves the differential diagnosis of parkinsonism. Phenomics.

[50] Rajpurkar P, Lungren MP (2023). The current and future state of AI interpretation of medical images. N. Engl. J. Med..

[51] Houssein NJ, Henriksen AC, Hejl AM, Marner L (2023). Diagnostic accuracy of cerebral [(18)F]FDG PET in atypical parkinsonism. EJNMMI Res..

[52] Gu SC, Ye Q, Yuan CX (2019). Metabolic pattern analysis of (18)F-FDG PET as a marker for Parkinson’s disease: a systematic review and meta-analysis. Rev. Neurosci..

[53] Nandhagopal R (2011). Longitudinal evolution of compensatory changes in striatal dopamine processing in Parkinson’s disease. Brain.

[54] Bauckneht M (2018). Presynaptic dopaminergic neuroimaging in REM sleep behavior disorder: a systematic review and meta-analysis. Sleep. Med. Rev..

[55] Peralta C (2022). Pragmatic approach on neuroimaging techniques for the differential diagnosis of parkinsonisms. Mov. Disord. Clin. Pr..

[56] Page MJ (2021). The PRISMA 2020 statement: an updated guideline for reporting systematic reviews. BMJ.

[57] Sounderajah V (2021). A quality assessment tool for artificial intelligence-centered diagnostic test accuracy studies: QUADAS-AI. Nat. Med..

[58] Jackson D, Turner R (2017). Power analysis for random-effects meta-analysis. Res. Synth. Methods.

[59] Bossuyt, P., Deeks, J., Leeflang, M., Takwoingi, Y. & Flemyng, E. *Cochrane Handbook for Systematic Reviews of Diagnostic Test Accuracy Version 2* (Cochrane, London, 2022). 10.1002/14651858.ED000163 PMC10408284 37470764

