# Supplementary material for: Diagnostic performance of artificial intelligence-assisted PET imaging for Parkinson’s disease: a systematic review and meta-analysis
Source: NPJ Digit Med. 2024 Jan 22;7:17. doi: 10.1038/s41746-024-01012-z (PMC10803804; doi:10.1038/s41746-024-01012-z)
Supplement: Supplementary file 2 — Supplementary materials [file 41746_2024_1012_MOESM2_ESM.pdf]

Supplementary Information

Diagnostic performance of artificial intelligence-assisted PET imaging for Parkinson's disease: a systematic review and meta-analysis

Jing Wang[1,2], Le Xue[3], Jiehui Jiang[4], Fengtao Liu[5,6], Ping Wu[2], Jiaying Lu[2], Huiwei Zhang[2], Weiqi Bao[2], Qian Xu[2], Zizhao Ju[2], Li Chen[7], Fangyang Jiao[2], Huamei Lin[2], Jingjie Ge[2*], Chuantao Zuo[1,2,6*] and Mei Tian[1,2*]

[1]Huashan Hospital & Human Phenome Institute, Fudan University, Shanghai, China.

[2]Department of Nuclear Medicine/PET Center, Huashan Hospital, Fudan University, Shanghai, China.

[3]Department of Nuclear Medicine, the Second Hospital of Zhejiang University School of Medicine, Hangzhou, Zhejiang, China.

[4]Institute of Biomedical Engineering, School of Life Science, Shanghai University, Shanghai, China.

[5]Department of Neurology, Huashan Hospital, Fudan University, Shanghai, China.

[6]National Clinical Research Center for Aging and Medicine, & National Center for Neurological Disorders, Huashan Hospital, Fudan University, Shanghai, China.

[7]Department of Ultrasound Medicine, Huashan Hospital, Fudan University, Shanghai, China.

[*]Corresponding author:

Mei Tian

Chuantao Zuo

Jingjie Ge

Email:

tianmei@fudan.edu.cn

zuochuantao@fudan.edu.cn

lovejingjie@126.com

Table of Contents

Supplementary Table 1. Other characteristic of all included studies (n = 23) 4
Supplementary Table 2. Detailed enumeration for various categories across different PET tracers 7
Supplementary Table 3. Detailed enumeration of various categories across different PET tracers (PD vs. NC) 8
Supplementary Table 4. Detailed enumeration of various categories across different PET tracer (PD vs. AP) 9
Supplementary Table 5. Classification accuracy using different ML algorithms-assisted ^{18}F-FDG PET imaging (PD vs. NC) 11
Supplementary Table 6. Classification accuracy using different ML algorithms-assisted ^{18}F-FDG PET imaging (PD vs. AP) 12
Supplementary Table 7. Classification accuracy using sMRI assisted AI algorithm in ^{18}F-FDG PET imaging (PD vs. NC) 13
Supplementary Table 8. Classification accuracy using sMRI assisted AI algorithm in ^{18}F-FDG PET imaging (PD vs. AP) 14
Supplementary Table 9. Classification accuracy using presynaptic PET imaging (PD vs. NC) 15
Supplementary Table 10. Classification accuracy using presynaptic PET imaging (PD vs. AP) 16
Supplementary Table 11. Tabular presentation of QUADAS-AI 17
Supplementary Table 12. Contingency tables for classifying PD from NC (140 tables from 11 studies) 18
Supplementary Table 13. Contingency tables for classifying PD from AP (73 tables from 13 studies) 24
Supplementary Table 14. Description of quality assessment based on QUADAS-AI domains 28
Supplementary Figure 1. Forest plot using presynaptic DA PET imaging (PD vs. NC) 29
Supplementary Figure 2. Forest plot with highest performance using ^{18}F-FDG PET imaging (PD vs. NC) 30
Supplementary Figure 3. Forest plot using ^{18}F-FDG PET imaging (PD vs. NC) 31
Supplementary Figure 4. Forest plot using DL- and ML-assisted ^{18}F-FDG PET imaging (PD vs. NC) 32
Supplementary Figure 5. Forest plot using SVM-assisted ^{18}F-FDG PET imaging (PD vs. NC) 33
Supplementary Figure 6. Forest plot using additional ML-assisted ^{18}F-FDG PET imaging (PD vs. NC) 34
Supplementary Figure 7. Forest plot using ^{18}F-FDG PET imaging based on sample size (PD vs. NC) 35
Supplementary Figure 8. Forest plot using presynaptic DA PET imaging (PD vs. AP) 36
Supplementary Figure 9. Forest plot with highest performance using presynaptic DA PET imaging (PD vs. AP) 37
Supplementary Figure 10. Forest plot using ^{11}C CFT PET imaging (PD vs. AP) 38
Supplementary Figure 11. Forest plot using DL-assisted presynaptic DA PET imaging (PD vs. AP) 39
Supplementary Figure 12. Forest plot using postsynaptic DA PET imaging (PD vs. AP) 40
Supplementary Figure 13. Forest plot with highest performance using postsynaptic DA PET imaging (PD vs. AP) 41
Supplementary Figure 14. Forest plot using ^{18}F-FDG PET imaging (PD vs. AP) 42
Supplementary Figure 15. Forest plot with highest performance using ^{18}F-FDG PET imaging (PD vs. AP) 43
Supplementary Figure 16. Forest plot using DL- and ML-assisted ^{18}F-FDG PET imaging (PD vs. AP) 44
Supplementary Figure 17. Forest plot using LR-assisted ^{18}F-FDG PET imaging (PD vs. AP) 45
Supplementary Figure 18. Forest plot using ^{18}F-FDG PET imaging based on sample size (PD vs. AP) 46
Supplementary Figure 19. SROC curves using DL- and ML-assisted ^{18}F-FDG PET imaging (PD vs. NC) 47
Supplementary Figure 20. SROC curves using SVM-assisted ^{18}F-FDG PET imaging (PD vs. NC) 48
Supplementary Figure 21. SROC curves using additional ML-assisted ^{18}F-FDG PET imaging (PD vs. NC) 49
Supplementary Figure 22. SROC curves using ^{18}F-FDG PET imaging based on sample size (PD vs. NC) 50
Supplementary Figure 23. SROC curves using DL- and ML-assisted ^{18}F-FDG PET imaging (PD vs. AP) 51
Supplementary Figure 24. SROC curves using ^{18}F-FDG PET imaging based on sample size (PD vs. AP) 52

Supplementary Figure 25. Publication bias using presynaptic DA and ^{18}F-FDG PET imaging (PD vs. NC) 53
Supplementary Figure 26. Publication bias using DL- and ML- assisted ^{18}F-FDG PET imaging (PD vs. NC) 54
Supplementary Figure 27. Publication bias using ML- assisted ^{18}F-FDG PET imaging (PD vs. NC) 55
Supplementary Figure 28. Publication bias using ^{18}F-FDG PET imaging based on sample size (PD vs. NC) 56
Supplementary Figure 29. Publication bias using presynaptic DA PET imaging (PD vs. AP) 57
Supplementary Figure 30. Publication bias using postsynaptic DA and ^{18}F-FDG PET imaging (PD vs. AP) 58
Supplementary Figure 31. Publication bias using DL- and ML- assisted ^{18}F-FDG PET imaging (PD vs. AP) 59
Supplementary Figure 32. Publication bias using LR-assisted ^{18}F-FDG PET imaging (PD vs. AP) 60
Supplementary Figure 33. Publication bias using ^{18}F-FDG PET imaging based on sample size (PD vs. AP) 61
Supplementary Figure 34. QUADAS-AI summary plot and risk of bias and concern of applicability for each item 62
Supplementary Methods. Search terms and search strategy 63
Supplementary Methods. Inclusion/exclusion criteria of literature 70
Supplementary References 71

Supplementary Table 1. Other characteristic of all included studies (n = 23)

Author. year[ref]	Participant demographics							Data characteristics					Algorithm details
	Target condition	Structural brain lesions	Other degenerative diseases excluded	Prior exposure to neuroleptics/ drug use excluded	Neurological / psychiatric illness excluded?	Reference standard	Pathological diagnosis?	Imaging agent	Tracer type	Structural MRI?	Exclusion of poor-quality imaging	Open access data	AI vs visual interpretation?
Sun et al. 2023[12]	PD vs. AP	Yes	Yes	NR	Yes	Expert consensus; two years follow-up	No	^{11}C-CFT<br>^{18}F-FDG	Presynaptic dopamine<br>Glucose metabolism	Yes	Yes	No	No
Wu et al. 2022[13]	PD vs. AP	NR	NR	NR	NR	Expert consensus; two years follow-up	No	^{18}F-FDG	Glucose metabolism	No	NR	No	No
Zhao et al. 2022[14]	PD vs. AP	NR	NR	Yes	Yes	Expert consensus; two years follow-up	No	^{11}C-CFT	Presynaptic dopamine	No	NR	No	No
Xu et al. 2022[15]	PD vs. AP	Yes	NR	Yes	Yes	Expert consensus	No	^{11}C-CFT	Presynaptic dopamine	Yes	NR	No	No
Sun et al. 2022[1]	PD vs. NC	NR	Yes	Yes	Yes	Expert consensus	No	^{18}F-FDG	Glucose metabolism	No	NR	No	No
Yoon et al. 2021[2]	PD vs. NC	Yes	NR	NR	Yes	Expert consensus	No	^{18}F-FP-CIT	Presynaptic dopamine	No	NR	No	No
Piccardo et al. 2021[3]	PD vs. NC	NR	NR	NR	NR	Expert consensus; two years follow-up	No	^{18}F-DOPA	Presynaptic dopamine	No	NR	No	No
Martins et al. 2021[4]	PD vs. NC<br>PD vs. AP	NR	Yes	NR	Yes	Expert consensus	No	^{11}C-RAC	Postsynaptic dopamine	Yes	NR	No	No

Hu et al. 2021[16]	PD vs. AP	Yes	Yes	NR	NR	Expert consensus	No	^{18}F-FDG	Glucose metabolism	Yes	Yes	No	No
Dai et al. 2021[5]	PD vs. NC	NR	NR	NR	NR	Expert consensus	No	^{18}F-FDG	Glucose metabolism	Yes	NR	Yes	Yes
Choi et al. 2021[17]	PD vs. AP	Yes	Yes	NR	NR	Expert consensus	No	^{18}F-FP-CIT	Presynaptic dopamine	No	NR	No	No
Van et al. 2020[6]	PD vs. NC	NR	NR	NR	NR	Expert consensus	No	^{18}F-FDG	Glucose metabolism	No	NR	No	No
Rus et al. 2020[18]	PD vs. AP	NR	NR	NR	NR	Expert consensus； one-year follow-up	No	^{18}F-FDG	Glucose metabolism	No	NR	No	No
Wu et al. 2019[7]	PD vs. NC	NR	NR	Yes	Yes	Expert consensus	No	^{18}F-FDG	Glucose metabolism	No	NR	No	No
Shen et al. 2019[8]	PD vs. NC	NR	NR	Yes	Yes	Expert consensus	No	^{18}F-FDG	Glucose metabolism	No	NR	No	No
Manzanera et al. 2019[9]	PD vs. NC	NR	Yes	NR		Expert consensus	No	^{18}F-FDG	Glucose metabolism	No	NR	No	No
Glaab et al. 2019[10]	PD vs. NC	NR	NR	NR	NR	Expert consensus	No	^{18}F-FDG	Glucose metabolism	No	NR	No	No
Segovia, F et al. 2017[19]	PD vs. AP	NR	NR	NR	NR	Expert consensus; follow-up	No	^{18}F-DMFP	Postsynaptic dopamine	No	NR	No	No
Segovia, F et al. 2017[20]	PD vs. AP	NR	NR	NR	NR	Expert consensus; follow-up	No	^{18}F-DMFP	Postsynaptic dopamine	No	NR	No	No
Segovia, F et al. 2015[21]	PD vs. AP	NR	NR	NR	NR	Expert consensus; follow-up	No	^{18}F-DMFP	Postsynaptic dopamine	No	NR	No	No
Mudali et al. 2015[11]	PD vs. NC	NR	NR	NR	NR	Expert consensus; follow-up	No	^{18}F-FDG	Glucose metabolism	No	NR	No	No

Garraux et al. 2013[22]	PD vs. AP	Yes	NR	Yes	NR	Expert consensus	No	^{18}F-FDG	Glucose metabolism	No	NR	No	No
Tang et al. 2010[23]	PD vs. AP	Yes	NR	NR	NR	Expert consensus; two years follow-up	No	^{18}F-FDG	Glucose metabolism	No	NR	No	No

NR not reported, NC normal control, PD Parkinson's disease, AP atypical parkinsonism, AI artificial intelligence, ^{18}F-DMFP ^{18}F-Desmethoxyfallypride, ^{18}F-FDG ^{18}F-fluorodeoxyglucose, ^{18}F FP-CIT ^{18}F N-(3-fluoropropyl)-2β-carboxymethoxy-3β-(4-iodophenyl) nortropane, ^{11}C-CFT ^{11}C-2β-carbomethoxy-3β-(4-fluorophenyl) tropane, ^{18}F-DOPA ^{18}F-Fluoro-dihydroxyphenylalanine, ^{11}C-RAC ^{11}C-raclopride, MRI magnetic resonance imaging.

Supplementary Table 2. Detailed enumeration for various categories across different PET tracers

Category		Number of studies	Number of tables
PD diagnosis classification	PD from NC	11	140
	PD from AP	13	73
PET imaging agent	Presynaptic dopamine	7	34
	^{11}C-CFT	3	12
	^{18}F-DOPA	2	9
	^{18}F-FPCIT	2	13
	Postsynaptic dopamine	4	18
	^{11}C-raclopride	1	6
	^{18}F-DMFP	3	12
	Glucose metabolism		
	^{18}F-FDG	14	161
AI algorithm	Convolutional Neural Network	8	83
	Machine Learning	17	130
	Support Vector Machine	10	66
	Random Forest	3	15
	XGBoost	1	9
	Decision Tree	2	2
	Logistic Regression	4	14
	GMLVQ	1	10
	LASSO	1	14

Supplementary Table 3. Detailed enumeration of various categories across different PET tracers (PD vs. NC)

Imaging agent	Category	Number of studies	Number of tables
Presynaptic dopamine	Presynaptic dopamine	3	21
	^{18}F-DOPA	2	9
	^{18}F-FPCIT	1	12
	Algorithm		
	DL	0	0
	ML	3	21
	Sample size		
	≥100	0	0
	<100	3	21
	Structural MRI		
	Yes	0	0
	No	0	0
Postsynaptic dopamine	^{11}C-raclopride	1	3
Glucose metabolism	^{18}F-FDG	8	116
	Algorithm		
	DL	4	53
	ML	6	63
	Sample size		
	≥100	5	46
	<100	7	70
	Structural MRI		
	Yes	1	4
	No	7	112

Supplementary Table 4. Detailed enumeration of various categories across different PET tracer (PD vs. AP)

Imaging agent	Category	Number of studies	Number of tables
Presynaptic dopamine	Presynaptic dopamine	4	13
	^{11}C-CFT	3	12
	^{18}F-FPCIT	1	1
	Algorithm		
	DL	2	10
	ML	2	3
	Sample size		
	≥100	2	6
	<100	3	7
	Structural MRI		
	Yes	2	3
	No	2	10
Postsynaptic dopamine	Postsynaptic dopamine	4	15
	^{11}C-raclopride	1	3
	^{18}F-DMFP	3	12
	Algorithm		
	DL	0	0
	ML	4	15
	Sample size		
	≥100	0	0
	<100	4	15
	Structural MRI		
	Yes	1	3

	No	3	12
Glucose metabolism	Glucose metabolism	6	45
	^{18}F-FDG	6	45
	Algorithm		
	DL	1	19
	ML	5	26
	Sample size		
	≥100	3	21
	<100	5	24
	Structural MRI		
	Yes	2	16
	No	4	29

Supplementary Table 5. Classification accuracy using different ML algorithms-assisted ^{18}F-FDG PET imaging (PD vs. NC)

Category	No. of tables	AUC (95% CI)	Sensitivity (%)		*p* value [a]	Specificity (%)		*p* value [a]
			SE (95% CI)	I^2 (95% CI)		SP (95% CI)	I^2 (95% CI)	
SVM	42	0.88 (0.85−0.91)	77.66 (73.49−81.34)	74.89 (67.39−82.39)	< 0.001	86.67 (82.26−90.12)	88.51 (85.76−91.26)	0.53
Additional	21	0.85 (0.82−0.88)	82.88 (77.13−87.46)	76.85 (67.23−86.47)		70.52 (53.67−83.16)	90.35 (87.22−93.48)	
RF	10	-	-	-		-	-	
LVQ	10	-	-	-		-	-	
DT	1	-	-	-		-	-	

[a] *p* value for heterogeneity between subgroups with meta-regression analysis.

Supplementary Table 6. Classification accuracy using different ML algorithms-assisted ^{18}F-FDG PET imaging (PD vs. AP)

Category	No. of tables	AUC (95% CI)	Sensitivity (%)		Specificity (%)	
			SE (95% CI)	I^2 (95% CI)	SP (95% CI)	I^2 (95% CI)
LR	11	0.91 (0.88−0.93)	83.72 (79.26−87.39)	32.98 (0.00−80.60)	95.75 (90.23−98.22)	43.24 (3.29−83.19)
LASSO	14	-	-	-	-	-
DT	1	-	-	-	-	-

Supplementary Table 7. Classification accuracy using sMRI assisted AI algorithm in ^{18}F-FDG PET imaging (PD vs. NC)

Category	No. of tables	AUC (95% CI)	Sensitivity (%)		*p* value [a]	Specificity (%)		*p* value [a]
			SE (95% CI)	I^2 (95% CI)		SP (95% CI)	I^2 (95% CI)	
Structural MRI					0.09			0.65
Yes	4	0.98 (0.96−0.99)	94.16 (88.78−97.04)	95.52 (96.18−98.87)		92.35 (85.40−96.15)	96.60 (95.09−98.71)	
No	112	0.89 (0.86−0.92)	82.87 (80.63−84.90)	73.92 (69.11−78.72)		83.36 (80.10−86.18)	89.09 (87.52−90.65)	

[a] *p* value for heterogeneity between subgroups with meta-regression analysis.

Supplementary Table 8. Classification accuracy using sMRI assisted AI algorithm in ^{18}F-FDG PET imaging (PD vs. AP)

Category	No. of tables	AUC (95% CI)	Sensitivity (%)		*p* value [a]	Specificity (%)		*p* value [a]
			SE (95% CI)	I^2 (95% CI)		SP (95% CI)	I^2 (95% CI)	
Structural MRI					< 0.001			0.65
Yes	16	0.93 (0.91−0.95)	89.63 (85.49−92.69)	43.45 (10.17−76.73)		84.02 (74.42−90.49)	59.13 (36.61−81.65)	
No	29	0.98 (0.97−0.99)	94.03 (91.63−95.77)	79.69 (72.79−86.60)		95.25 (93.28−96.67)	58.39 (41.31−75.48)	

[a] *p* value for heterogeneity between subgroups with meta-regression analysis.

Supplementary Table 9. Classification accuracy using presynaptic PET imaging (PD vs. NC)

Category	No. of tables	AUC (95% CI)	Sensitivity (%)		*p* value [a]	Specificity (%)		*p* value [a]
			SE (95% CI)	I^2 (95% CI)		SP (95% CI)	I^2 (95% CI)	
^{18}F-FPCIT	12	0.96 (0.94−0.97)	92.14 (83.68−96.40)	87.46 (81.58−93.35)	< 0.001	93.32 (89.82−95.67)	17.63 (0.00−71.11)	0.77
^{18}F-DOPA	9	0.92 (0.89−0.94)	90.77 (87.15−93.44)	0.00 (0.00−100.0)		72.86 (62.90−80.95)	26.75 (0.00−82.57)	

[a] p value for heterogeneity between subgroups with meta-regression analysis.

Supplementary Table 10. Classification accuracy using presynaptic PET imaging (PD vs. AP)

Category	No. of tables	AUC (95% CI)	Sensitivity (%)		Specificity (%)	
			SE (95% CI)	I^2 (95% CI)	SP (95% CI)	I^2 (95% CI)
^{11}C-CFT	12	0.93 (0.90−0.95)	89.39 (86.87−91.47)	41.97 (2.66−81.28)	89.52 (82.07−94.10)	80.64 (70.39−90.89)
^{18}F-DOPA	1	-	-	-	-	-

Supplementary Table 11. Tabular presentation of QUADAS-AI

Author [ref], year	Subject selection						Index Test		Reference standard		Flow and Timing	
	Q1	Q2	Q3	Q4	Q5	Risk	Q6	Risk	Q7	Risk	Q8	Risk
Sun et al. 2023[12]	Yes	No	Yes	Yes	Yes	Low	No	High	Yes	Low	Yes	Low
Wu et al. 2022[13]	Yes	No	Yes	Yes	Yes	Low	Yes	Low	Yes	Low	Yes	Low
Zhao et al. 2022[14]	Yes	No	Yes	Yes	Yes	Low	No	High	Yes	Low	Yes	Low
Xu et al. 2022[15]	Yes	No	No	Yes	Yes	High	No	High	Yes	Low	Yes	Low
Sun et al. 2022[1]	Yes	No	Yes	Yes	Yes	Low	Yes	Low	Yes	Low	Yes	Low
Yoon et al. 2021[2]	Yes	No	No	Unclear	Yes	High	No	High	Yes	Low	Yes	Low
Piccardo et al. 2021[3]	Yes	No	Yes	Unclear	No	Unclear	No	High	Unclear	Unclear	Yes	Low
Martins et al. 2021[4]	Yes	No	No	Yes	Yes	High	No	High	Yes	Low	Yes	Low
Hu et al. 2021[16]	No	No	Yes	Yes	Yes	High	No	High	Yes	Low	Yes	Low
Dai et al. 2021[5]	Yes	Yes	Yes	Yes	No	Low	No	High	Yes	Low	Yes	Low
Choi et al. 2021[17]	Yes	No	Yes	No	Yes	High	No	High	Yes	Low	Yes	Low
Van et al. 2020[6]	Yes	No	Yes	Yes	No	High	No	High	Yes	Low	Yes	Low
Rus et al. 2020[18]	Yes	No	Yes	Yes	Yes	Low	No	High	Yes	Low	Yes	Low
Wu et al. 2019[7]	Yes	No	Yes	Yes	Yes	Low	Yes	Low	Yes	Low	Yes	Low
Shen et al. 2019[8]	Yes	No	Yes	Yes	Yes	Low	Yes	Low	Yes	Low	Yes	Low
Manzanera et al. 2019[9]	Yes	No	Yes	Yes	Yes	Low	No	High	Yes	Low	Yes	Low
Glaab et al. 2019[10]	Yes	No	Yes	Yes	Yes	Low	No	High	Yes	Low	Yes	Low
Segovia, F et al. 2017[19]	No	No	No	Yes	No	High	No	High	Yes	Low	Unclear	Unclear
Segovia, F et al. 2017[20]	No	No	No	Yes	No	High	No	High	Yes	Low	Unclear	Unclear
Segovia, F et al. 2015[21]	No	No	No	Yes	No	High	No	High	Yes	Low	Unclear	Unclear
Mudali et al. 2015[11]	Yes	No	Yes	Unclear	Yes	High	No	High	Yes	Low	Yes	Low
Garraux et al. 2013[22]	Yes	No	Yes	Yes	Yes	Low	No	High	Yes	Low	Yes	Low
Tang et al. 2010[23]	Yes	No	Yes	Yes	No	High	No	High	Yes	Low	Yes	Low

QUADAS-AI quality assessment of diagnostic accuracy studies-AI

Supplementary Table 12. Contingency tables for classifying PD from NC (140 tables from 11 studies)

Author [ref]	Year	Total	PD	NC	TP	TN	FN	FP	Accuracy	SE	SP	AUROC	ML/DL		Imaging agent	
Sun et al [1]	2022	358	103	255	74	194	29	61	0.749	0.718	0.761	0.720	ML	SVM	^{18}F-FDG	Glucose metabolism
Sun et al [1]	2022	358	103	255	90	239	13	16	0.919	0.874	0.937	0.840	ML	SVM	^{18}F-FDG	Glucose metabolism
Sun et al [1]	2022	358	103	255	83	235	20	20	0.888	0.806	0.922	0.820	ML	SVM	^{18}F-FDG	Glucose metabolism
Sun et al [1]	2022	358	103	255	92	249	11	6	0.953	0.893	0.976	0.900	DL	CNN	^{18}F-FDG	Glucose metabolism
Sun et al [1]	2022	305	50	255	34	187	16	68	0.725	0.680	0.733	0.720	ML	SVM	^{18}F-FDG	Glucose metabolism
Sun et al [1]	2022	305	50	255	39	212	11	43	0.823	0.780	0.831	0.770	ML	SVM	^{18}F-FDG	Glucose metabolism
Sun et al [1]	2022	305	50	255	36	200	14	55	0.774	0.720	0.784	0.740	ML	SVM	^{18}F-FDG	Glucose metabolism
Sun et al [1]	2022	305	50	255	41	219	9	36	0.852	0.820	0.859	0.830	DL	CNN	^{18}F-FDG	Glucose metabolism
Sun et al [1]	2022	308	53	255	36	205	17	50	0.782	0.679	0.804	0.740	ML	SVM	^{18}F-FDG	Glucose metabolism
Sun et al [1]	2022	308	53	255	46	240	7	15	0.929	0.868	0.941	0.870	ML	SVM	^{18}F-FDG	Glucose metabolism
Sun et al [1]	2022	308	53	255	41	243	12	12	0.922	0.774	0.953	0.830	ML	SVM	^{18}F-FDG	Glucose metabolism
Sun et al [1]	2022	308	53	255	46	249	7	6	0.958	0.868	0.976	0.920	DL	CNN	^{18}F-FDG	Glucose metabolism
Sun et al [1]	2022	358	103	255	99	248	4	7	0.969	0.961	0.973	NR	DL	CNN	^{18}F-FDG	Glucose metabolism
Sun et al [1]	2022	358	103	255	92	240	11	15	0.927	0.893	0.941	NR	DL	CNN	^{18}F-FDG	Glucose metabolism
Sun et al [1]	2022	358	103	255	94	238	9	17	0.927	0.913	0.933	NR	DL	CNN	^{18}F-FDG	Glucose metabolism
Sun et al [1]	2022	358	103	255	95	242	8	13	0.941	0.922	0.949	NR	DL	CNN	^{18}F-FDG	Glucose metabolism
Sun et al [1]	2022	358	103	255	96	248	7	7	0.961	0.932	0.973	NR	DL	CNN	^{18}F-FDG	Glucose metabolism
Sun et al [1]	2022	48	22	26	15	19	7	7	0.708	0.682	0.731	0.660	ML	SVM	^{18}F-FDG	Glucose metabolism
Sun et al [1]	2022	48	22	26	18	23	4	3	0.854	0.818	0.885	0.770	ML	SVM	^{18}F-FDG	Glucose metabolism
Sun et al [1]	2022	48	22	26	15	23	7	3	0.792	0.682	0.885	0.700	ML	SVM	^{18}F-FDG	Glucose metabolism
Sun et al [1]	2022	48	22	26	19	23	3	3	0.875	0.864	0.885	0.810	DL	CNN	^{18}F-FDG	Glucose metabolism
Sun et al [1]	2022	38	12	26	8	19	4	7	0.711	0.667	0.731	0.650	ML	SVM	^{18}F-FDG	Glucose metabolism

Sun et al [1]	2022	38	12	26	9	21	3	5	0.789	0.750	0.808	0.750	ML	SVM	^{18}F-FDG	Glucose metabolism
Sun et al [1]	2022	38	12	26	8	20	4	6	0.737	0.667	0.769	0.640	ML	SVM	^{18}F-FDG	Glucose metabolism
Sun et al [1]	2022	38	12	26	10	23	2	3	0.868	0.833	0.885	0.800	DL	CNN	^{18}F-FDG	Glucose metabolism
Sun et al [1]	2022	36	10	26	7	19	3	7	0.722	0.700	0.731	0.690	ML	SVM	^{18}F-FDG	Glucose metabolism
Sun et al [1]	2022	36	10	26	8	19	2	7	0.750	0.800	0.731	0.820	ML	SVM	^{18}F-FDG	Glucose metabolism
Sun et al [1]	2022	36	10	26	7	21	3	5	0.778	0.700	0.808	0.790	ML	SVM	^{18}F-FDG	Glucose metabolism
Sun et al [1]	2022	36	10	26	8	23	2	3	0.861	0.800	0.885	0.850	DL	CNN	^{18}F-FDG	Glucose metabolism
Sun et al [1]	2022	48	22	26	19	23	3	3	0.875	0.864	0.885	NR	DL	CNN	^{18}F-FDG	Glucose metabolism
Sun et al [1]	2022	48	22	26	20	22	2	4	0.875	0.909	0.846	NR	DL	CNN	^{18}F-FDG	Glucose metabolism
Sun et al [1]	2022	48	22	26	18	20	4	6	0.792	0.818	0.769	NR	DL	CNN	^{18}F-FDG	Glucose metabolism
Sun et al [1]	2022	48	22	26	19	23	3	3	0.875	0.864	0.885	NR	DL	CNN	^{18}F-FDG	Glucose metabolism
Sun et al [1]	2022	48	22	26	19	23	3	3	0.875	0.864	0.885	NR	DL	CNN	^{18}F-FDG	Glucose metabolism
Yoon et al [2]	2021	62	31	31	26	27	5	4	0.855	0.839	0.871	NR	ML	SVM	^{18}F-FP-CIT	Presynaptic dopamine
Yoon et al [2]	2021	62	31	31	28	28	3	3	0.903	0.903	0.903	NR	ML	LR	^{18}F-FP-CIT	Presynaptic dopamine
Yoon et al [2]	2021	62	31	31	27	28	4	3	0.887	0.871	0.903	NR	ML	RF	^{18}F-FP-CIT	Presynaptic dopamine
Yoon et al [2]	2021	62	31	31	30	29	1	2	0.952	0.968	0.935	NR	ML	XGBoost	^{18}F-FP-CIT	Presynaptic dopamine
Yoon et al [2]	2021	62	31	31	29	29	2	2	0.935	0.935	0.935	0.954	ML	XGBoost	^{18}F-FP-CIT	Presynaptic dopamine
Yoon et al [2]	2021	62	31	31	29	28	2	3	0.919	0.935	0.903	0.956	ML	XGBoost	^{18}F-FP-CIT	Presynaptic dopamine
Yoon et al [2]	2021	62	31	31	30	30	1	1	0.968	0.968	0.968	0.996	ML	XGBoost	^{18}F-FP-CIT	Presynaptic dopamine
Yoon et al [2]	2021	62	31	31	30	30	1	1	0.968	0.968	0.968	0.996	ML	XGBoost	^{18}F-FP-CIT	Presynaptic dopamine
Yoon et al [2]	2021	62	31	31	30	29	1	2	0.952	0.968	0.935	0.990	ML	XGBoost	^{18}F-FP-CIT	Presynaptic dopamine
Yoon et al [2]	2021	62	31	31	31	31	0	0	1.000	1.000	1.000	1.000	ML	XGBoost	^{18}F-FP-CIT	Presynaptic dopamine
Yoon et al [2]	2021	62	31	31	21	30	10	1	0.823	0.677	0.968	0.859	ML	XGBoost	^{18}F-FP-CIT	Presynaptic dopamine
Yoon et al [2]	2021	62	31	31	16	27	16	4	0.694	0.500	0.871	0.689	ML	XGBoost	^{18}F-FP-CIT	Presynaptic dopamine
Piccardo et al [3]	2021	30	11	19	11	17	0	2	0.933	1.000	0.895	0.882	DL	CNN	^{18}F-DOPA	Presynaptic dopamine

Martins et al [4]	2021	42	27	15	27	13	0	2	0.952	1.000	0.867	9.650	ML	SVM	^{11}C-raclopride	Postsynaptic dopamine
Martins et al [4]	2021	42	27	15	15	13	12	2	0.667	0.556	0.867	0.693	ML	SVM	^{11}C-raclopride	Postsynaptic dopamine
Martins et al [4]	2021	42	27	15	26	15	1	0	0.976	0.963	1.000	0.995	ML	SVM	^{11}C-raclopride	Postsynaptic dopamine
Dai et al [5]	2021	1350	614	736	515	645	99	91	0.859	0.839	0.876	0.971	DL	CNN	^{18}F-FDG	Glucose metabolism
Dai et al [5]	2021	1350	614	736	580	629	24	107	0.896	0.960	0.855	0.986	DL	CNN	^{18}F-FDG	Glucose metabolism
Dai et al [5]	2021	1350	614	736	579	685	35	51	0.936	0.943	0.931	0.989	DL	CNN	^{18}F-FDG	Glucose metabolism
Dai et al [5]	2021	1350	614	736	596	718	18	18	0.973	0.971	0.976	0.989	DL	CNN	^{18}F-FDG	Glucose metabolism
Van et al [6]	2020	214	126	88	108	69	18	19	0.827	0.857	0.784	0.890	ML	GMLVQ	^{18}F-FDG	Glucose metabolism
Van et al [6]	2020	214	126	88	102	71	24	17	0.808	0.810	0.807	0.810	ML	GMLVQ	^{18}F-FDG	Glucose metabolism
Van et al [6]	2020	214	126	88	93	64	33	24	0.734	0.738	0.727	0.820	ML	GMLVQ	^{18}F-FDG	Glucose metabolism
Van et al [6]	2020	214	126	88	89	66	37	22	0.724	0.706	0.750	0.310	ML	GMLVQ	^{18}F-FDG	Glucose metabolism
Van et al [6]	2020	39	20	19	16	17	4	2	0.846	0.800	0.895	0.960	ML	GMLVQ	^{18}F-FDG	Glucose metabolism
Van et al [6]	2020	39	20	19	15	17	5	2	0.821	0.750	0.895	0.920	ML	GMLVQ	^{18}F-FDG	Glucose metabolism
Van et al [6]	2020	87	38	49	26	37	12	12	0.724	0.684	0.755	0.790	ML	GMLVQ	^{18}F-FDG	Glucose metabolism
Van et al [6]	2020	87	38	49	25	34	13	15	0.678	0.658	0.694	0.760	ML	GMLVQ	^{18}F-FDG	Glucose metabolism
Van et al [6]	2020	88	68	20	60	16	8	4	0.864	0.882	0.800	0.890	ML	GMLVQ	^{18}F-FDG	Glucose metabolism
Van et al [6]	2020	88	68	20	48	14	20	6	0.705	0.706	0.700	0.770	ML	GMLVQ	^{18}F-FDG	Glucose metabolism
Wu et al [7]	2019	182	91	91	82	84	9	7	0.912	0.901	0.923	0.822	ML	SVM	^{18}F-FDG	Glucose metabolism
Wu et al [7]	2019	182	91	91	73	84	18	7	0.863	0.802	0.923	0.807	ML	SVM	^{18}F-FDG	Glucose metabolism
Wu et al [7]	2019	182	91	91	82	83	9	8	0.907	0.901	0.912	0.870	ML	SVM	^{18}F-FDG	Glucose metabolism
Wu et al [7]	2019	182	91	91	81	87	10	4	0.923	0.890	0.956	0.851	ML	SVM	^{18}F-FDG	Glucose metabolism
Wu et al [7]	2019	182	91	91	73	82	18	9	0.852	0.802	0.901	0.783	ML	SVM	^{18}F-FDG	Glucose metabolism
Wu et al [7]	2019	182	91	91	81	85	10	6	0.912	0.890	0.934	0.876	ML	SVM	^{18}F-FDG	Glucose metabolism
Wu et al [7]	2019	182	91	91	81	82	10	9	0.896	0.890	0.901	0.848	ML	SVM	^{18}F-FDG	Glucose metabolism
Wu et al [7]	2019	182	91	91	75	85	16	6	0.879	0.824	0.934	0.804	ML	SVM	^{18}F-FDG	Glucose metabolism

Wu et al [7]	2019	182	91	91	81	84	10	7	0.907	0.890	0.923	0.871	ML	SVM	^{18}F-FDG	Glucose metabolism
Wu et al [7]	2019	182	91	91	80	81	11	10	0.885	0.879	0.890	NR	ML	RF	^{18}F-FDG	Glucose metabolism
Wu et al [7]	2019	182	91	91	78	76	13	15	0.846	0.857	0.835	NR	ML	RF	^{18}F-FDG	Glucose metabolism
Wu et al [7]	2019	182	91	91	81	84	10	7	0.907	0.890	0.923	NR	ML	RF	^{18}F-FDG	Glucose metabolism
Wu et al [7]	2019	48	22	26	18	23	4	3	0.854	0.818	0.885	NR	ML	SVM	^{18}F-FDG	Glucose metabolism
Wu et al [7]	2019	48	22	26	15	23	7	3	0.792	0.682	0.885	NR	ML	SVM	^{18}F-FDG	Glucose metabolism
Wu et al [7]	2019	48	22	26	18	25	4	1	0.896	0.818	0.962	NR	ML	SVM	^{18}F-FDG	Glucose metabolism
Wu et al [7]	2019	48	22	26	17	25	5	1	0.875	0.773	0.962	NR	ML	SVM	^{18}F-FDG	Glucose metabolism
Wu et al [7]	2019	48	22	26	16	24	6	2	0.833	0.727	0.923	NR	ML	SVM	^{18}F-FDG	Glucose metabolism
Wu et al [7]	2019	48	22	26	18	24	4	2	0.875	0.818	0.923	NR	ML	SVM	^{18}F-FDG	Glucose metabolism
Wu et al [7]	2019	48	22	26	17	25	5	1	0.875	0.773	0.962	NR	ML	SVM	^{18}F-FDG	Glucose metabolism
Wu et al [7]	2019	48	22	26	16	22	6	4	0.792	0.727	0.846	NR	ML	SVM	^{18}F-FDG	Glucose metabolism
Wu et al [7]	2019	48	22	26	18	24	4	2	0.875	0.818	0.923	NR	ML	SVM	^{18}F-FDG	Glucose metabolism
Wu et al [7]	2019	48	22	26	18	24	4	2	0.875	0.818	0.923	NR	ML	RF	^{18}F-FDG	Glucose metabolism
Wu et al [7]	2019	48	22	26	14	24	8	2	0.792	0.636	0.923	NR	ML	RF	^{18}F-FDG	Glucose metabolism
Wu et al [7]	2019	48	22	26	18	24	4	2	0.875	0.818	0.923	NR	ML	RF	^{18}F-FDG	Glucose metabolism
Shen et al [8]	2019	250	75	175	73	140	2	35	0.852	0.973	0.800	0.933	DL	DBN	^{18}F-FDG	Glucose metabolism
Shen et al [8]	2019	250	75	175	59	120	16	55	0.716	0.787	0.686	0.777	DL	DBN	^{18}F-FDG	Glucose metabolism
Shen et al [8]	2019	250	75	175	70	144	5	31	0.856	0.933	0.823	0.928	DL	DBN	^{18}F-FDG	Glucose metabolism
Shen et al [8]	2019	250	75	175	54	120	21	55	0.696	0.720	0.686	0.809	DL	DBN	^{18}F-FDG	Glucose metabolism
Shen et al [8]	2019	250	75	175	67	133	8	42	0.800	0.893	0.760	0.898	DL	DBN	^{18}F-FDG	Glucose metabolism
Shen et al [8]	2019	250	75	175	66	139	9	36	0.820	0.880	0.794	0.998	DL	DBN	^{18}F-FDG	Glucose metabolism
Shen et al [8]	2019	250	75	175	61	130	14	45	0.764	0.813	0.743	0.823	DL	DBN	^{18}F-FDG	Glucose metabolism
Shen et al [8]	2019	250	75	175	70	138	5	37	0.832	0.933	0.789	0.901	DL	DBN	^{18}F-FDG	Glucose metabolism
Shen et al [8]	2019	250	75	175	72	146	3	29	0.872	0.960	0.834	0.963	DL	DBN	^{18}F-FDG	Glucose metabolism

Shen et al [8]	2019	50	25	25	24	21	1	4	0.900	0.960	0.840	0.912	DL	DBN	^{18}F-FDG	Glucose metabolism
Shen et al [8]	2019	50	25	25	18	15	7	10	0.660	0.720	0.600	0.757	DL	DBN	^{18}F-FDG	Glucose metabolism
Shen et al [8]	2019	50	25	25	23	18	2	8	0.820	0.920	0.692	0.910	DL	DBN	^{18}F-FDG	Glucose metabolism
Shen et al [8]	2019	50	25	25	18	17	7	8	0.700	0.720	0.680	0.774	DL	DBN	^{18}F-FDG	Glucose metabolism
Shen et al [8]	2019	50	25	25	24	13	1	13	0.740	0.960	0.500	0.913	DL	DBN	^{18}F-FDG	Glucose metabolism
Shen et al [8]	2019	50	25	25	18	19	7	6	0.740	0.720	0.760	0.810	DL	DBN	^{18}F-FDG	Glucose metabolism
Shen et al [8]	2019	50	25	25	18	18	7	7	0.720	0.720	0.720	0.782	DL	DBN	^{18}F-FDG	Glucose metabolism
Shen et al [8]	2019	50	25	25	18	22	7	3	0.800	0.720	0.880	0.832	DL	DBN	^{18}F-FDG	Glucose metabolism
Shen et al [8]	2019	50	25	25	23	21	2	4	0.880	0.920	0.840	0.932	DL	DBN	^{18}F-FDG	Glucose metabolism
Shen et al [8]	2019	50	25	25	23	20	2	5	0.860	0.920	0.800	0.899	DL	DBN	^{18}F-FDG	Glucose metabolism
Shen et al [8]	2019	50	25	25	17	17	8	8	0.680	0.680	0.680	0.741	DL	DBN	^{18}F-FDG	Glucose metabolism
Shen et al [8]	2019	50	25	25	24	14	1	11	0.760	0.960	0.560	0.873	DL	DBN	^{18}F-FDG	Glucose metabolism
Shen et al [8]	2019	50	25	25	18	18	7	7	0.720	0.720	0.720	0.790	DL	DBN	^{18}F-FDG	Glucose metabolism
Shen et al [8]	2019	50	25	25	24	11	1	14	0.700	0.960	0.440	0.873	DL	DBN	^{18}F-FDG	Glucose metabolism
Shen et al [8]	2019	50	25	25	20	20	5	5	0.800	0.800	0.800	0.875	DL	DBN	^{18}F-FDG	Glucose metabolism
Shen et al [8]	2019	50	25	25	18	20	7	5	0.760	0.720	0.800	0.802	DL	DBN	^{18}F-FDG	Glucose metabolism
Shen et al [8]	2019	50	25	25	18	21	7	4	0.780	0.720	0.840	0.816	DL	DBN	^{18}F-FDG	Glucose metabolism
Shen et al [8]	2019	50	25	25	22	20	3	5	0.840	0.880	0.800	0.895	DL	DBN	^{18}F-FDG	Glucose metabolism
Manzanera et al [9]	2019	40	20	20	17	16	3	4	0.835	0.870	0.800	0.940	DL	CNN	^{18}F-FDG	Glucose metabolism
Manzanera et al [9]	2019	40	20	20	16	16	4	4	0.805	0.790	0.820	0.900	DL	CNN	^{18}F-FDG	Glucose metabolism
Manzanera et al [9]	2019	40	20	20	16	18	4	2	0.840	0.790	0.890	0.920	DL	CNN	^{18}F-FDG	Glucose metabolism
Manzanera et al [9]	2019	40	20	20	17	17	3	3	0.860	0.870	0.850	0.940	DL	CNN	^{18}F-FDG	Glucose metabolism
Manzanera et al [9]	2019	40	20	20	17	17	3	3	0.855	0.840	0.870	0.910	DL	CNN	^{18}F-FDG	Glucose metabolism
Manzanera et al [9]	2019	40	20	20	16	17	4	3	0.820	0.800	0.840	0.910	DL	CNN	^{18}F-FDG	Glucose metabolism
Manzanera et al [9]	2019	40	20	20	8	20	12	0	0.685	0.390	0.980	0.690	ML	SVM	^{18}F-FDG	Glucose metabolism

Manzanera et al [9]	2019	40	20	20	0	20	20	0	0.500	0.000	1.000	0.500	ML	SVM	^{18}F-FDG	Glucose metabolism
Glaab et al [10]	2019	67	51	16	33	2	18	14	0.522	0.647	0.125	0.800	ML	SVM	^{18}F-FDG	Glucose metabolism
Glaab et al [10]	2019	67	51	16	40	7	11	9	0.701	0.784	0.438	NR	ML	SVM	^{18}F-FDG	Glucose metabolism
Glaab et al [10]	2019	67	51	16	48	1	3	15	0.731	0.941	0.063	NR	ML	RF	^{18}F-FDG	Glucose metabolism
Glaab et al [10]	2019	67	51	16	48	2	3	14	0.746	0.941	0.125	NR	ML	RF	^{18}F-FDG	Glucose metabolism
Glaab et al [10]	2019	66	51	15	36	4	15	11	0.606	0.706	0.267	0.910	ML	SVM	^{18}F-FDG	Glucose metabolism
Glaab et al [10]	2019	66	51	15	41	6	10	9	0.712	0.804	0.400	NR	ML	SVM	^{18}F-FDG	Glucose metabolism
Glaab et al [10]	2019	66	51	15	50	1	1	14	0.773	0.980	0.067	NR	ML	RF	^{18}F-FDG	Glucose metabolism
Glaab et al [10]	2019	66	51	15	48	1	3	14	0.742	0.941	0.067	NR	ML	RF	^{18}F-FDG	Glucose metabolism
Glaab et al [10]	2019	58	44	14	39	7	5	7	0.793	0.886	0.500	0.940	ML	SVM	^{18}F-FDOPA	Presynaptic dopamine
Glaab et al [10]	2019	58	44	14	41	9	3	5	0.862	0.932	0.643	NR	ML	SVM	^{18}F-FDOPA	Presynaptic dopamine
Glaab et al [10]	2019	58	44	14	39	10	5	4	0.845	0.886	0.714	NR	ML	RF	^{18}F-FDOPA	Presynaptic dopamine
Glaab et al [10]	2019	58	44	14	39	8	5	6	0.810	0.886	0.571	NR	ML	RF	^{18}F-FDOPA	Presynaptic dopamine
Glaab et al [10]	2019	58	44	14	39	10	5	4	0.845	0.886	0.714	0.980	ML	SVM	^{18}F-FDOPA	Presynaptic dopamine
Glaab et al [10]	2019	58	44	14	39	10	5	4	0.845	0.886	0.714	NR	ML	SVM	^{18}F-FDOPA	Presynaptic dopamine
Glaab et al [10]	2019	58	44	14	41	12	3	2	0.914	0.932	0.857	NR	ML	RF	^{18}F-FDOPA	Presynaptic dopamine
Glaab et al [10]	2019	58	44	14	41	12	3	2	0.914	0.932	0.857	NR	ML	RF	^{18}F-FDOPA	Presynaptic dopamine
Mudali et al [11]	2015	38	20	18	10	8	10	10	0.474	0.500	0.444	NR	ML	DT	^{18}F-FDG	Glucose metabolism

SE Sensitivity, SP Specificity, NR not reported, AI artificial intelligence, DL deep learning, ML machine learning.

Supplementary Table 13. Contingency tables for classifying PD from AP (73 tables from 13 studies)

Author [ref]	Year	Total	PD	AP	AP subtypes			TP	TN	FN	FP	Accuracy	SE	SP	AUROC	ML/DL		Imaging agent	
					MSA	PSP	Others												
Sun et al [12]	2023	84	57	27	27	0	0	49	15	8	12	0.762	0.860	0.556	0.779	ML	LR	^{11}C-CFT	Presynaptic dopamine
Sun et al [12]	2023	84	57	27	27	0	0	47	22	10	5	0.821	0.825	0.815	0.902	ML	LR	^{11}C-CFT	Glucose metabolism
Sun et al [12]	2023	35	24	11	11	0	0	17	6	7	5	0.657	0.708	0.545	0.583	ML	LR	^{18}F-FDG	Presynaptic dopamine
Sun et al [12]	2023	35	24	11	11	0	0	19	10	5	1	0.829	0.792	0.909	0.777	ML	LR	^{18}F-FDG	Glucose metabolism
Wu et al [13]	2022	547	299	248	150	98	0	286	242	13	6	0.966	0.957	0.976	0.986	DL	CNN	^{18}F-FDG	Glucose metabolism
Wu et al [13]	2022	260	136	124	90	34	0	129	121	7	3	0.962	0.949	0.976	0.981	DL	CNN	^{18}F-FDG	Glucose metabolism
Wu et al [13]	2022	287	163	124	60	64	0	156	122	7	2	0.969	0.957	0.984	0.991	DL	CNN	^{18}F-FDG	Glucose metabolism
Wu et al [13]	2022	330	211	119	61	58	0	207	107	4	12	0.952	0.981	0.900	NR	DL	CNN	^{18}F-FDG	Glucose metabolism
Wu et al [13]	2022	108	66	42	22	20	0	65	37	1	5	0.945	0.985	0.881	NR	DL	CNN	^{18}F-FDG	Glucose metabolism
Wu et al [13]	2022	108	66	42	22	20	0	63	41	3	1	0.963	0.955	0.976	NR	DL	CNN	^{18}F-FDG	Glucose metabolism
Wu et al [13]	2022	90	34	56	17	39	0	32	47	2	9	0.878	0.941	0.840	NR	DL	CNN	^{18}F-FDG	Glucose metabolism
Wu et al [13]	2022	330	211	119	61	58	0	193	112	18	7	0.924	0.914	0.941	NR	DL	CNN	^{18}F-FDG	Glucose metabolism
Wu et al [13]	2022	108	66	42	22	20	0	58	40	8	2	0.907	0.878	0.952	NR	DL	CNN	^{18}F-FDG	Glucose metabolism
Wu et al [13]	2022	108	66	42	22	20	0	57	42	9	0	0.917	0.864	0.999	NR	DL	CNN	^{18}F-FDG	Glucose metabolism
Wu et al [13]	2022	330	211	119	61	58	0	207	107	4	12	0.952	0.981	0.900	NR	DL	CNN	^{18}F-FDG	Glucose metabolism
Wu et al [13]	2022	330	211	119	61	58	0	207	107	4	12	0.952	0.981	0.900	NR	DL	CNN	^{18}F-FDG	Glucose metabolism
Wu et al [13]	2022	108	66	42	22	20	0	65	37	1	5	0.945	0.985	0.881	NR	DL	CNN	^{18}F-FDG	Glucose metabolism
Wu et al [13]	2022	108	66	42	22	20	0	65	37	1	5	0.945	0.985	0.881	NR	DL	CNN	^{18}F-FDG	Glucose metabolism
Wu et al [13]	2022	108	66	42	22	20	0	64	41	2	1	0.972	0.969	0.976	NR	DL	CNN	^{18}F-FDG	Glucose metabolism
Wu et al [13]	2022	108	66	42	22	20	0	63	41	3	1	0.963	0.955	0.976	NR	DL	CNN	^{18}F-FDG	Glucose metabolism
Wu et al [13]	2022	547	299	248	150	98	0	286	235	13	13	0.953	0.957	0.948	0.989	DL	CNN	^{18}F-FDG	Glucose metabolism

Wu et al [13]	2022	260	136	124	90	34	0	132	118	4	6	0.962	0.971	0.952	0.989	DL	CNN	^{18}F-FDG	Glucose metabolism
Wu et al [13]	2022	287	163	124	60	64	0	156	117	7	7	0.951	0.957	0.944	0.991	DL	CNN	^{18}F-FDG	Glucose metabolism
Zhao et al [14]	2022	279	146	133	79	54	0	126	123	20	10	0.893	0.863	0.925	0.938	DL	CNN	^{11}C-CFT	Presynaptic dopamine
Zhao et al [14]	2022	140	64	76	53	23	0	58	67	6	9	0.893	0.906	0.882	0.931	DL	CNN	^{11}C-CFT	Presynaptic dopamine
Zhao et al [14]	2022	139	82	57	26	31	0	72	54	10	3	0.906	0.878	0.947	0.950	DL	CNN	^{11}C-CFT	Presynaptic dopamine
Zhao et al [14]	2022	280	194	86	44	42	0	176	76	18	10	0.900	0.907	0.884	NR	DL	CNN	^{11}C-CFT	Presynaptic dopamine
Zhao et al [14]	2022	96	62	34	20	14	0	58	29	4	5	0.906	0.935	0.853	NR	DL	CNN	^{11}C-CFT	Presynaptic dopamine
Zhao et al [14]	2022	96	62	34	20	14	0	56	34	6	0	0.937	0.903	0.999	NR	DL	CNN	^{11}C-CFT	Presynaptic dopamine
Zhao et al [14]	2022	280	194	86	44	42	0	177	77	17	9	0.908	0.912	0.900	NR	DL	CNN	^{11}C-CFT	Presynaptic dopamine
Zhao et al [14]	2022	96	62	34	20	14	0	60	31	2	3	0.948	0.968	0.912	NR	DL	CNN	^{11}C-CFT	Presynaptic dopamine
Zhao et al [14]	2022	96	62	34	20	14	0	57	34	5	0	0.947	0.919	0.999	NR	DL	CNN	11C-CFT	Presynaptic dopamine
Xu et al [15]	2022	107	50	57	37	20	0	42	49	8	8	0.851	0.840	0.860	0.850	ML	SVM	^{11}C-CFT	Presynaptic dopamine
Martins et al [4]	2021	46	27	19	8	0	6CBD;5DLB	27	11	0	8	0.826	1.000	0.579	0.820	ML	SVM	^{11}C-raclopride	Postsynaptic dopamine
Martins et al [4]	2021	46	27	19	8	0	6CBD; 5DLB	21	13	6	6	0.739	0.778	0.684	0.760	ML	SVM	^{11}C-raclopride	Postsynaptic dopamine
Martins et al [4]	2021	46	27	19	8	0	6CBD; 5DLB	24	12	3	7	0.783	0.889	0.632	0.820	ML	SVM	^{11}C-raclopride	Postsynaptic dopamine
Hu et al [16]	2021	63	42	21	21	0	0	40	15	2	6	0.873	0.952	0.714	0.958	ML	LASSO	^{18}F-FDG	Glucose metabolism
Hu et al [16]	2021	63	42	21	21	0	0	41	18	1	3	0.937	0.976	0.857	0.941	ML	LASSO	^{18}F-FDG	Glucose metabolism
Hu et al [16]	2021	63	42	21	21	0	0	38	18	4	3	0.889	0.905	0.857	0.895	ML	LASSO	^{18}F-FDG	Glucose metabolism
Hu et al [16]	2021	63	42	21	21	0	0	41	16	1	5	0.905	0.976	0.762	0.951	ML	LASSO	^{18}F-FDG	Glucose metabolism
Hu et al [16]	2021	63	42	21	21	0	0	40	18	2	3	0.921	0.952	0.857	0.932	ML	LASSO	^{18}F-FDG	Glucose metabolism
Hu et al [16]	2021	63	42	21	21	0	0	38	20	4	1	0.921	0.905	0.952	0.971	ML	LASSO	^{18}F-FDG	Glucose metabolism
Hu et al [16]	2021	63	42	21	21	0	0	39	21	3	0	0.952	0.929	1.000	0.993	ML	LASSO	^{18}F-FDG	Glucose metabolism
Hu et al [16]	2021	27	18	9	9	0	0	16	6	2	3	0.815	0.889	0.667	0.932	ML	LASSO	^{18}F-FDG	Glucose metabolism
Hu et al [16]	2021	27	18	9	9	0	0	14	5	4	5	0.704	0.778	0.500	0.926	ML	LASSO	^{18}F-FDG	Glucose metabolism
Hu et al [16]	2021	27	18	9	9	0	0	15	9	3	0	0.889	0.833	1.000	0.889	ML	LASSO	^{18}F-FDG	Glucose metabolism

Hu et al [16]	2021	27	18	9	9	0	0	15	5	3	4	0.741	0.833	0.556	0.951	ML	LASSO	^{18}F-FDG	Glucose metabolism
Hu et al [16]	2021	27	18	9	9	0	0	13	5	5	5	0.667	0.722	0.500	0.920	ML	LASSO	^{18}F-FDG	Glucose metabolism
Hu et al [16]	2021	27	18	9	9	0	0	15	9	3	0	0.889	0.833	1.000	0.957	ML	LASSO	^{18}F-FDG	Glucose metabolism
Hu et al [16]	2021	27	18	9	9	0	0	17	9	1	0	0.963	0.944	1.000	0.994	ML	LASSO	^{18}F-FDG	Glucose metabolism
Choi et al [17]	2021	26	19	7	0	0	7VP	19	5	0	2	0.923	1.000	0.714	0.857	DL	CNN	^{18}F-FP-CIT	Presynaptic dopamine
Rus et al [18]	2020	56	43	13	6	7	0	37	12	6	1	0.875	0.860	0.923	NR	ML	LR	^{18}F-FDG	Glucose metabolism
Rus et al [18]	2020	43	22	21	11	10	0	18	20	4	1	0.884	0.818	0.952	NR	ML	LR	^{18}F-FDG	Glucose metabolism
Segovia et al [19]	2017	87	39	48	24	24	0	29	37	10	11	0.759	0.744	0.771	NR	ML	SVM	^{18}F-DMFP	Postsynaptic dopamine
Segovia et al [19]	2017	87	39	48	24	24	0	26	37	13	11	0.724	0.667	0.771	NR	ML	SVM	^{18}F-DMFP	Postsynaptic dopamine
Segovia et al [19]	2017	87	39	48	24	24	0	22	35	17	13	0.655	0.564	0.729	NR	ML	SVM	^{18}F-DMFP	Postsynaptic dopamine
Segovia et al [19]	2017	87	39	48	24	24	0	31	27	8	21	0.667	0.795	0.563	0.760	ML	SVM	^{18}F-DMFP	Postsynaptic dopamine
Segovia et al [19]	2017	87	39	48	24	24	0	29	28	10	20	0.655	0.744	0.583	0.717	ML	SVM	^{18}F-DMFP	Postsynaptic dopamine
Segovia et al [20]	2017	87	39	48	24	24	0	30	33	9	15	0.724	0.769	0.688	0.770	ML	SVM	^{18}F-DMFP	Postsynaptic dopamine
Segovia et al [21]	2015	87	39	48	24	24	0	24	37	15	11	0.701	0.615	0.771	NR	ML	SVM	^{18}F-DMFP	Postsynaptic dopamine
Segovia et al [21]	2015	87	39	48	24	24	0	27	37	12	11	0.736	0.692	0.771	NR	ML	SVM	^{18}F-DMFP	Postsynaptic dopamine
Segovia et al [21]	2015	87	39	48	24	24	0	26	35	13	13	0.701	0.667	0.729	NR	ML	SVM	^{18}F-DMFP	Postsynaptic dopamine
Segovia et al [21]	2015	87	39	48	24	24	0	29	36	10	12	0.747	0.744	0.750	NR	ML	SVM	^{18}F-DMFP	Postsynaptic dopamine
Segovia et al [21]	2015	87	39	48	24	24	0	28	38	11	10	0.759	0.718	0.792	NR	ML	SVM	^{18}F-DMFP	Postsynaptic dopamine
Segovia et al [21]	2015	87	39	48	24	24	0	30	38	9	10	0.782	0.769	0.792	NR	ML	SVM	^{18}F-DMFP	Postsynaptic dopamine
Garraux et al [22]	2013	120	42	78	31	26	21CBS	39	65	3	13	0.867	0.929	0.833	NR	ML	DT	^{18}F-FDG	Glucose metabolism
Tang et al [23]	2010	167	96	71	41	30	0	81	69	15	2	0.898	0.844	0.972	0.970	ML	LR	^{18}F-FDG	Glucose metabolism
Tang et al [23]	2010	55	30	25	11	14	0	23	23	7	2	0.836	0.767	0.920	NR	ML	LR	^{18}F-FDG	Glucose metabolism
Tang et al [23]	2010	112	66	46	30	16	0	58	46	8	0	0.929	0.879	1.000	NR	ML	LR	^{18}F-FDG	Glucose metabolism
Tang et al [23]	2010	22	9	13	5	8	0	6	12	3	1	0.818	0.667	0.923	NR	ML	LR	^{18}F-FDG	Glucose metabolism
Tang et al [23]	2010	33	21	12	6	6	0	17	11	4	1	0.848	0.810	0.917	NR	ML	LR	^{18}F-FDG	Glucose metabolism

Tang et al [23]	2010	28	8	20	11	9	0	4	20	4	0	0.857	0.500	1.000	NR	ML	LR	^{18}F-FDG	Glucose metabolism
Tang et al [23]	2010	84	58	26	19	7	0	54	26	4	0	0.952	0.931	1.000	NR	ML	LR	^{18}F-FDG	Glucose metabolism

SE Sensitivity, SP Specificity, NR not reported, AI artificial intelligence, DL deep learning, ML machine learning, CBS corticobasal syndrome, MSA multiple system atrophy, PSP progressive supranuclear palsy, CBD corticobasal degeneration, DLB dementia with Lewy bodies.

Supplementary Table 14. Description of quality assessment based on QUADAS-AI domains

Domain	Subject selection	Index text (AI)	Reference standard	Work-flow
Concern	Signaling question:<br>• Q1: Accurately characterize the source, size and quality of input data alongside clear patient eligibility criteria?<br>• Q2: Was it derived from open-source datasets?<br>• Q3: Present the rationale and breakdown of its training, validation and test sets?<br>• Q4: Whether to perform image pre-processing?<br>• Q5: Provide the scanner model information used to acquire imaging data?	Signaling question:<br>• Q6: Was external verification performed?	Signaling question:<br>• Q7: Was the reference standard likely to correctly classify the target condition?	Signaling question:<br>• Q8: Was the time between the index test and the reference standard reasonable?
Concerns regarding “risk of bias”	Risk of bias is judged as “low”, “high”, or “unclear”. If all signaling questions for a domain are answered “yes” then risk of bias can be judged “low”.<br>• If any signaling question is answered “no” this flags the potential for bias. Review authors then need to have in-depth discussions to judge risk of bias.<br>• The “unclear” category should be used only when insufficient data are reported to permit a judgment.			

Supplementary Figure 1. Forest plot using presynaptic DA PET imaging (PD vs. NC)

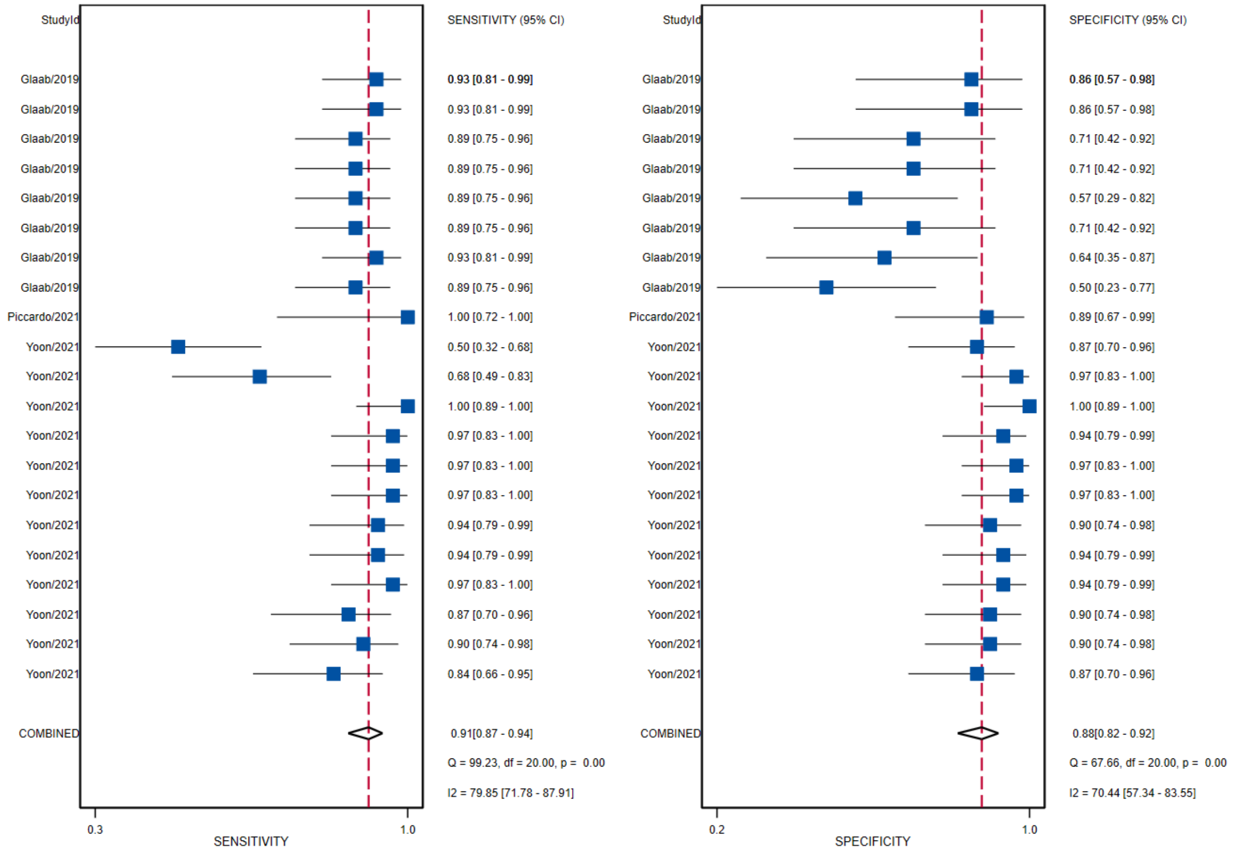

Supplementary Figure 2. Forest plot with highest performance using ^{18}F-FDG PET imaging (PD vs. NC)

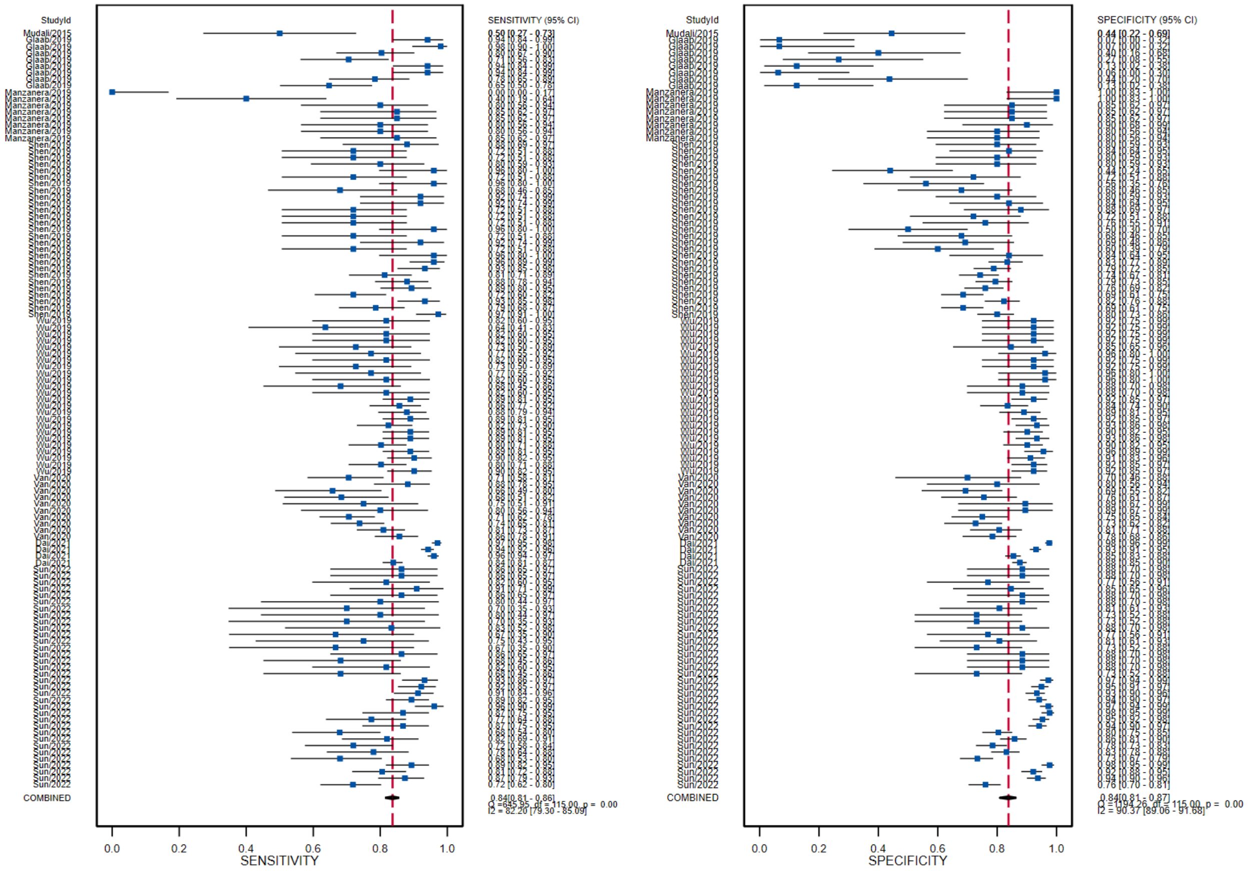

Supplementary Figure 3. Forest plot using ^{18}F-FDG PET imaging (PD vs. NC)

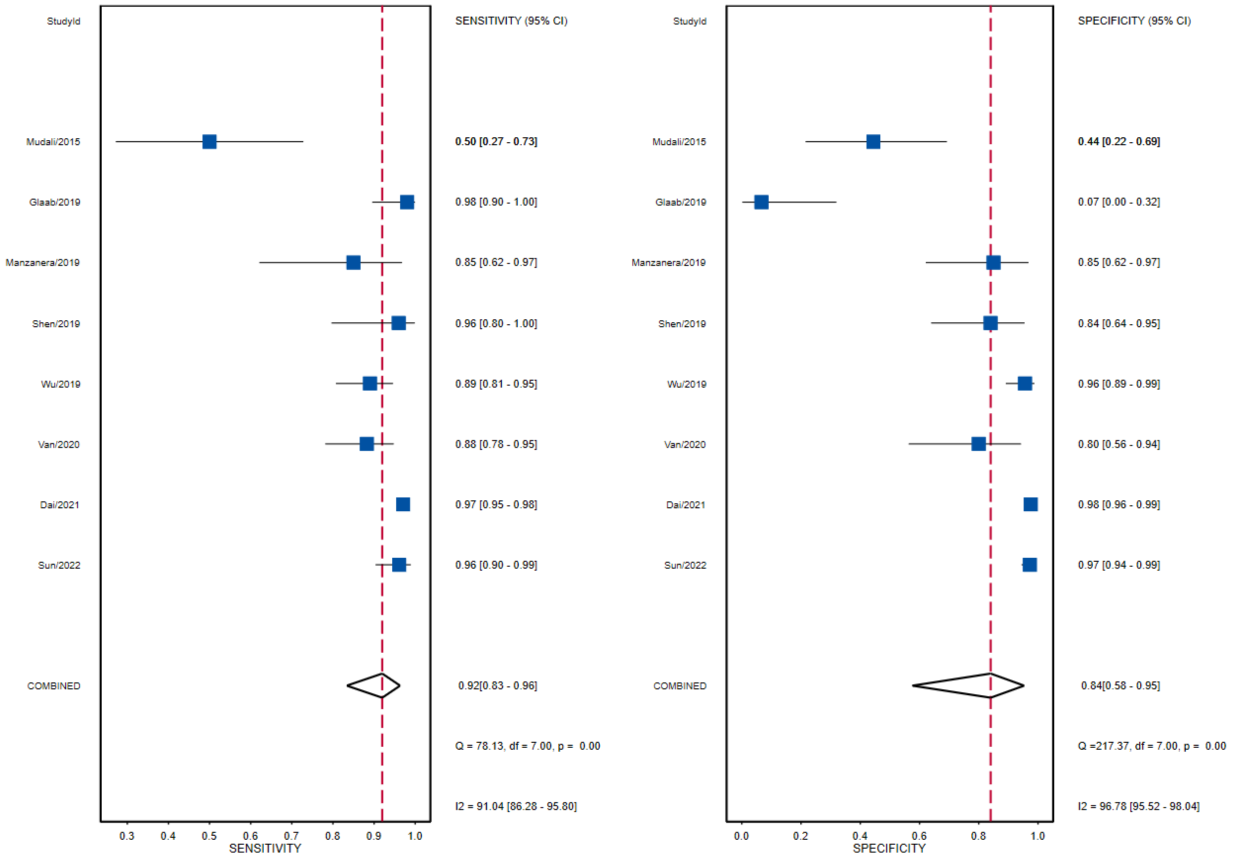

Note. 8 tables with highest performance.

Supplementary Figure 4. Forest plot using DL- and ML-assisted ^{18}F-FDG PET imaging (PD vs. NC)

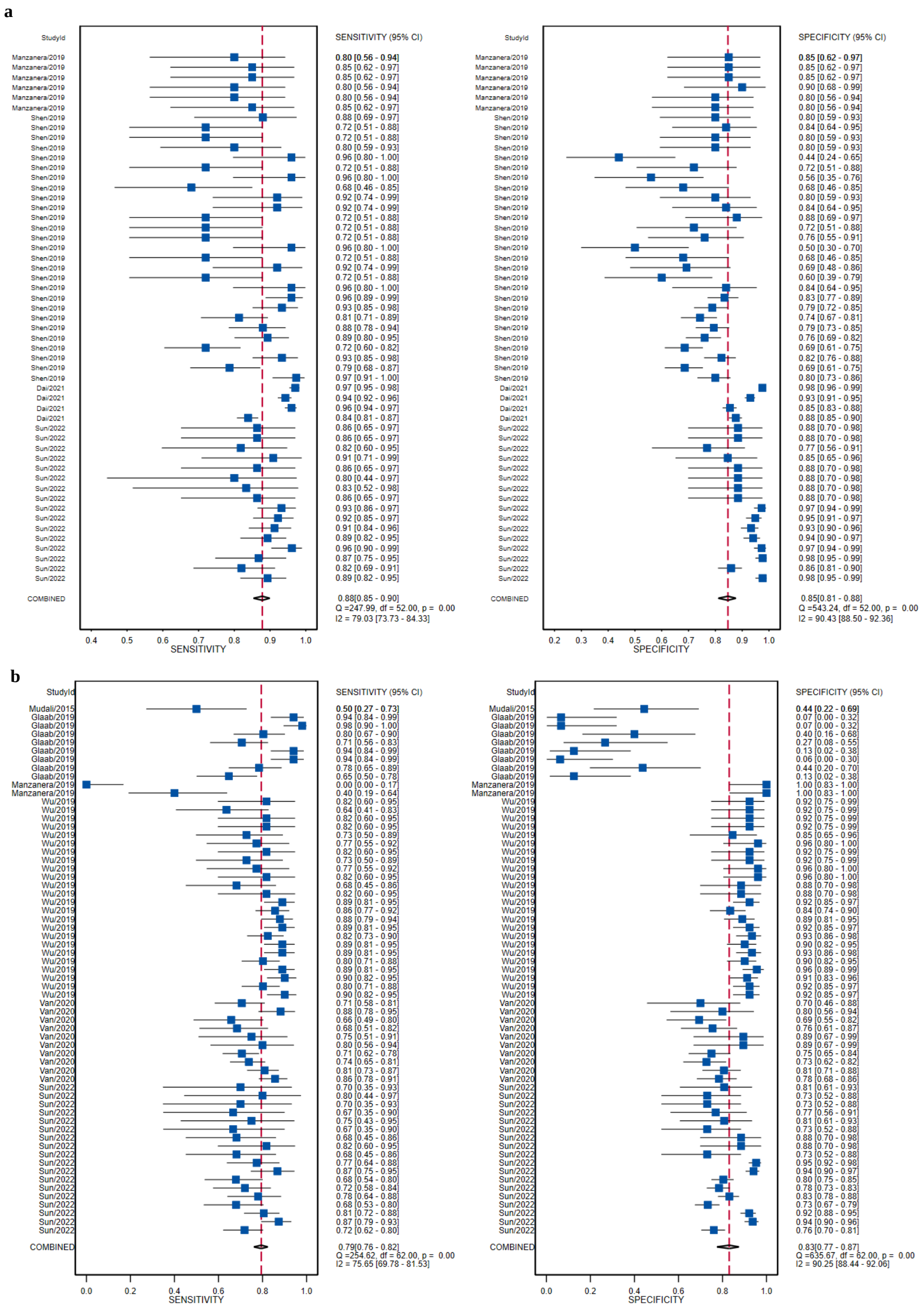

a. DL (4 studies with 53 contingency tables).

b. ML (6 studies with 63 contingency tables).

Supplementary Figure 5. Forest plot using SVM-assisted ^{18}F-FDG PET imaging (PD vs. NC)

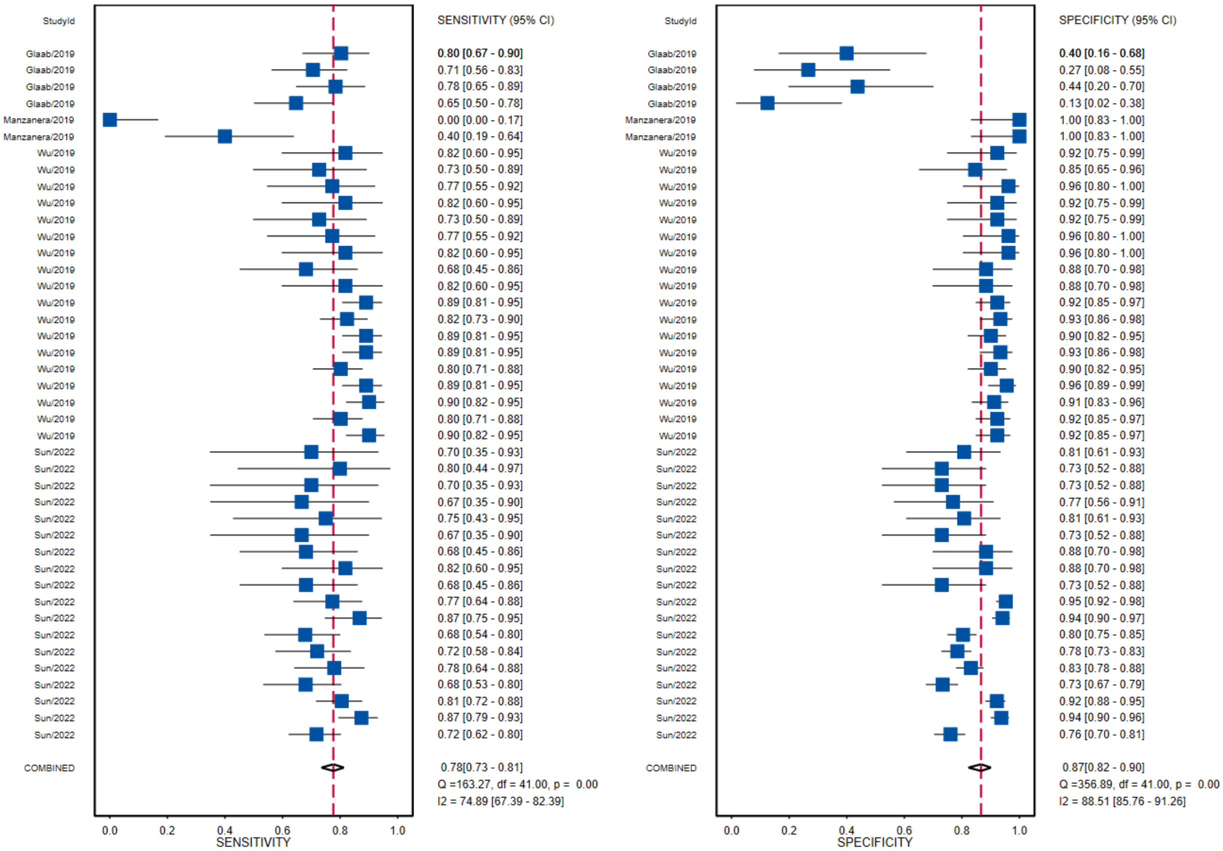

Note. 4 studies with 42 contingency tables.

Supplementary Figure 6. Forest plot using additional ML-assisted ^{18}F-FDG PET imaging (PD vs. NC)

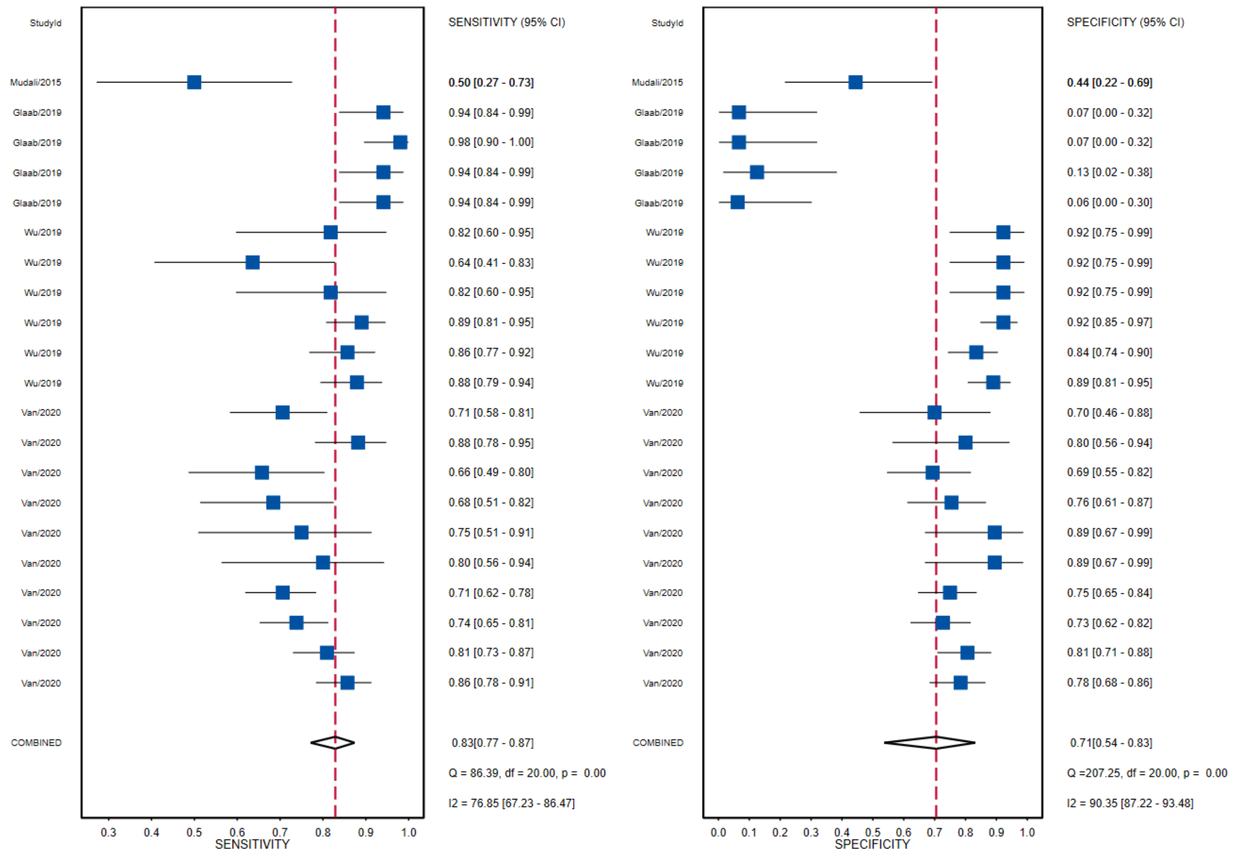

Note. 4 studies with 21 contingency tables. Additional ML algorithms includes RF, LVQ, DT.

Supplementary Figure 7. Forest plot using ^{18}F-FDG PET imaging based on sample size (PD vs. NC)

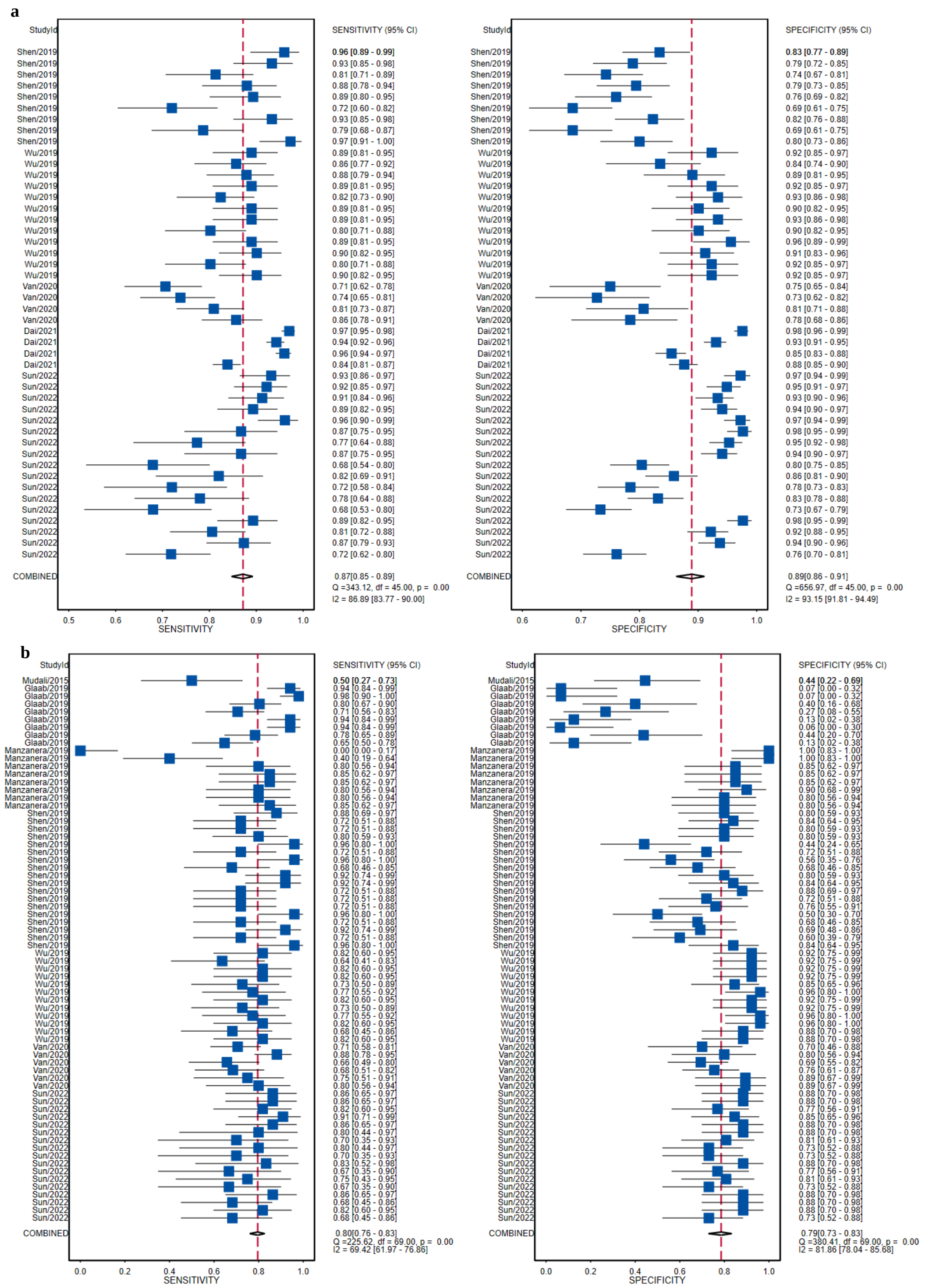

a: sample size ≥ 100 (5 studies with 46 tables).

b: sample size < 100 (7 studies with 70 tables).

Supplementary Figure 8. Forest plot using presynaptic DA PET imaging (PD vs. AP)

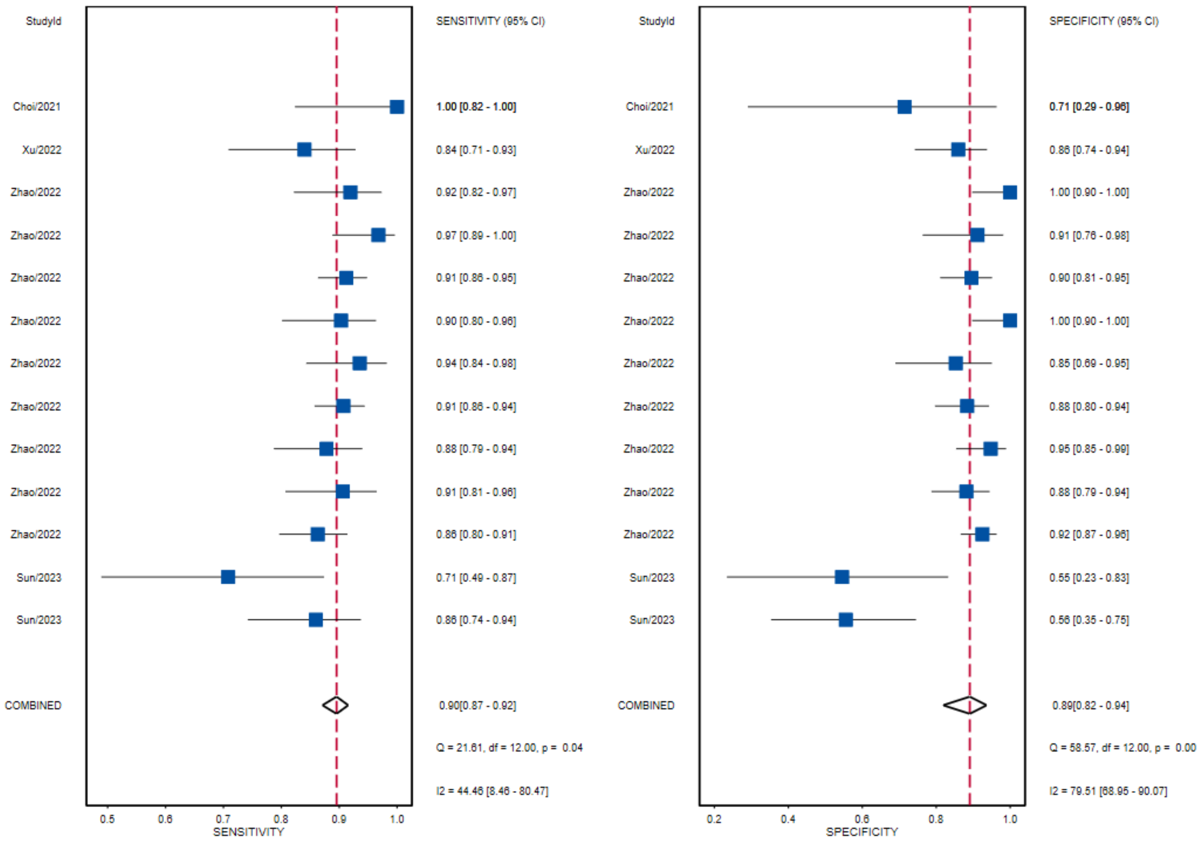

Note. 4 studies with 13 contingency tables.

Supplementary Figure 9. Forest plot with highest performance using presynaptic DA PET imaging (PD vs. AP)

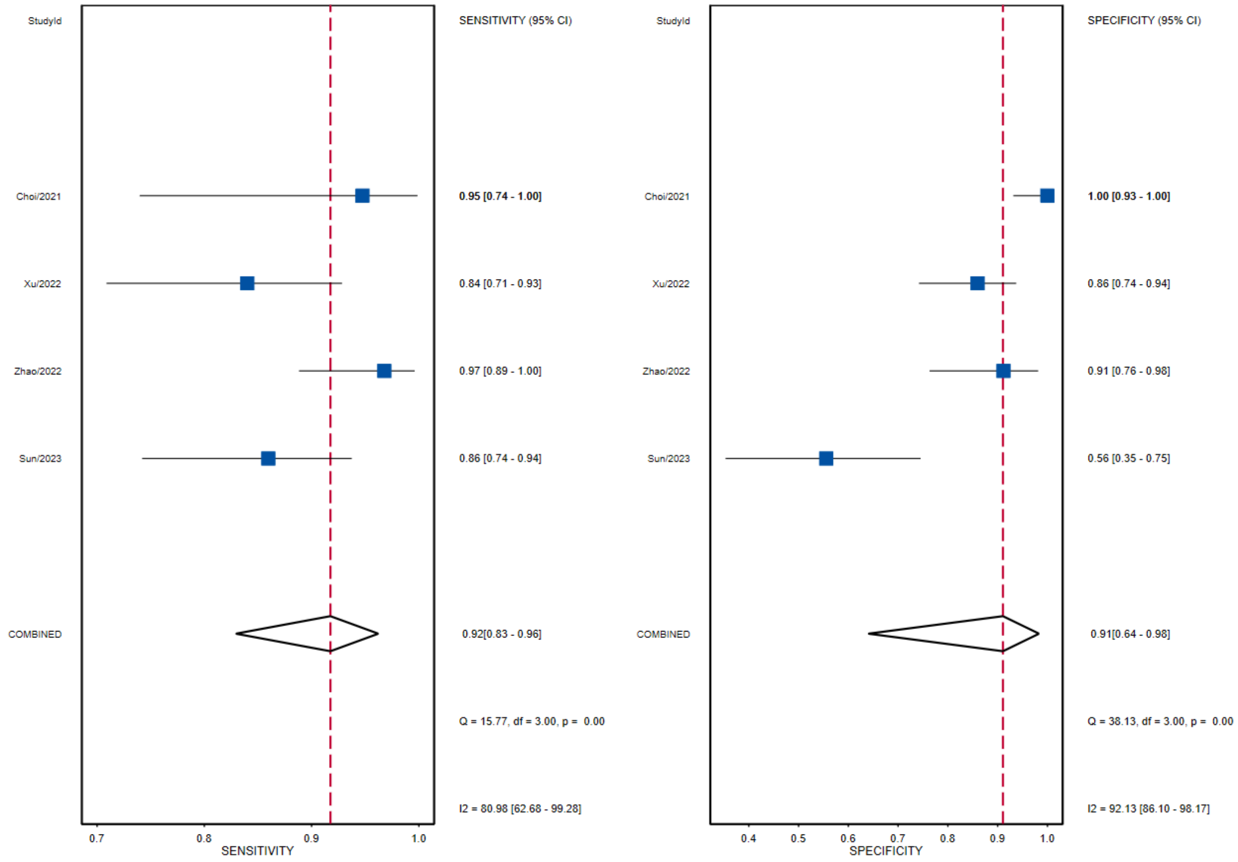

Note. 4 tables with highest performance.

Supplementary Figure 10. Forest plot using ^{11}C CFT PET imaging (PD vs. AP)

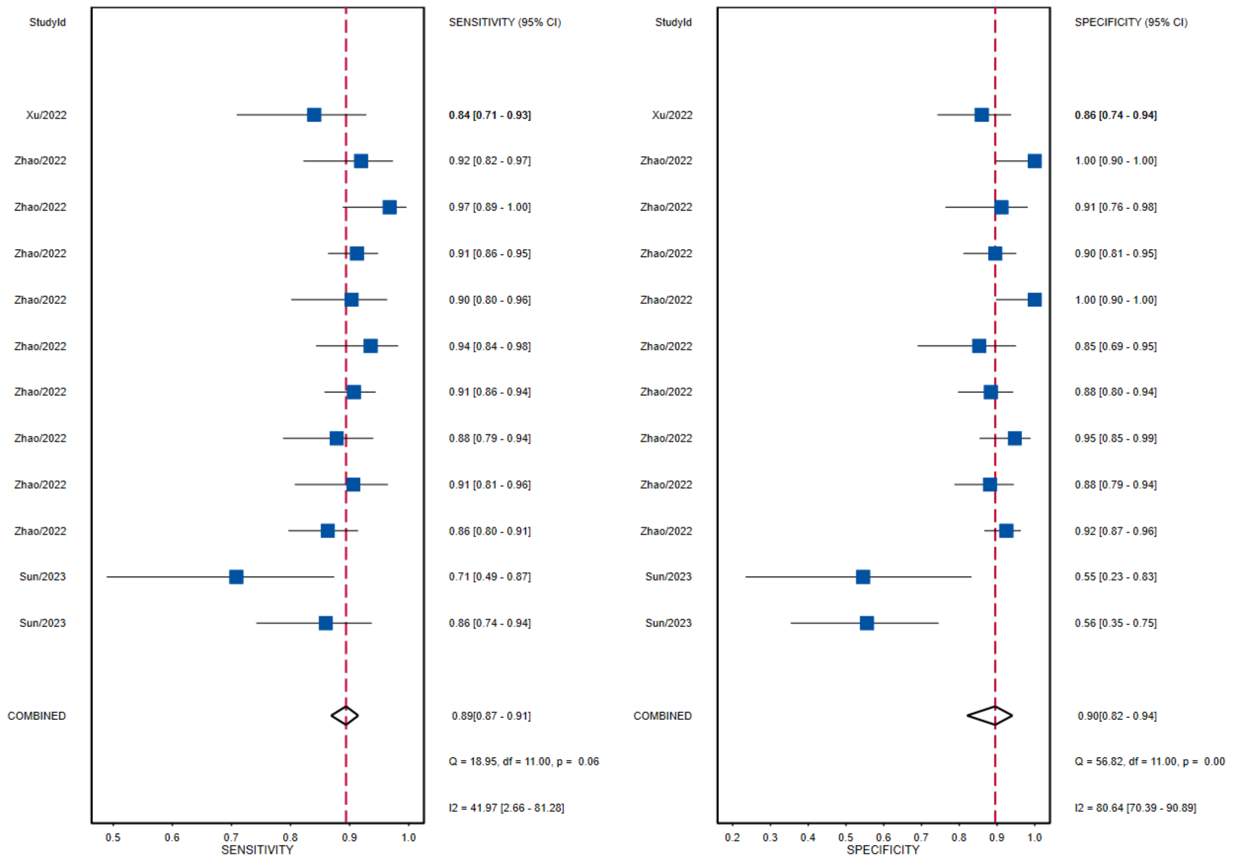

Note. 3 studies with 12 contingency tables.

Supplementary Figure 11. Forest plot using DL-assisted presynaptic DA PET imaging (PD vs. AP)

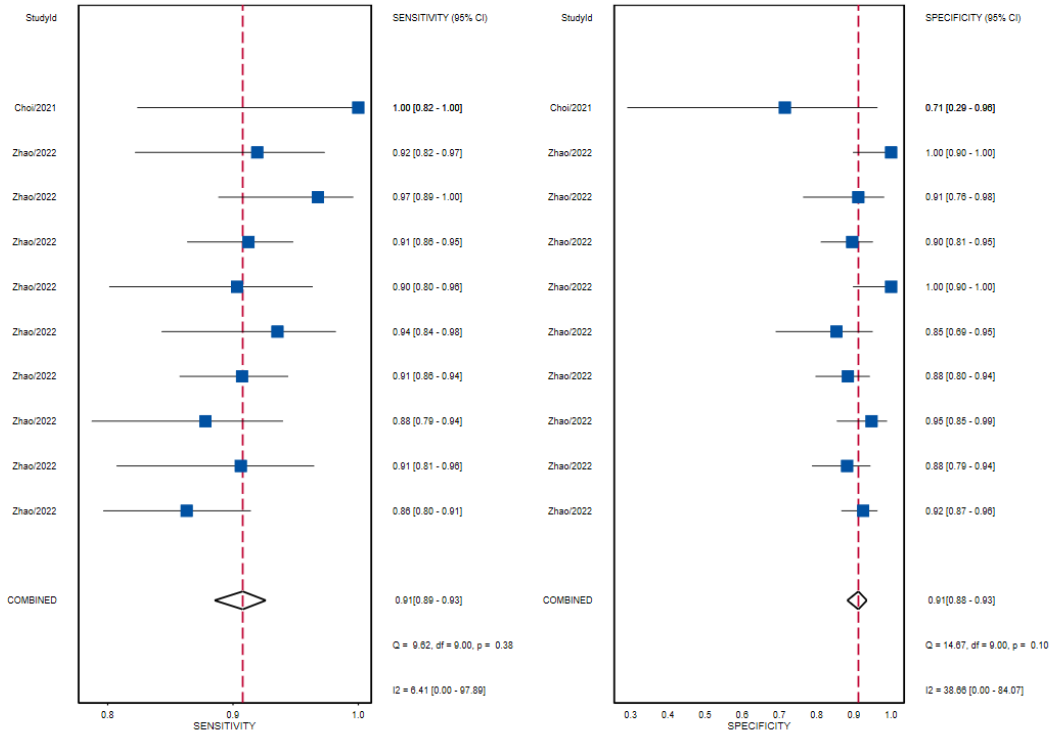

Note. 2 studies with 10 contingency tables.

Supplementary Figure 12. Forest plot using postsynaptic DA PET imaging (PD vs. AP)

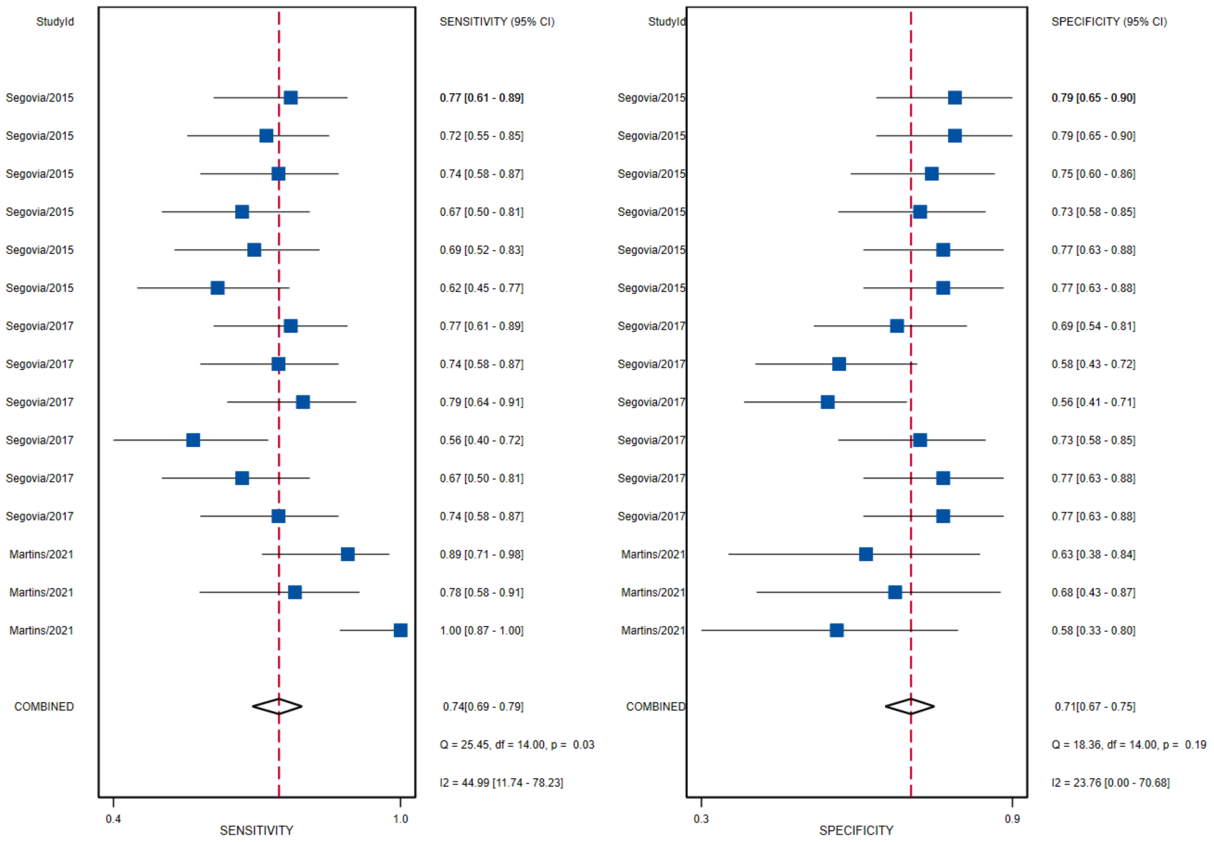

Note. 4 studies with 15 contingency tables.

Supplementary Figure 13. Forest plot with highest performance using postsynaptic DA PET imaging (PD vs. AP)

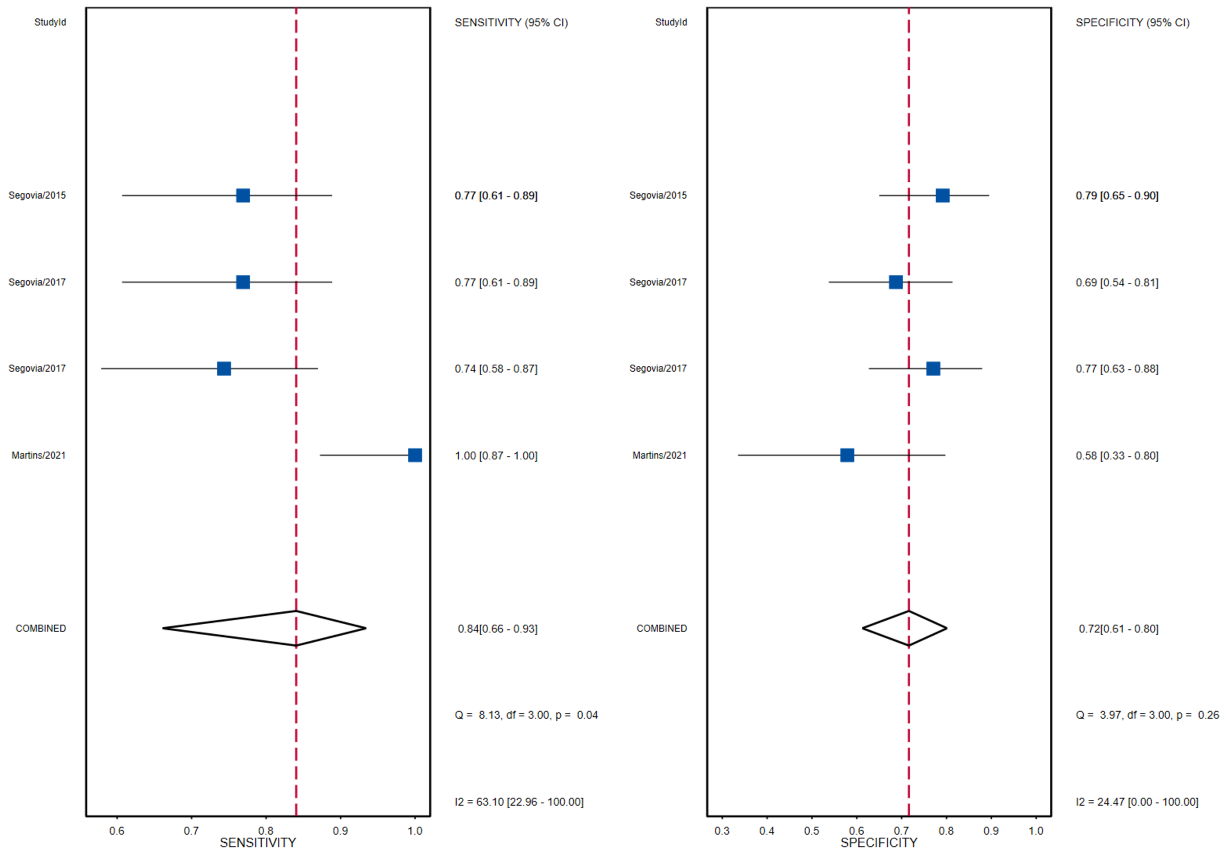

Note. 4 tables with highest performance.

Supplementary Figure 14. Forest plot using ¹⁸F-FDG PET imaging (PD vs. AP)

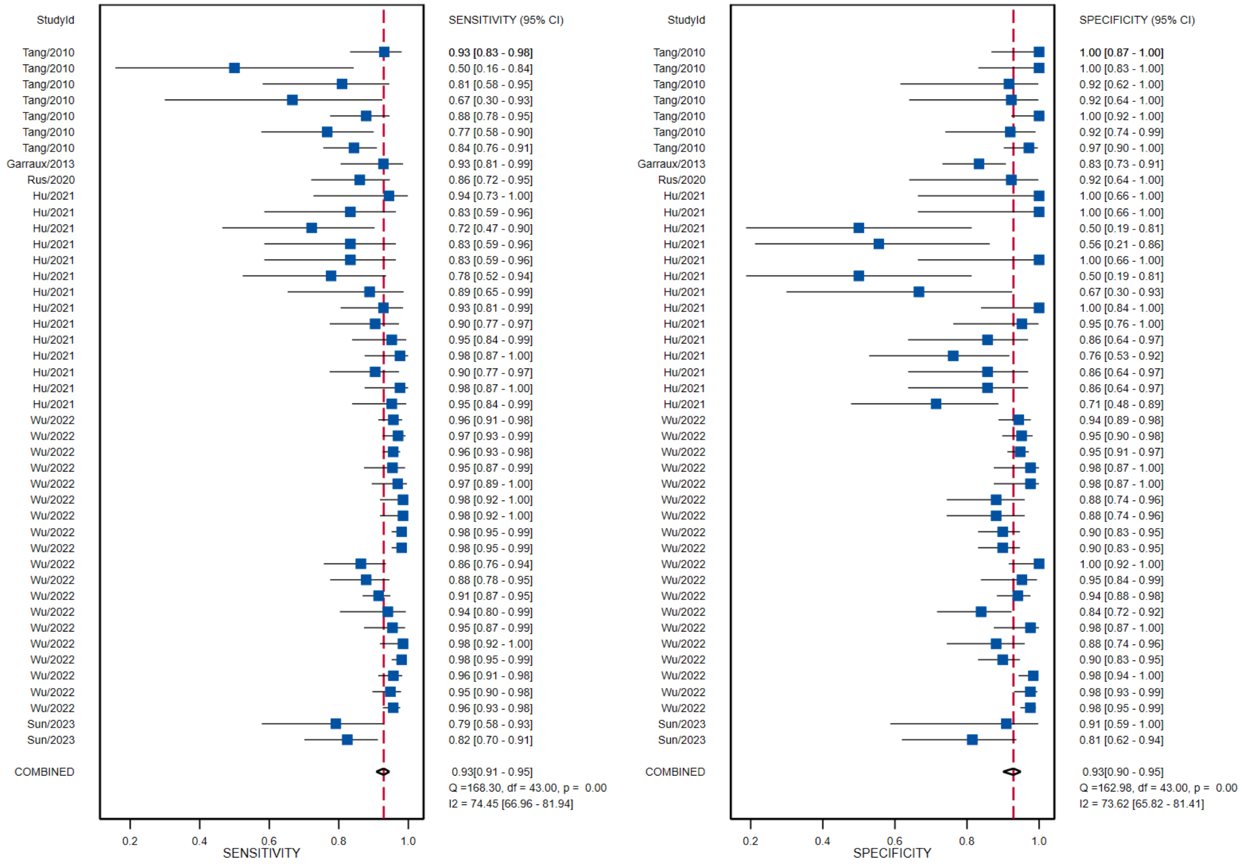

Note. 6 studies with 45 contingency tables.

Supplementary Figure 15. Forest plot with highest performance using ^{18}F-FDG PET imaging (PD vs. AP)

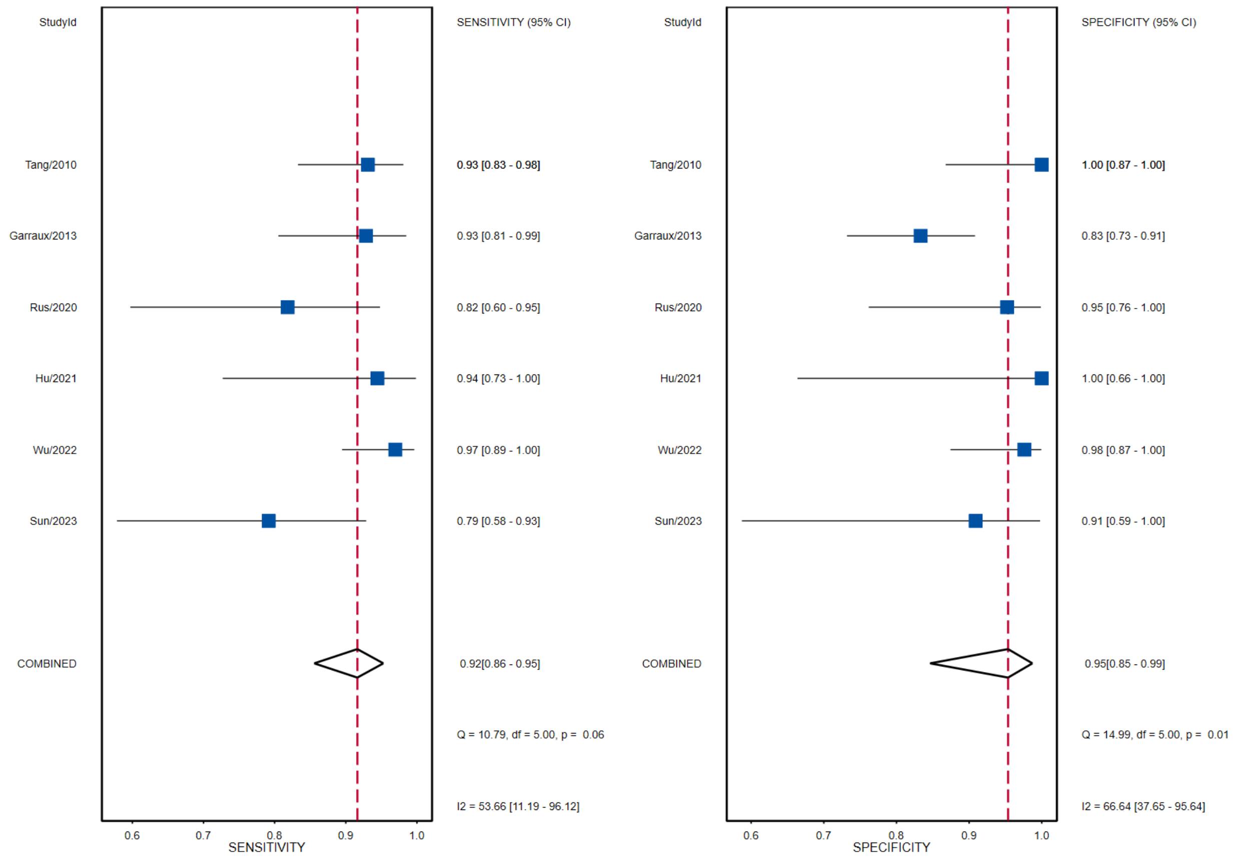

Note. 6 tables with highest performance.

Supplementary Figure 16. Forest plot using DL- and ML-assisted ^{18}F-FDG PET imaging (PD vs. AP)

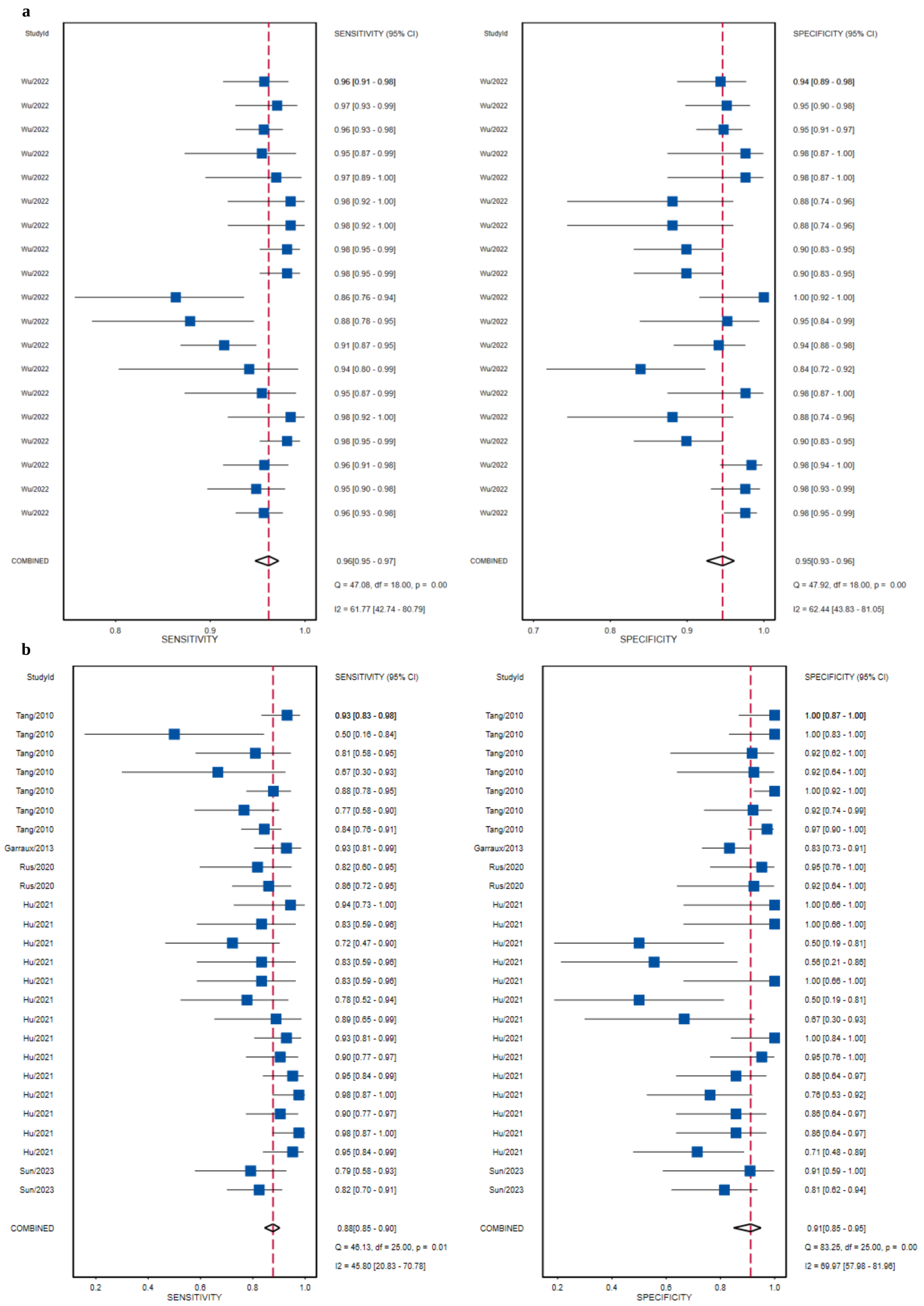

a. DL (1 studies with 19 contingency tables).

b. ML (5 studies with 25 contingency tables).

Supplementary Figure 17. Forest plot using LR-assisted ^{18}F-FDG PET imaging (PD vs. AP)

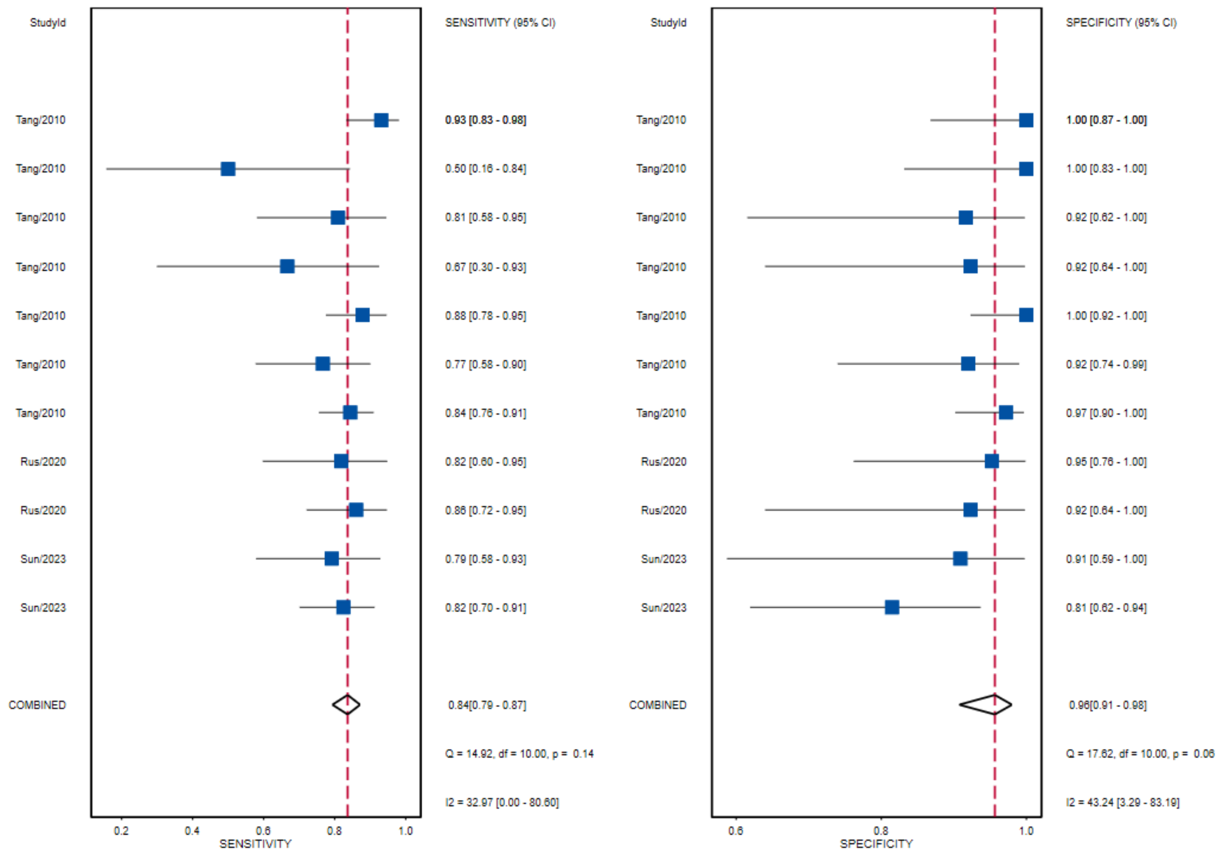

Note. 3 studies with 11 contingency tables.

Supplementary Figure 18. Forest plot using ^{18}F-FDG PET imaging based on sample size (PD vs. AP)

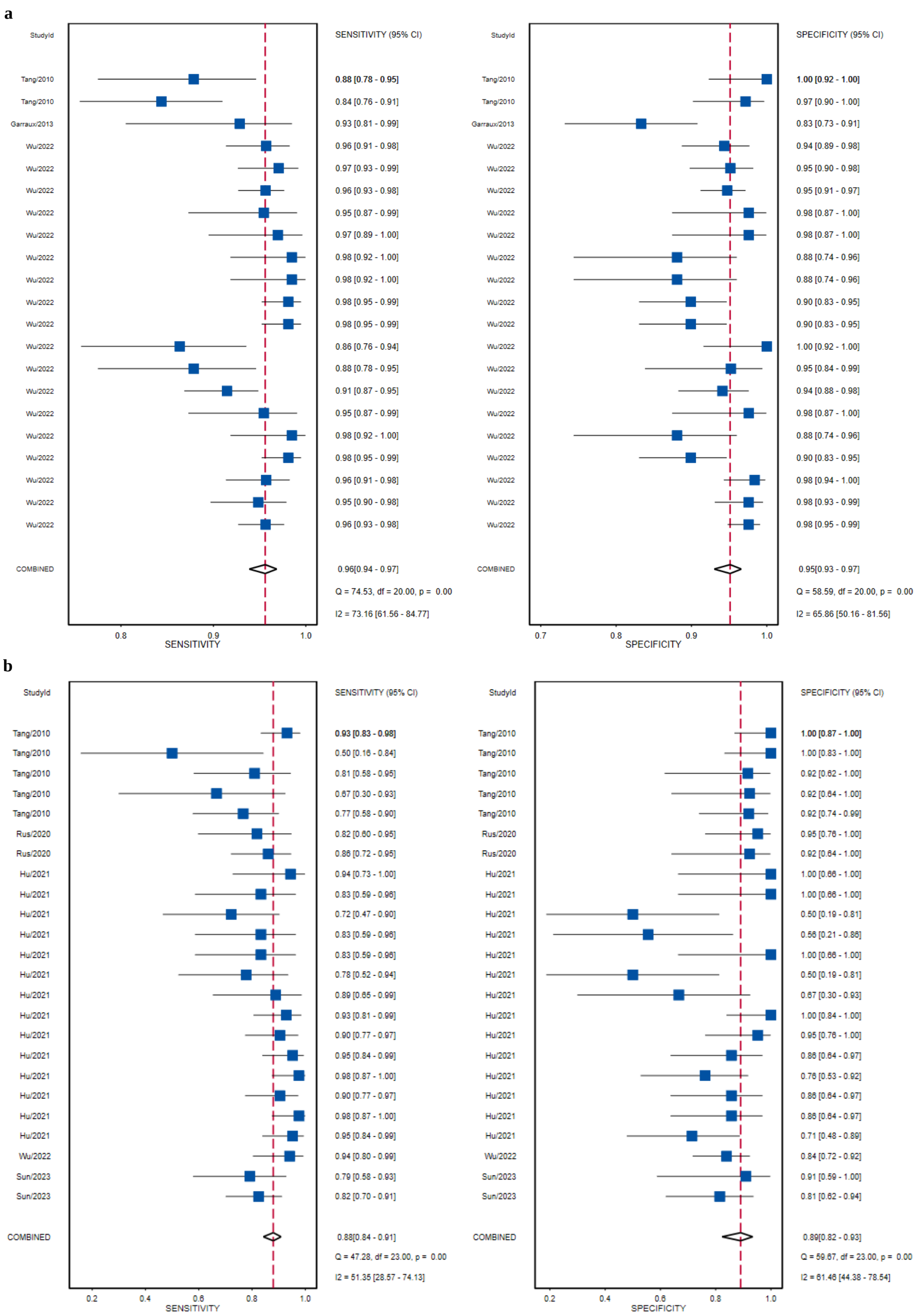

a. sample ≥ 100 (3 studies with 21 tables).

b. sample < 100 (5 studies with 24 tables).

Supplementary Figure 19. SROC curves using DL- and ML-assisted ^{18}F-FDG PET imaging (PD vs. NC)

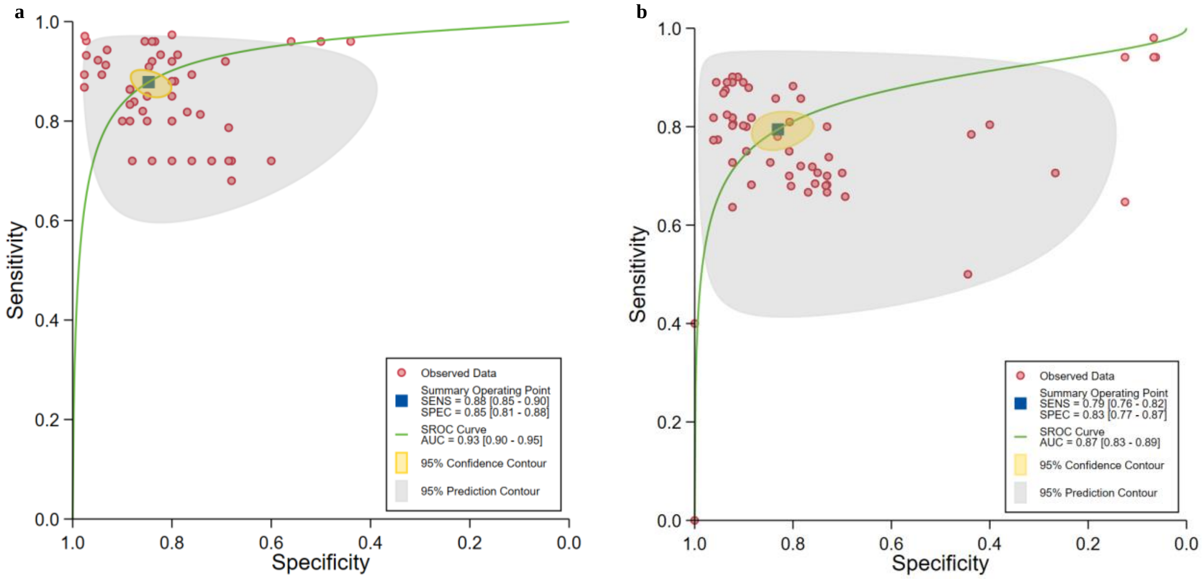

a. DL (4 studies with 53 contingency tables).

b. ML (6 studies with 63 contingency tables).

Supplementary Figure 20. SROC curves using SVM-assisted ^{18}F-FDG PET imaging (PD vs. NC)

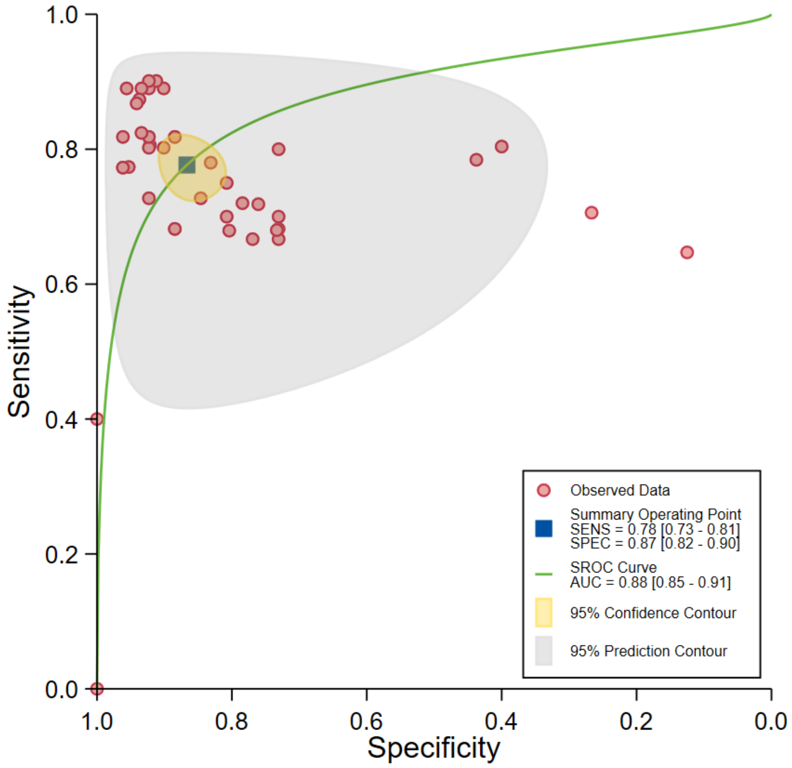

Note. 4 studies with 42 contingency tables.

Supplementary Figure 21. SROC curves using additional ML-assisted ^{18}F-FDG PET imaging (PD vs. NC)

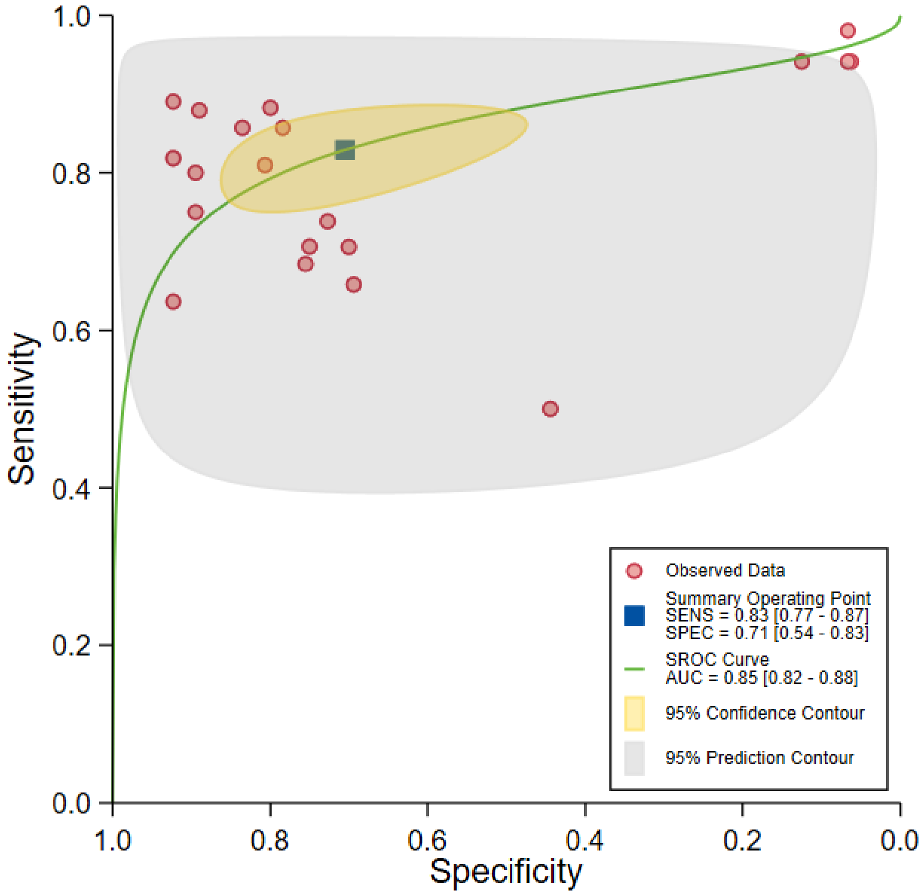

Note. 4 studies with 21 contingency tables. Additional ML algorithms includes RF, LVQ, DT.

Supplementary Figure 22. SROC curves using ^{18}F-FDG PET imaging based on sample size (PD vs. NC)

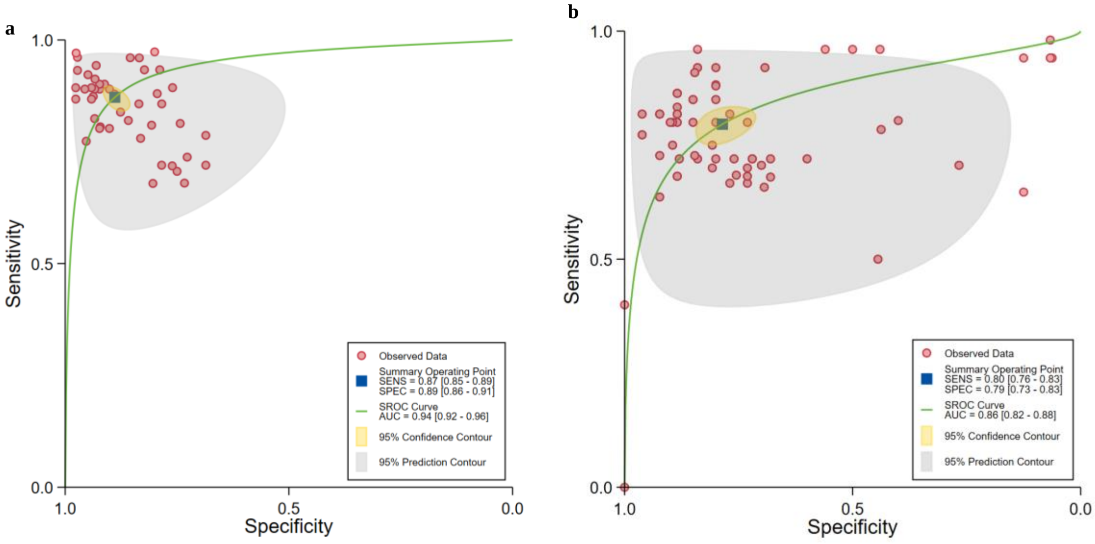

a. Sample size ≥ 100 (5 studies with 46 tables).

b. Sample size < 100 (7 studies with 70 tables).

Supplementary Figure 23. SROC curves using DL- and ML-assisted ^{18}F-FDG PET imaging (PD vs. AP)

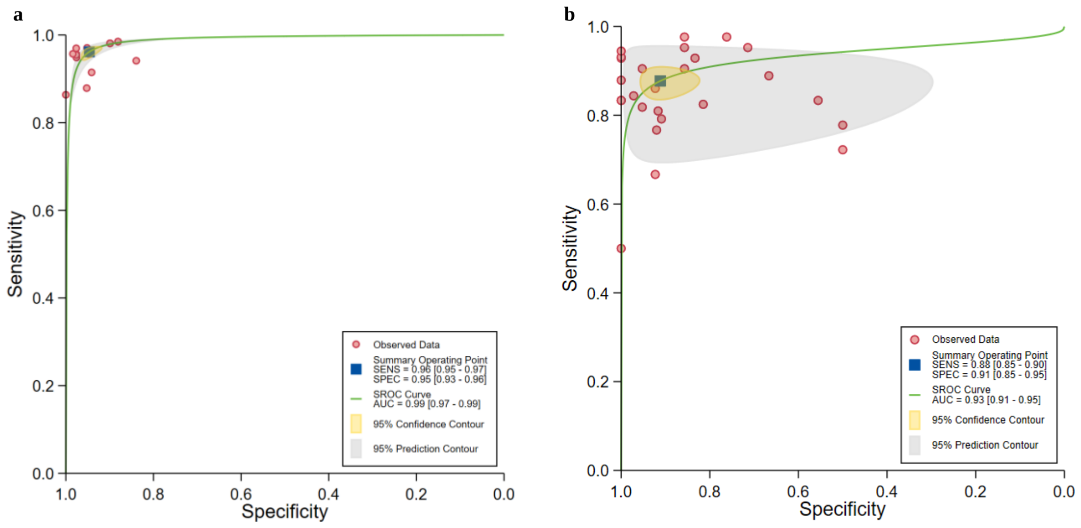

a. DL (1 studies with 19 contingency tables).

b. ML (5 studies with 26 contingency tables).

Supplementary Figure 24. SROC curves using ^{18}F-FDG PET imaging based on sample size (PD vs. AP)

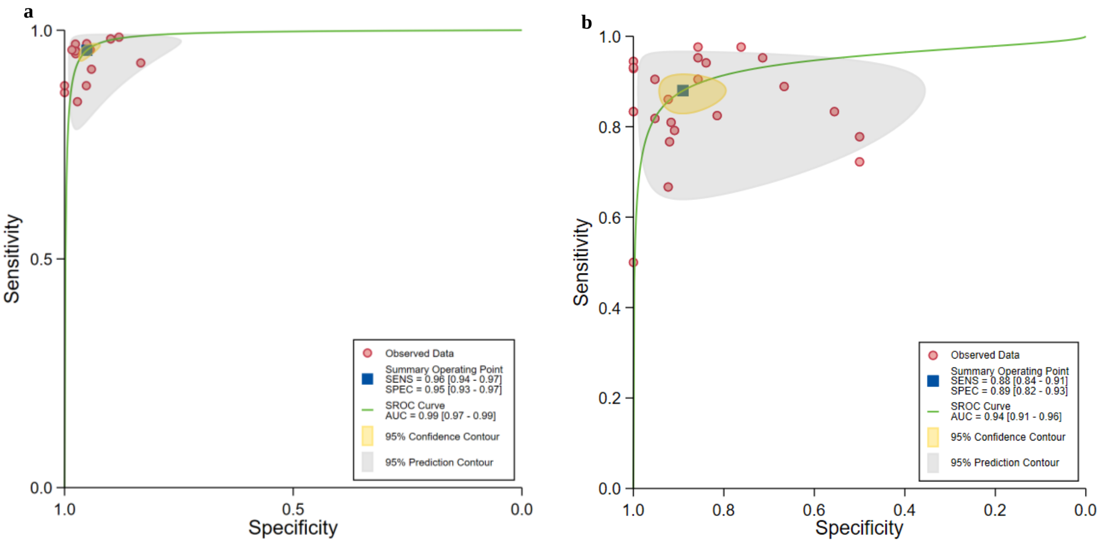

a. sample size ≥ 100 (3 studies with 21 tables).

b. sample size < 100 (5 studies with 24 tables).

Supplementary Figure 25. Publication bias using presynaptic DA and ^{18}F-FDG PET imaging (PD vs. NC)

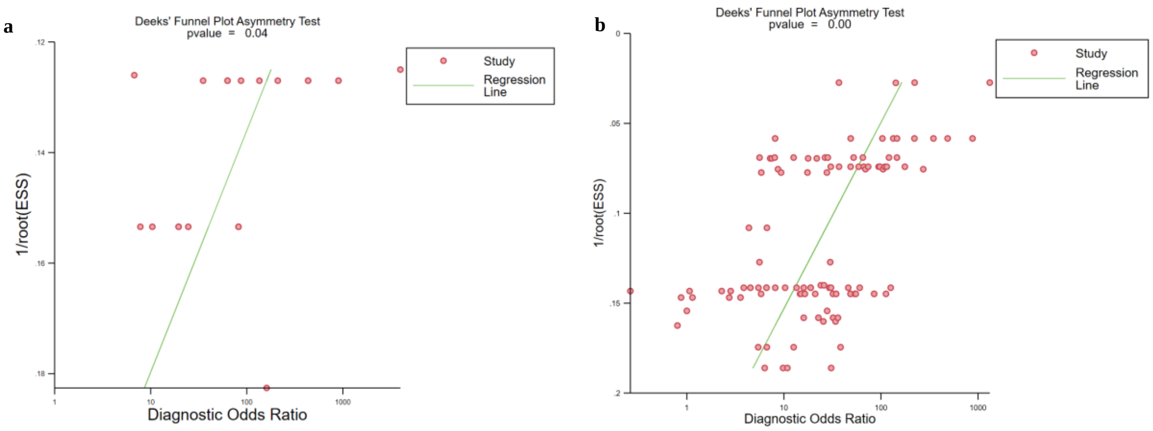

a. Using presynaptic DA PET imaging (Funnel plots suggested there was a publication bias).

b. Using ^{18}F-FDG PET imaging (Funnel plots suggested there was a publication bias).

Supplementary Figure 26. Publication bias using DL- and ML- assisted ^{18}F-FDG PET imaging (PD vs. NC)

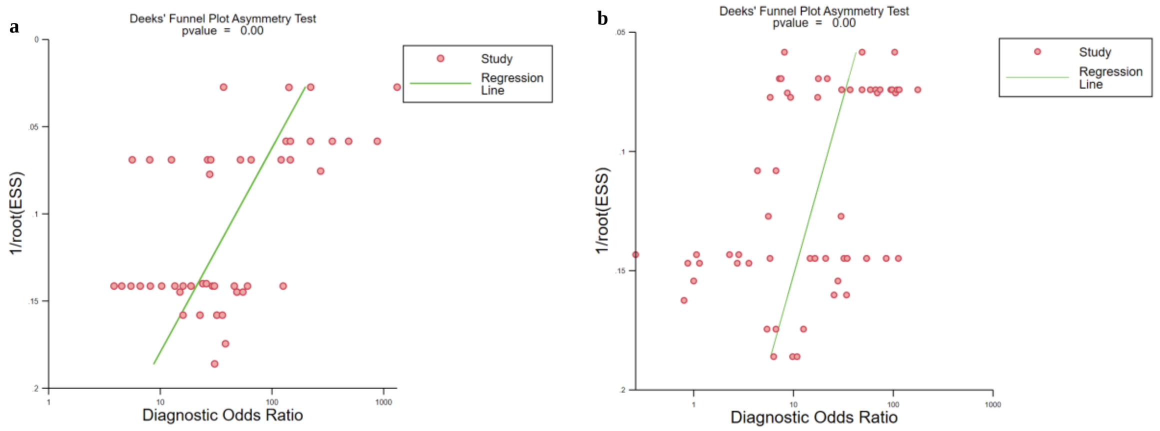

a. Using DL- assisted ^{18}F-FDG PET imaging (Funnel plots suggested there was a publication bias).

b. Using ML-assisted ^{18}F-FDG PET imaging (Funnel plots suggested there was a publication bias).

Supplementary Figure 27. Publication bias using ML- assisted ^{18}F-FDG PET imaging (PD vs. NC)

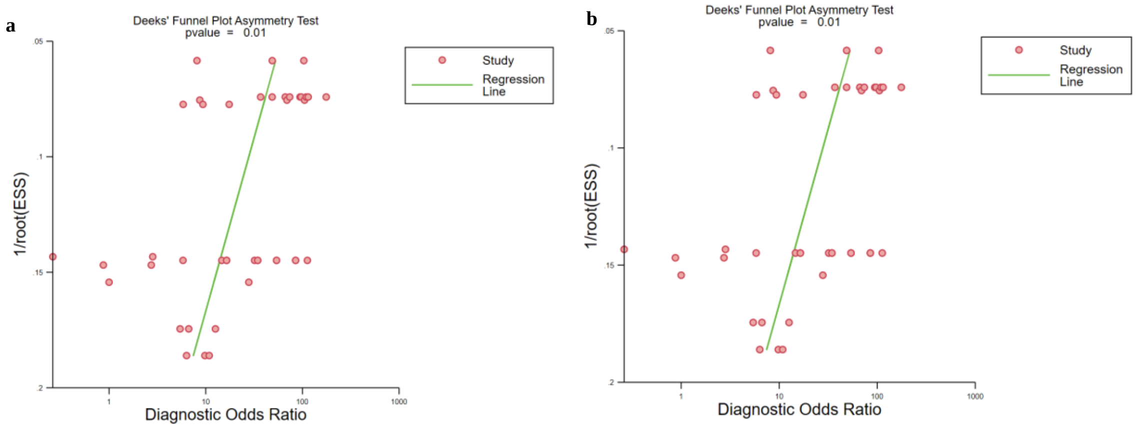

a. Using SVM-assisted ^{18}F-FDG PET imaging (Funnel plots suggested there was a publication bias).

b. Using additional ML-assisted ^{18}F-FDG PET imaging (Funnel plots suggested there was a publication bias).

Supplementary Figure 28. Publication bias using ^{18}F-FDG PET imaging based on sample size (PD vs. NC)

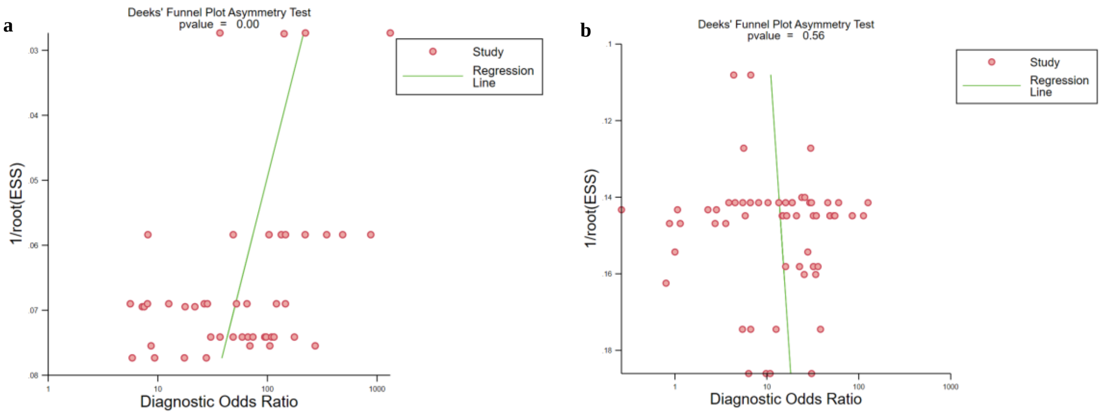

a. Sample size ≥ 100 (Funnel plots suggested there was a publication bias).

b. Sample size < 100 (Funnel plots suggested there was no publication bias).

Supplementary Figure 29. Publication bias using presynaptic DA PET imaging (PD vs. AP)

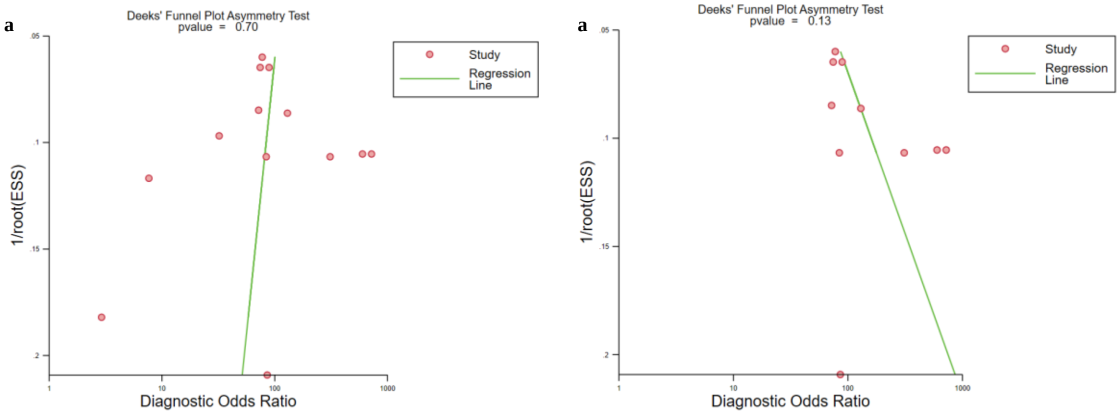

a. Using presynaptic DA PET imaging (Funnel plots suggested there was no publication bias).

b. Using DL-assisted presynaptic DA PET imaging (Funnel plots suggested there was no publication bias).

Supplementary Figure 30. Publication bias using postsynaptic DA and ^{18}F-FDG PET imaging (PD vs. AP)

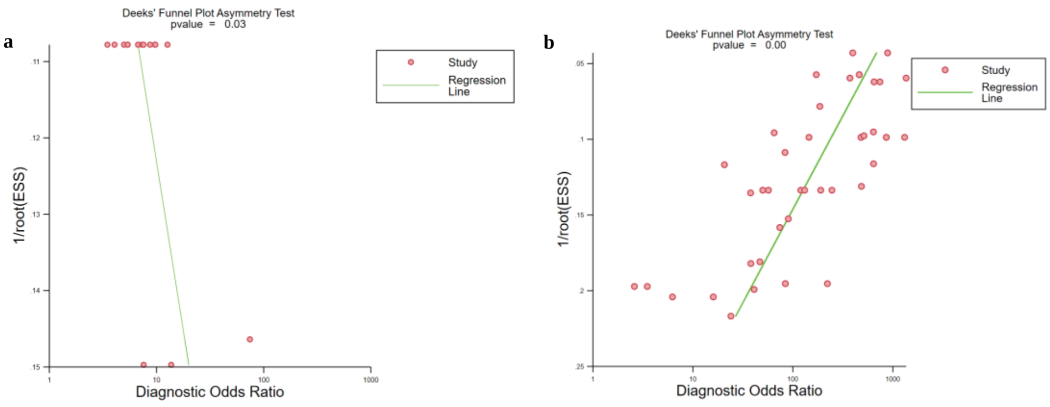

a. Using postsynaptic DA PET imaging (Funnel plots suggested there was a publication bias).

b. Using ^{18}F-FDG PET imaging (Funnel plots suggested there was a publication bias).

Supplementary Figure 31. Publication bias using DL- and ML- assisted ^{18}F-FDG PET imaging (PD vs. AP)

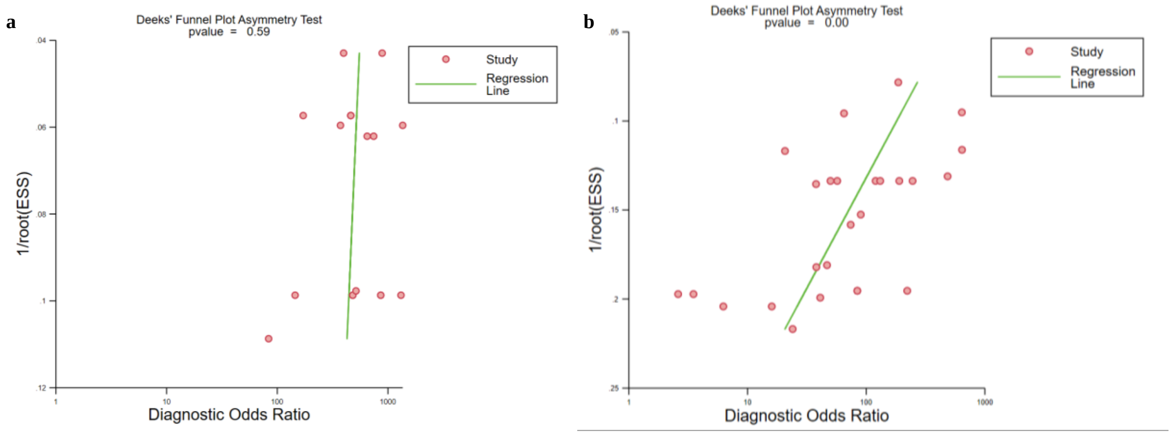

a. Using DL-assisted ^{18}F-FDG PET imaging (Funnel plots suggested there was no publication bias).

b. Using ML-assisted ^{18}F-FDG PET imaging (Funnel plots suggested there was a publication bias).

Supplementary Figure 32. Publication bias using LR-assisted ^{18}F-FDG PET imaging (PD vs. AP)

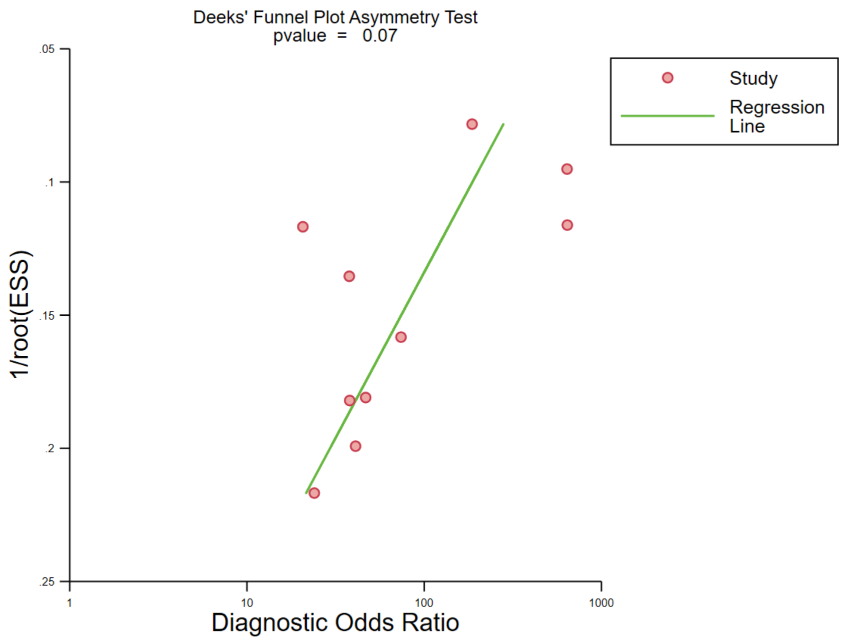

Funnel plots suggested there was no publication bias.

Supplementary Figure 33. Publication bias using ^{18}F-FDG PET imaging based on sample size (PD vs. AP)

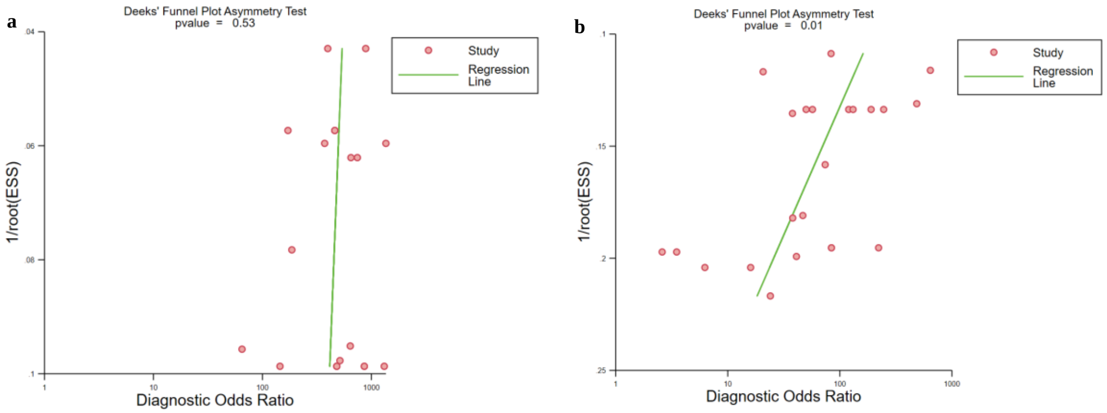

a. Sample size ≥ 100 (Funnel plots suggested there was no publication bias).

b. Sample size < 100 (Funnel plots suggested there was a publication bias).

Supplementary Figure 34. QUADAS-AI summary plot and risk of bias and concern of applicability for each item

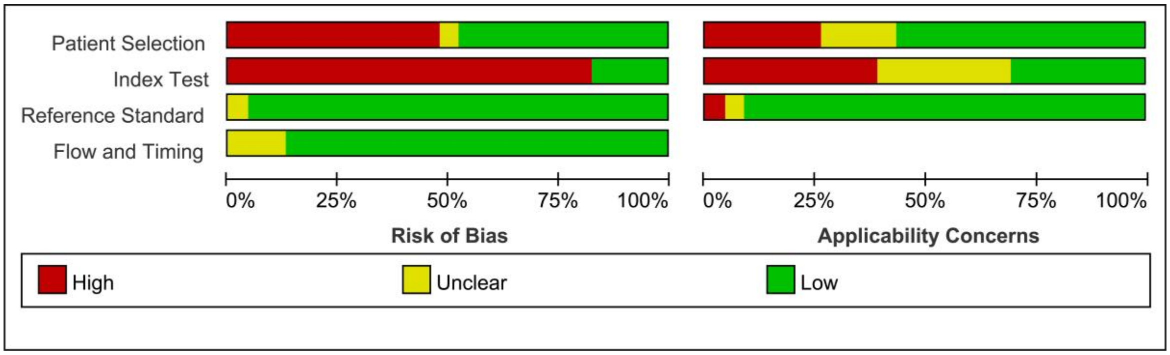

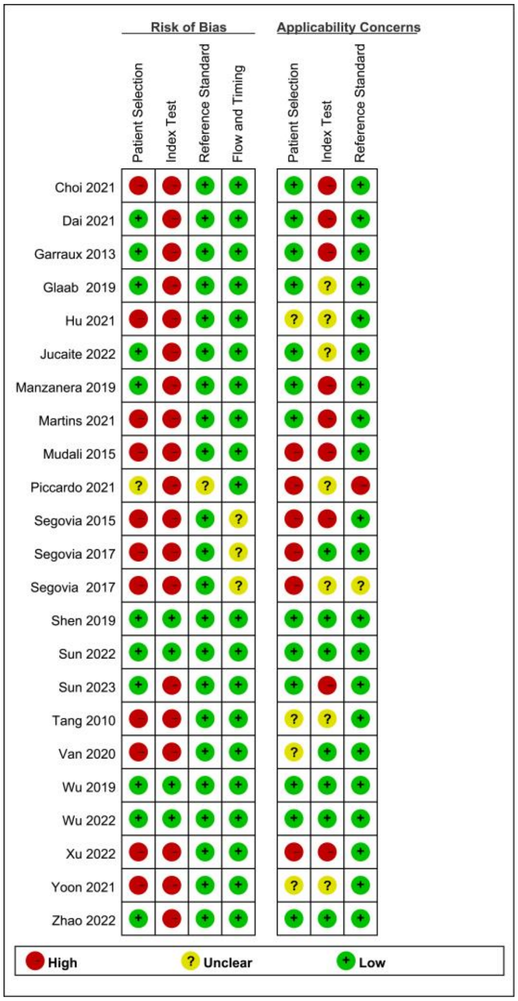

Supplementary Methods. Search terms and search strategy

We show the search strategy for 1) Ovid MEDLINE, 2) Ovid Embase, 3) Cochrane Central, 4) Web of science Core Collection, 5) IEEE Xplore

**Database**	**Search strategy**
Ovid MEDLINE(R) ALL <1946 to August 17, 2023>	1 exp Artificial intelligence/ 177323<br>2 exp machine learning/ 59227<br>3 exp deep learning/ 16255<br>4 exp Neural Networks, Computer/ 60151<br>5 exp Supervised Machine Learning/ 11699<br>6 Random Forest.ab,hw,kf,ti,nm. 20269<br>7 exp Decision Trees/ 12397<br>8 exp Unsupervised Machine Learning/ 814<br>9 exp Support Vector Machine/ 10079<br>10 Vector Machine.ab,hw,kf,ti,nm. 24483<br>11 extreme learning machine.ab,hw,kf,ti,nm. 1125<br>12 linear discriminant.ab,hw,kf,ti,nm. 8557<br>13 logistic regression.ab,hw,kf,ti,nm. 400295<br>14 Convolution neural network.ab,hw,kf,ti,nm. 1368<br>15 Artificial neural network.ab,hw,kf,ti,nm. 11523<br>16 Naive bayes.ab,hw,kf,ti,nm. 3197<br>17 "lasso*".ab,hw,kf,ti,nm. 14868<br>18 "kernel*".ab,hw,kf,ti,nm. 25973<br>19 "elastic net*".ab,hw,kf,ti,nm. 3106<br>20 learning machine.ab,hw,kf,ti,nm. 1408<br>21 1 or 2 or 3 or 4 or 5 or 6 or 7 or 8 or 9 or 10 or 11 or 12 or 13 or 14 or 15 or 16 or 17 or 18 or 19 or 20 649779<br>22 exp Diagnosis, Computer-Assisted/ 86823<br>23 exp Mass Screening/ 144161

	24 "diagnos*".ab,hw,kf,ti,nm. 3479381
	25 "screen*".ab,hw,kf,ti,nm. 1059378
	26 "classifi*".ab,hw,kf,ti,nm. 778949
	27 "discriminat*".ab,hw,kf,ti,nm. 320568
	28 "discriminat*".ab,hw,kf,ti,nm. 320568
	29 22 or 23 or 24 or 25 or 26 or 27 or 28 5122641
	30 exp "Sensitivity and Specificity"/ 648624
	31 exp ROC Curve/ 71299
	32 exp Area Under Curve/ 45730
	33 exp Calibration/ 41185
	34 performance.ab,hw,kf,ti,nm. 1376447
	35 discrimination.ab,hw,kf,ti,nm. 177381
	36 "calibrat*".ab,hw,kf,ti,nm. 151204
	37 "accurac*".ab,hw,kf,ti,nm. 565286
	38 sensitivity.ab,hw,kf,ti,nm. 1351923
	39 specificity.ab,hw,kf,ti,nm. 1157747
	40 AUC.ab,hw,kf,ti,nm. 115449
	41 ROC.ab,hw,kf,ti,nm.130628
	42 30 or 31 or 32 or 33 or 34 or 35 or 36 or 37 or 38 or 39 or 40 or 41 3937810
	43 exp Positron-Emission Tomography/ 79498
	44 exp Positron Emission Tomography Computed Tomography/ 19386
	45 "PET*".ab,hw,kf,ti,nm. 249526
	46 "PET image*".ab,hw,kf,ti,nm. 6299
	47 PET scan.ab,hw,kf,ti,nm. 4841
	48 PET neuroimaging.ab,hw,kf,ti,nm. 198
	49 43 or 44 or 45 or 46 or 47 or 48 269246
	50 exp Parkinson Disease/ 82302
	51 exp Parkinsonian Disorders/ 98128

	52 Idiopathic Parkinson's disease.ab,hw,kf,ti,nm. 3199<br>53 Parkinsonism.ab,hw,kf,ti,nm. 20469<br>54 atypical Parkinson's syndromes.ab,hw,kf,ti,nm.6<br>55 Progressive supranuclear palsy.ab,hw,kf,ti,nm. 4920<br>56 Multiple system atrophy.ab,hw,kf,ti,nm. 5463<br>57 50 or 51 or 52 or 53 or 54 or 55 or 56 110641<br>58 49 and 57 3203<br>59 29 and 58 1243<br>60 21 and 59 49<br>61 42 and 60 41
Ovid Embase <1974 to 2023 August 17>	1 exp Artificial intelligence/ 83988<br>2 exp machine learning/ 409038<br>3 exp deep learning/ 42873<br>4 exp Neural Networks, Computer/ 92413<br>5 exp Supervised Machine Learning/ 4324<br>6 Random Forest.ab,hw,kf,ti. 31344<br>7 exp Decision Trees/ 21630<br>8 exp Unsupervised Machine Learning/ 2587<br>9 exp Support Vector Machine/ 39429<br>10 Vector Machine.ab,hw,kf,ti. 44333<br>11 extreme learning machine.ab,hw,kf,ti. 1689<br>12 linear discriminant.ab,hw,kf,ti. 10496<br>13 logistic regression.ab,hw,kf,ti. 599897<br>14 Convolution neural network.ab,hw,kf,ti. 1668<br>15 Artificial neural network.ab,hw,kf,ti. 54043<br>16 Naive bayes.ab,hw,kf,ti. 3945<br>17 "lasso*".ab,hw,kf,ti. 17638<br>18 "kernel*".ab,hw,kf,ti. 33169

	19 "elastic net*".ab,hw,kf,ti. 4160<br>20 learning machine.ab,hw,kf,ti. 4324<br>21 1 or 2 or 3 or 4 or 5 or 6 or 7 or 8 or 9 or 10 or 11 or 12 or 13 or 14 or 15 or 16 or 17 or 18 or 19 or 20 1077440<br>22 exp Diagnosis, Computer-Assisted/ 1436290<br>23 exp Mass Screening/308516<br>24 "diagnos*".ab,hw,kf,ti. 5824937<br>25 "screen*".ab,hw,kf,ti. 1706592<br>26 "classifi*".ab,hw,kf,ti. 1432364<br>27 "discriminat*".ab,hw,kf,ti. 410716<br>28 "discriminat*".ab,hw,kf,ti. 410716<br>29 22 or 23 or 24 or 25 or 26 or 27 or 28 8972111<br>30 exp "Sensitivity and Specificity"/ 484836<br>31 exp ROC Curve/ 207003<br>32 exp Area Under Curve/ 197557<br>33 exp Calibration/ 93538<br>34 performance.ab,hw,kf,ti. 2038256<br>35 discrimination.ab,hw,kf,ti. 227683<br>36 "calibrat*".ab,hw,kf,ti. 194698<br>37 "accurac*".ab,hw,kf,ti. 1137372<br>38 sensitivity.ab,hw,kf,ti. 1806398<br>39 specificity.ab,hw,kf,ti. 1080035<br>40 AUC.ab,hw,kf,ti. 183330<br>41 ROC.ab,hw,kf,ti. 144587<br>42 30 or 31 or 32 or 33 or 34 or 35 or 36 or 37 or 38 or 39 or 40 or 41 5071408<br>43 exp Positron-Emission Tomography/ 228819<br>44 exp Positron Emission Tomography Computed Tomography/ 62478<br>45 "PET*".ab,hw,kf,ti. 412168

	46 "PET image*".ab,hw,kf,ti. 13032<br>47 PET scan.ab,hw,kf,ti. 13950<br>48 PET neuroimaging.ab,hw,kf,ti. 369<br>49 43 or 44 or 45 or 46 or 47 or 48 502682<br>50 exp Parkinson Disease/ 190765<br>51 exp Parkinsonian Disorders/ 34688<br>52 Idiopathic Parkinson's disease.ab,hw,kf,ti. 4862<br>53 Parkinsonism.ab,hw,kf,ti. 44838<br>54 atypical Parkinson's syndromes.ab,hw,kf,ti. 10<br>55 Progressive supranuclear palsy.ab,hw,kf,ti. 9820<br>56 Multiple system atrophy.ab,hw,kf,ti. 8082<br>57 50 or 51 or 52 or 53 or 54 or 55 or 56 223992<br>58 49 and 57 9487<br>59 29 and 58 8223<br>60 21 and 59 280<br>61 42 and 60 174
Cochrane Central Register of Controlled Trials Issue 7 of 12, July 2023	#1 MeSH descriptor: [Artificial Intelligence] explode all trees 2908<br>#2 MeSH descriptor: [Machine Learning] explode all trees 911<br>#3 MeSH descriptor: [Deep Learning] explode all trees 276<br>#4 MeSH descriptor: [Neural Networks, Computer] explode all trees 533<br>#5 MeSH descriptor: [Supervised Machine Learning] explode all trees 104<br>#6 (Random forest):ti,ab,kw OR (extreme learning machine):ti,ab,kw OR (linear discriminant):ti,ab,kw OR (logistic regression):ti,ab,kw OR (Convolution neural network):ti,ab,kw 26203<br>#7 MeSH descriptor: [Decision Trees] explode all trees 325<br>#8 MeSH descriptor: [Unsupervised Machine Learning] explode all trees 5<br>#9 (Artificial neural network):ti,ab,kw OR (Naive bayes):ti,ab,kw OR (lasso*):ti,ab,kw OR (kernel*):ti,ab,kw OR (elastic net*):ti,ab,kw 1921

	#10 (learning machine):ti,ab,kw OR (Vector Machine):ti,ab,kw 2780<br>#11 #1 or #2 or #3 or #4 or #5 or #6 or #7 or #8 or #9 or #10 31764<br>#12 MeSH descriptor: [Diagnosis, Computer-Assisted] explode all trees 2200<br>#13 MeSH descriptor: [Mass Screening] explode all trees 5361<br>#14 (diagnos*):ti,ab,kw OR (screen*):ti,ab,kw OR (classifi*):ti,ab,kw OR (discriminat*):ti,ab,kw OR (monitor*):ti,ab,kw500720<br>#15 #12 or #13 or #14 501201<br>#16 MeSH descriptor: [Sensitivity and Specificity] explode all trees 19931<br>#17 MeSH descriptor: [ROC Curve] explode all trees 1687<br>#18 MeSH descriptor: [Calibration] explode all trees 520<br>#29 MeSH descriptor: [Area Under Curve] explode all trees 7900<br>#20 (performance):ti,ab,kw OR (discrimination):ti,ab,kw OR (calibrat*):ti,ab,kw OR (accurac*):ti,ab,kw OR (sensitivity):ti,ab,kw 207327<br>#21 (specificity):ti,ab,kw OR (ROC):ti,ab,kw OR (AUC):ti,ab,kw 46950<br>#22 #16 or #17 or #18 or #19 or #20 or #21 237953<br>#23 MeSH descriptor: [Positron-Emission Tomography] explode all trees 1449<br>#24 MeSH descriptor: [Positron Emission Tomography Computed Tomography] explode all trees 299<br>#25 (PET*):ti,ab,kw OR (PET image*):ti,ab,kw OR (PET scan):ti,ab,kw OR (PET neuroimaging):ti,ab,kw 17659<br>#26 #23 or #24 or #25 18023<br>#27 MeSH descriptor: [Parkinson Disease] explode all trees 6187<br>#28 MeSH descriptor: [Parkinsonian Disorders] explode all trees 6450<br>#29 (Idiopathic Parkinson's disease):ti,ab,kw OR (Parkinsonism):ti,ab,kw OR (atypical Parkinson's syndromes):ti,ab,kw OR (Progressive supranuclear palsy):ti,ab,kw OR (Multiple system atrophy):ti,ab,kw 3096<br>#30 #27 or #28 or #29 8408<br>#31 #11 and #15 and #22 and #26 and #30 3

Web of science Core Collection=SCI-EXPANDED, SSCI, A&HCI, CPCI-S, CPCI-SSH, BKCI-S, BKCI-SSH, ESCI, CCR-EXPANDED, IC Timespan=All years	#5 AND #4 AND #3 AND #2 AND #1 (42)<br>1. TS= “Artificial intelligence” OR “machine learning” OR “deep learning” OR “Neural Networks, Computer” OR “Supervised Machine Learning” OR “Random forest” OR “Decision Trees” OR “Unsupervised Machine Learning” OR “Support Vector Machine” OR “extreme learning machine” OR “linear discriminant” OR “logistic regression” OR “Convolution neural network” OR “Artificial neural network” OR “Naive bayes” OR “lasso*” R “kernel*” OR “elastic net*” OR “Vector Machine” OR “Learning machine” (1,251,708)<br>2. TS= “Diagnosis, Computer-Assisted” OR “Mass Screening” OR “diagnos*” OR “screen*” OR “classifi*” OR “discriminat*” OR “monitor*” (7,437,004)<br>3. TS= “Sensitivity and Specificity” OR “ROC Curve” OR “Calibration” OR “performance” OR “Area Under Curve” OR “discrimination” OR “calibrat*” OR “accurac*” OR “sensitivity” OR “specificity” OR “ROC” OR “AUC” (9,276,351)<br>4. TS= “Positron-Emission Tomography” OR “Positron Emission Tomography Computed Tomography” OR “PET*” OR “PET image*” OR “PET scan” OR “PET neuroimaging” (707,969)<br>5. TS= “Parkinson Disease” OR “Parkinsonian Disorders” OR “Idiopathic Parkinson's disease” OR “Parkinsonism” OR “atypical Parkinson's syndromes” OR “Progressive supranuclear palsy” OR “Multiple system atrophy” (54,472)
IEEE Xplore	((“Abstract”: “Artificial intelligence” OR “machine learning” OR “deep learning” OR “Neural Networks” OR “Supervised Machine Learning” OR “Random forest” OR “Decision Trees” OR “Vector Machine” OR “learning machine”) AND (“Abstract”: roc OR auc OR performance OR discriminat* OR calibrat* OR accuracy* OR sensitivity OR specificity) AND (“Abstract”: “Parkinson Disease” OR “Parkinsonian Disorders” OR “Idiopathic Parkinson's disease” OR “Parkinsonism” OR “Parkinson’s Disease”) AND (“Abstract”: “Positron-Emission Tomography” OR “Positron Emission Tomography Computed Tomography” OR PET* OR “PET neuroimaging”)) (10)

Supplementary Methods. Inclusion/exclusion criteria of literature

PICOS	Inclusion	Exclusion
P	Patients with Parkinson's disease that underwent clinical diagnosis were included in our study	Patients with brain parkinsonism (e.g., depression, traumatic brain injury, cancer, etc.).
I	1) patients in the case- or experimental group received Artificial intelligence to assist the classification based on the PET imaging;<br>2) no limit on sample size.	Using other medical imaging approach but not PET imaging was excluded (e.g., SPECT, MRI/fMRI).
C	Patients in the control groups underwent clinical diagnosis or other approach.	Did not differentiate between AI-assisted PET imaging and clinical diagnosis were not eligible. studies that do not involve fully automated AI or a classification task.
O	Primary outcomes including accuracy of patients.	Relevant outcomes were missing.
S	RCTs, prospective cohort studies, and retrospective comparative studies in our analysis.	1) Articles without peer-reviewed or unpublished;<br>2) Quasi-experimental studies and crossover studies.

Supplementary References

1 Sun, X. *et al.* Use of deep learning-based radiomics to differentiate Parkinson's disease patients from normal controls: a study based on [(18)F]FDG PET imaging. *Eur Radiol* **32**, 8008-8018, doi:10.1007/s00330-022-08799-z (2022).

2 Yoon, H. J. *et al.* Heterogeneity by global and textural feature analysis in F-18 FP-CIT brain PET images for diagnosis of Parkinson's disease. *Medicine (Baltimore)* **100**, e26961, doi:10.1097/md.0000000000026961 (2021).

3 Piccardo, A. *et al.* The role of the deep convolutional neural network as an aid to interpreting brain [(18)F]DOPA PET/CT in the diagnosis of Parkinson's disease. *Eur Radiol* **31**, 7003-7011, doi:10.1007/s00330-021-07779-z (2021).

4 Martins, R. *et al.* Automatic classification of idiopathic Parkinson's disease and atypical Parkinsonian syndromes combining [(11)C]raclopride PET uptake and MRI grey matter morphometry. *J Neural Eng* **18**, doi:10.1088/1741-2552/abf772 (2021).

5 Dai, Y. *et al.* Multi-Focus Image Fusion Based on Convolution Neural Network for Parkinson's Disease Image Classification. *Diagnostics (Basel)* **11**, doi:10.3390/diagnostics11122379 (2021).

6 van Veen, R. *et al.* An application of generalized matrix learning vector quantization in neuroimaging. *Comput Methods Programs Biomed* **197**, 105708, doi:10.1016/j.cmpb.2020.105708 (2020).

7 Wu, Y. *et al.* Use of radiomic features and support vector machine to distinguish Parkinson's disease cases from normal controls. *Ann Transl Med* **7**, 773, doi:10.21037/atm.2019.11.26 (2019).

8 Shen, T. *et al.* Use of Overlapping Group LASSO Sparse Deep Belief Network to Discriminate Parkinson's Disease and Normal Control. *Front Neurosci* **13**, 396, doi:10.3389/fnins.2019.00396 (2019).

9 Manzanera, O. M. *et al.* Scaled Subprofile Modeling and Convolutional Neural Networks for the Identification of Parkinson's Disease in 3D Nuclear Imaging Data. *Int J Neural Syst* **29**, 1950010, doi:10.1142/s0129065719500102 (2019).

10 Glaab, E. *et al.* Integrative analysis of blood metabolomics and PET brain neuroimaging data for Parkinson's disease. *Neurobiol Dis* **124**, 555-562, doi:10.1016/j.nbd.2019.01.003 (2019).

11 Mudali, D., Teune, L. K., Renken, R. J., Leenders, K. L. & Roerdink, J. B. Classification of Parkinsonian syndromes from FDG-PET brain data using decision trees with SSM/PCA features. *Comput Math Methods*

Med **2015**, 136921, doi:10.1155/2015/136921 (2015).

12 Sun, J. *et al.* Identification of Parkinson's disease and multiple system atrophy using multimodal PET/MRI radiomics. *Eur Radiol*, doi:10.1007/s00330-023-10003-9 (2023).

13 Wu, P. *et al.* Differential Diagnosis of Parkinsonism Based on Deep Metabolic Imaging Indices. *J Nucl Med* **63**, 1741-1747, doi:10.2967/jnumed.121.263029 (2022).

14 Zhao, Y. *et al.* Decoding the dopamine transporter imaging for the differential diagnosis of parkinsonism using deep learning. *Eur J Nucl Med Mol Imaging* **49**, 2798-2811, doi:10.1007/s00259-022-05804-x (2022).

15 Xu, J. *et al.* Computer-Aided Classification Framework of Parkinsonian Disorders Using (11)C-CFT PET Imaging. *Front Aging Neurosci* **13**, 792951, doi:10.3389/fnagi.2021.792951 (2021).

16 Hu, X. *et al.* Multivariate radiomics models based on (18)F-FDG hybrid PET/MRI for distinguishing between Parkinson's disease and multiple system atrophy. *Eur J Nucl Med Mol Imaging* **48**, 3469-3481, doi:10.1007/s00259-021-05325-z (2021).

17 Choi, B. W. *et al.* Faster Region-Based Convolutional Neural Network in the Classification of Different Parkinsonism Patterns of the Striatum on Maximum Intensity Projection Images of [(18)F]FP-CIT Positron Emission Tomography. *Diagnostics (Basel)* **11**, doi:10.3390/diagnostics11091557 (2021).

18 Rus, T. *et al.* Differential diagnosis of parkinsonian syndromes: a comparison of clinical and automated - metabolic brain patterns' based approach. *Eur J Nucl Med Mol Imaging* **47**, 2901-2910, doi:10.1007/s00259-020-04785-z (2020).

19 Segovia, F., Górriz, J. M., Ramírez, J., Martínez-Murcia, F. J. & Salas-Gonzalez, D. Preprocessing of (18)F-DMFP-PET Data Based on Hidden Markov Random Fields and the Gaussian Distribution. *Front Aging Neurosci* **9**, 326, doi:10.3389/fnagi.2017.00326 (2017).

20 Segovia, F. *et al.* Multivariate Analysis of (18)F-DMFP PET Data to Assist the Diagnosis of Parkinsonism. *Front Neuroinform* **11**, 23, doi:10.3389/fninf.2017.00023 (2017).

21 Segovia, F. *et al.* Distinguishing Parkinson's disease from atypical parkinsonian syndromes using PET data and a computer system based on support vector machines and Bayesian networks. *Front Comput Neurosci* **9**, 137, doi:10.3389/fncom.2015.00137 (2015).

22 Garraux, G. *et al.* Multiclass classification of FDG PET scans for the distinction between Parkinson's disease and atypical parkinsonian syndromes. *Neuroimage Clin* **2**, 883-893, doi:10.1016/j.nicl.2013.06.004 (2013).

23 Tang, C. C. *et al*. Differential diagnosis of parkinsonism: a metabolic imaging study using pattern analysis. *Lancet Neurol* **9**, 149-158, doi:10.1016/s1474-4422(10)70002-8 (2010).
